# LINC00941 Promotes Cell Malignant Behavior and Is One of Five Costimulatory Molecule-Related lncRNAs That Predict Prognosis in Renal Clear Cell Carcinoma

**DOI:** 10.3390/medicina59020187

**Published:** 2023-01-17

**Authors:** Huafeng Pan, Wei Wei, Guanghou Fu, Jiaren Pan, Baiye Jin

**Affiliations:** 1 Department of Urology, The First Affiliated Hospital, Zhejiang University School of Medicine, Hangzhou 310003, China; 2 Department of Urology, Hwa Mei Hospital, University of Chinese Academy of Sciences, Ningbo 315010, China; 3 Ningbo NO. 2 Hospital, Ningbo 315099, China

**Keywords:** renal cancer, costimulatory molecule, lncRNAs, immune, immunotherapy

## Abstract

*Background and Objectives***:** A significant role was played by costimulatory molecules in renal cancer. However, the lncRNAs regulating costimulatory molecules have not been fully investigated. *Materials and Methods***:** Data from the next-sequence file and clinical data were downloaded from the Cancer Genome Atlas (TCGA) database. All analyses were conducted using the R and GraphPad Prism software. *Results:* A total of 1736 costimulatory molecule-related lncRNAs were determined under the threshold of |Cor| > 0.5 and *p*-value < 0.001. Furthermore, a prognosis prediction signature consisting of five lncRNAs: LINC00941, AC016773.1, AL162171.1, HOTAIRM1, and AL109741.1 was established with great prediction ability. By combining risk score and clinical parameters, a nomogram plot was constructed for better clinical practice. A biological enrichment analysis indicated that E2F targets, coagulation, IL6/JAK/STAT3 signaling, G2/M checkpoint, and allograft rejection pathways were activated in high-risk patients. Furthermore, a higher infiltration level of resting CD4+ T cell, M2 macrophage, and resting mast cells, while a lower CD8+ T cell infiltration was observed in high-risk patients. It is worthy of note that, low-risk patients might respond better to PD-1 checkpoint therapy. A correlation analysis of LINC00941 revealed that it was positively correlated with Th2 cells, Th1 cells, macrophages, and Treg cells, but negatively correlated with Th17 cells. A pathway enrichment analysis indicated that the pathways of the inflammatory response, G2M checkpoint, and IL6/JAK/STAT3 signaling were significantly activated in patients with high LINC00941 expression. In vitro experiments indicated that LINC00941 can enhance the malignant biological behaviors of renal cancer cells. *Conclusions*: Our study established a costimulatory molecule-related lncRNAs-based prognosis model with a great prediction prognosis. In addition, LINC00941 could enhance the malignant biological behaviors of renal cancer cells.

## 1. Introduction

In 2020, there were approximately 420,000 new cases of renal cell carcinoma (RCC) and 180,000 deaths from it globally [1]. Among RCCs, clear cell renal cell carcinoma (ccRCC) is the most common, characteristic of a worse prognosis and high mortality [2]. While many promising treatments have been proposed for ccRCC patients, recurrence or metastasis rates after surgery still exceed 20% [3]. Moreover, due to the insidious onset of symptoms, a considerable proportion of ccRCC patients have progressed to an advanced stage by the time of their first diagnosis, thus leading to the loss of the opportunity for surgery [4]. Consequently, the identification of effective molecular biomarkers for ccRCC therapy is still imperative.

Costimulatory molecules, consisting of the B7-CD28 family and tumor necrosis factor (TNF) family, play a critical role in cancer biology [5]. Cell-mediated immunity is triggered by molecules from the B7/CD28 family, including the most commonly observed PD-1/L1 axis. Meanwhile, members of the TNF/TNFR family are engaged in the later phases of T-cell activation, as well as antitumor immunity [6]. In ccRCC, however, few studies have been conducted on costimulation molecules and their biological functions. Long noncoding RNA (lncRNA) has broad regulatory effects and participates in several stages of cancer [7]. For instance, Li et al. revealed that the MRCCAT1 was overexpressed in ccRCC tissue and could promote cancer metastasis [8]. Given the prominent values of costimulatory molecules and lncRNA, it is essential to identify the costimulatory molecule-related lncRNAs (CMLs) affecting the prognosis, which might be useful treatment options and prognosis evaluations in ccRCC patients.

Increasing amounts of public data have been generated in the era of Big Data, and secondary analysis of these data can provide researchers with direction [9]. Here, we comprehensively investigated the role of CMLs in ccRCC. A prognosis signature consisting of LINC00941, AC016773.1, AL162171.1, HOTAIRM1, and AL109741.1 was established with great prediction efficiency. We then investigated the underlying biological difference in patients with high and low risk scores, including immune and pathway enrichment analysis. It is worthy of note that low-risk patients might respond better to PD-1 checkpoint therapy, which might be helpful for clinical treatment options. Finally, LINC00941 was selected for further research. The results showed that LINC00941 could enhance the malignant biological behaviors of renal cancer cells., which might be a potential biomarker of ccRCC.

## 2. Methods

### 2.1. Data Collection

Data from the next-sequence file and clinical data were downloaded from the Cancer Genome Atlas (TCGA) database of the TCGA-KIRC project (72 normal tissue and 539 tumor tissue). The original form of expression profile data was the “STAR-count” form. All the data were preprocessed before analysis. The detailed clinicopathological parameters of patients enrolled in our analysis are shown in Table 1. The collected list of costimulatory molecules is shown in Table S1 [10].

### 2.2. Identification of CMLs

A correlation analysis was utilized to identify the relationship between costimulatory molecules and lncRNAs data. The lncRNAs were considered as CMLs meeting the criteria of |Cor| > 0.5 and *p*-value < 0.001.

### 2.3. CMLs Prognosis Analysis

For the enrolled patients, a 1:1 ratio was used to randomly assign patients to training and validation groups. First, using a Univariate Cox regression analysis, CMLs associated with patient survival were identified. (*p* value < 0.05). Following this, the Random Survival Forests Variable Hunting (RSFVH) algorithm was utilized to identify lncRNAs. Additionally, independent prognostic factors associated with CML survival were determined by a multivariate Cox regression analysis. The formula of the risk score consisting of five CMLs was as follows:

(1) $$riskscore=\overset{n}{\underset{i=1}{\sum }}coef\left(lncRNAi\right)*\exp\left(lncRNAi\right)$$

The survival difference between the two groups was compared using the Kaplan-Meier (KM) survival curve. In the training and validation cohorts, the median value of the risk score in each cohort was defined as the cut-off values to distinguish high- and low-risk groups. The patients with a risk score higher than the median value were high-risk patients, otherwise, they were considered to be low-risk patients.

### 2.4. Development of a Nomogram

Using the rms package, a nomogram was established for better application in the clinic. Furthermore, the calibration curve for 3-, 5-, and 8 years was performed to compare the predictive accuracies of the nomogram.

### 2.5. Biological Enrichment

To investigate the significant differences between the two risk groups, a gene set enrichment analysis (GSEA) was performed. All annotated gene set files (*n* = 9) retrieved from the MSigDB database were chosen as the gene set of reference.

### 2.6. Immune Features and Immunotherapy Response Prediction

The proportions of 22 different types of infiltrating immune cells in the tumor microenvironment were quantified using the CIBERSORT algorithm [11]. Each patient’s immune-related function was quantified using the single sample gene set enrichment analysis (ssGSEA) algorithm [12]. Tumor Immune Dysfunction and Exclusion (TIDE) and submap algorithms were utilized to evaluate the immunotherapy responses between the two risk groups [13]. For the TIDE algorithm, all patients were assigned a TIDE score based on their expression profile data through the online portal website http://tide.dfci.harvard.edu/, accessed on 21 June 2022. The patients whose TIDE score > 0 were regarded as immunotherapy non-responders; otherwise, they were considered to be responders. The submap algorithm can evaluate the patient’s response to two specific immunotherapy options, CTLA4 and PD-1 therapy. The submap algorithm was conducted through the GenePattern server (https://cloud.genepattern.org/, accessed on 23 June 2022). The method for *p* value correction was “Bonferroni” and the output Bonferroni corrected *p* value could reflect the similarity between selected patients and specific immunotherapy therapy cohorts (PD1-R = response of PD-1 therapy, PD1-nonR = non-response of PD-1 therapy, CTLA4-R = response of CTLA4 therapy, CTLA4-nonR = non-response of CTLA4 therapy), in which Bonferroni corrected *p* value < 0.05 was considered as statistically significant.

### 2.7. Cell Lines and qRT-Pcr Assay

Normal renal epithelial cell lines (HK-2) and renal cancer cell lines (786-O, Caki-1, Caki-2) were purchased from the Cell Bank of Shanghai Academy of Chinese Sciences and routinely maintained at our laboratory. Total RNA was extracted using an RNA extraction kit (Qiagen). The sequences of primers used in the study were as follows: LINC00941: forward primer, 5′-CAAGCAACCGTCCAACTACCAGACA-3′; reverse primer, 5′-AAATCAAGAGCCCAAACATTGTGAA-3′; GAPDH, forward primer, 5′-CTGGGCTACACTGAGCACC-3′, forward primer, 5′-AAGTGGTCGTTGAGGGCAATG-3′.

### 2.8. Cell Transfection

Cell transfection was carried out based on the lipofectamine 3000 regent following the protocol. The control and sh-LINC00941 plasmids were purchased from Shanghai GenePharma, whose target sequences were as follows: sh-LINC#1, 5′-GGACCAACTATGCTTATAA-3′; sh-LINC#2, 5′-GCCCTCGAGAAGTGTCTAA-3′; sh-LINC#3, 5′-GAGCATGTATCCATCTTAT-3′.

### 2.9. Cell Proliferation Assay

Firstly, we resuspended and seeded 500 cells per well in a six-plate well. The cells were then cultured for 14 days according to conventional cell culture conditions. Finally, crystal violet staining was applied after the cells were mixed with formaldehyde. A CCK8 assay was conducted using a CCK8 kit (Dojindo, Shanghai, China) based on the protocol.

### 2.10. Transwell Assays

In a 24-plate well, an 8-um pore Transwell chamber was used to divide the plate into the upper and lower chamber. To upper chamber were added 4 × 10^3^ cells with medium FBS. The lower chamber was filled with a medium containing 20% FBS. Cells were then stained with crystal violet after being mixed with formaldehyde for 24 h.

### 2.11. Statistical Analysis

All statistical analyses were conducted using R software and GraphPad Prism 8. Statistical significance was determined by a two-sided *p*-value less than 0.05. For the variables conforming to a normal distribution, a Student’s t-test was used for analysis; for the variables conforming to non-normal distribution, the Mann-Whitney U test was used for analysis. All biological experiments were repeated three times to obtain a statistical *p* value. The receiver operating characteristic (ROC) curve was utilized to assess the prediction performance of the identified variables. The Area Under the Curve (AUC) value of the ROC curve was calculated using the survivalROC package in R software.

## 3. Results

### 3.1. Construction of CMLs Prognosis Signature

**Figure 1:**
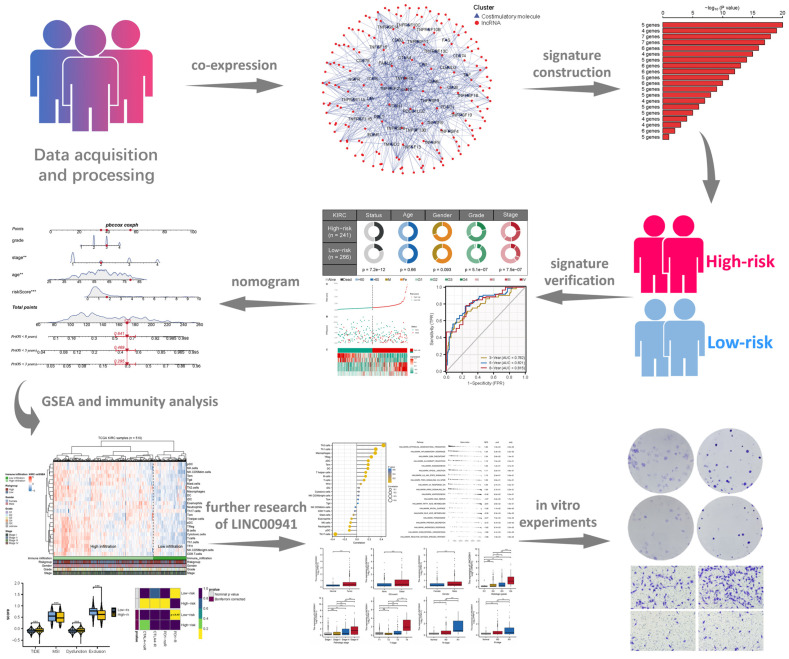
Flow chart. ** = *p* < 0.01, *** = *p* < 0.001.

**Figure 2:**
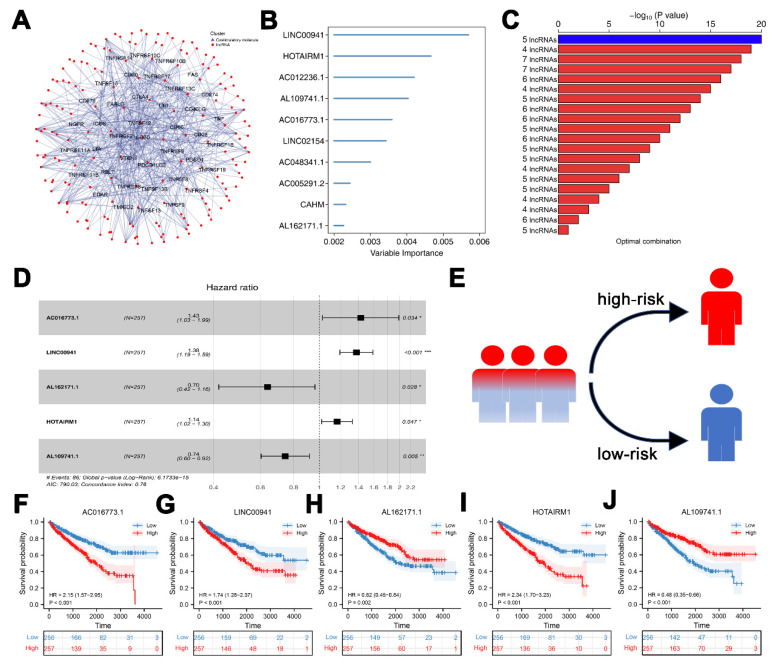
Identification of candidate lncRNAs and development of a molecule-related lncRNA signature. Notes: (**A**) The identification of costimulatory molecule-related lncRNAs. (**B**) A random survival forest analysis screened 10 lncRNAs. (**C**) After Kaplan–Meier analysis of 1023 combinations, the top 20 signatures were sorted according to the *p* value of KM. The signature included five lncRNAs that were screened out, as they had the biggest −log^10^ *p* value. (**D**) The forest plot of 17 prognostic lncRNAs with HR > 5.0 or HR < 0.8, * = *p* < 0.05, ** = *p* < 0.01, *** = *p* < 0.001 (**E**) Patients were divided into high- and low-risk groups according to the median risk score. (**F**–**J**) Kaplan-Meier survival curves of five prognostic costimulatory molecule-related lncRNAs in KIRC.

Figure 1 illustrates a flowchart of the study design. In total, 1736 costimulatory molecule-related lncRNAs were identified after co-expression analysis with |Cor| > 0.5 and *p* value = 0.001 (Figure 2A and Table S2). Based on these CMLs, a univariate Cox regression analysis was performed, identifying 219 lncRNAs strongly associated with survival (Table S3). A random forest algorithm was then used to identify the most optimal lncRNA combination with high variable importance for the signature, among which LINC00941 had the highest importance (Figure 2B,C). Finally, a multivariate Cox regression analysis identified the signature combination consisting of five lncRNAs: LINC00941, AC016773.1, AL162171.1, HOTAIRM1 and AL109741.1 had the most significant *p* value (Figure 2D). The risk score was calculated using the formula “Riskscore = AC016773.1 ∗ 0.356 + LINC00941 ∗ 0.319 + AL162171.1 ∗ −0.359 + HOTAIRM1 ∗ 0.132 + AL109741.1 ∗ −0.296” (Figure 2E). KM survival curves showed that lncRNA AC016773.1, LINC00941, and HOTAIRM1 were the risk factors, yet the AL162171.1 and AL109741.1 were the protective factors (Figure 2F–J).

### 3.2. Validation and Evaluation of the Prognosis Model

**Figure 3:**
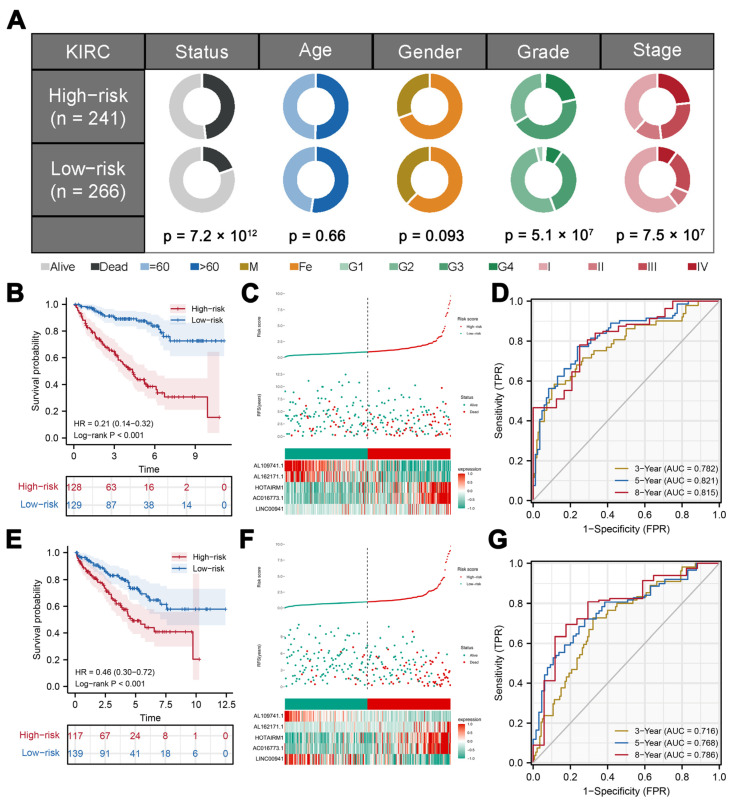
Evaluation and verification of the signature. Notes: (**A**) Pie charts showing the Chi-squared test of clinicopathologic factors between two risk groups in KIRC. (**B**,**E**) Kaplan-Meier curve of the signature prognosis in high- and low-risk groups. (**C**,**F**) The risk plot showed a higher percentage of progressed patients in the high-risk group (training cohort and validation cohort). (**D**,**G**) Time-dependent ROC curve of 3-, 5- and 8-year survival rates(training cohort and validation cohort).

According to clinical correlation, risk score was correlated with worse clinical grade and stage (Figure 3A). According to the KM survival curve, patients with high risk might have a shorter OS (Figure 3B). Meanwhile, high-risk groups had a higher death rate (Figure 3C). Based on our time-dependent ROC analysis, our model can predict patients prognosis satisfactorily (Figure 3D, 3 years AUC = 0.782, 5 years AUC = 0.821, 8 years AUC = 0.815). Validation group results showed the same trend (Figure 3E–G, 3 years AUC = 0.716, 5 years AUC = 0.768, 8 years AUC = 0.786). A univariate and multivariate analysis indicated that risk score could be an effective prognosis marker independent of other clinical features (Figure 3H,I). A univariate Cox regression analysis indicated that risk score could independently affect patients’ prognosis (Figure S1A). The N classification data of patients has many unknowns, so it was not included in the analysis. Considering the high correlation between clinical stage and T, and M classifications, we performed a multivariate Cox regression analysis based on three modules (age, gender, grade and stage/T classification/M classification). The results all indicated that risk score could be an effective prognosis marker independent of other clinical features (Figure S1B,C).

### 3.3. Nomogram

**Figure 4:**
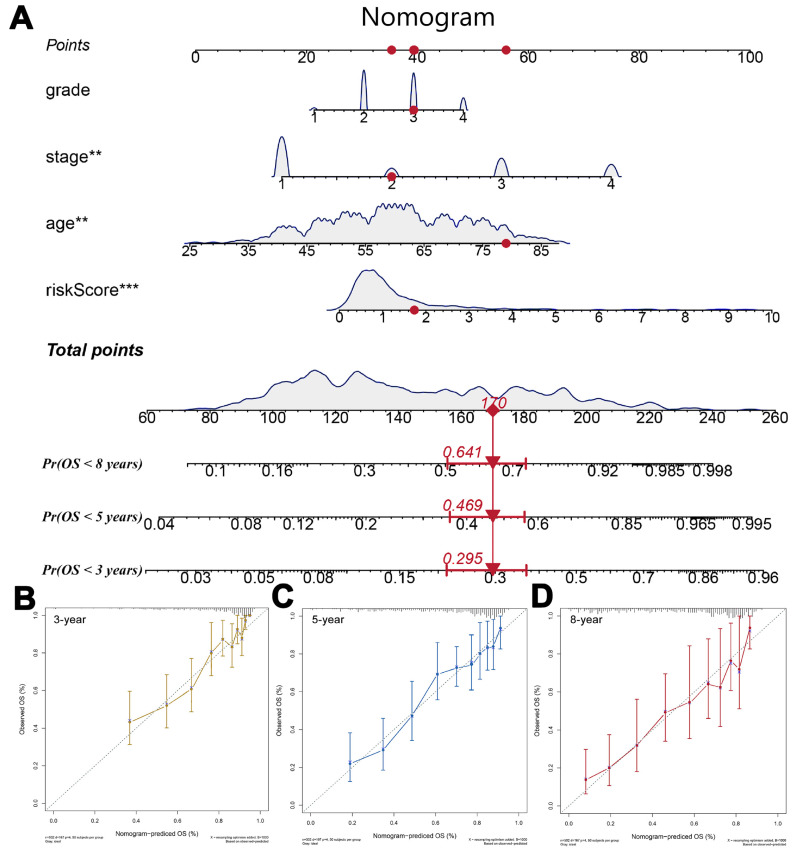
Development and validation of a risk score-based nomogram. Notes: (**A**) The risk score and clinical characteristics of KIRC were used to construct the nomogram, ** = *p* < 0.01, *** = *p* < 0.001. (**B**–**D**) Calibration curves of the nomogram for the estimation of survival rates at 3 (**B**), 5 (**C**) and 8 years (**D**).

For a better application in practice, a nomogram was constructed by combining the risk score and clinical features to predict 3-, 5-, and 8-year OS time, whose C-index was 0.791 (Figure 4A). Furthermore, the calibration plot showed good agreement between the actual observation and predicted survival of 3, 5, 8, and 10 years (Figure 4B–D).

### 3.4. Biological Enrichment

**Figure 5:**
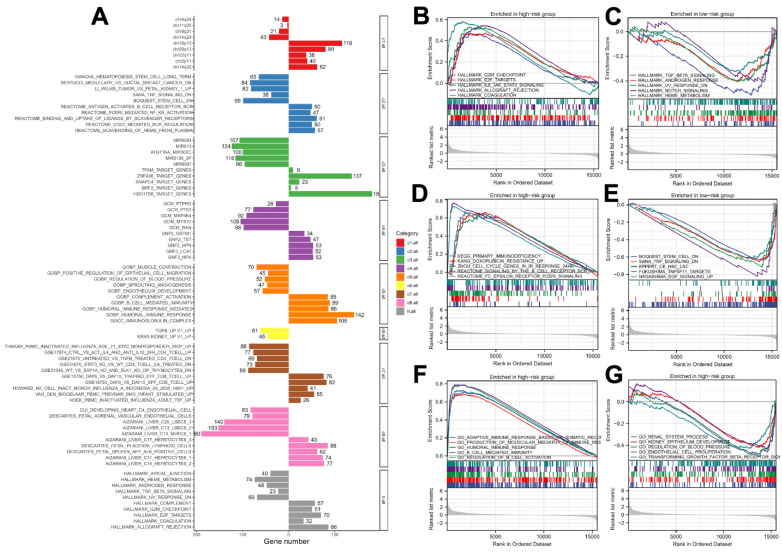
Gene set enrichment analysis for costimulatory molecule-related lncRNA signature. Notes: (**A**) GSEA analysis of the signature based on all annotated gene set files. (**B**,**C**) Different cancer hallmarks are regulated in the high-risk group (**B**) and low-risk group (**C**) of the signature. (**D**,**E**) Associated curated gene sets including KEGG terms enriched in the high-risk (**D**) and low-risk groups (**E**). (**F**,**G**) GO terms enriched in the high-risk (**F**) and low-risk groups (**G**).

A GSEA analysis was utilized to identify the biological difference between high- and low-risk patients (Figure 5A). Results of the GSEA indicated that the terms of E2F targets, coagulation, IL6/JAK/STAT3 signaling, allograft rejection, and G2/M checkpoint were remarkably enriched in high-risk patients, while the top five hallmark terms enriched in low-risk group were TGF-β signaling, androgen response, UV response, Notch signaling and heme metabolism (Figure 5B,C). In addition, the results revealed that the patients in the high-risk group were enriched in primary immune deficiency, cell cycle arrest in response, and doxorubicin resistance, and the low-risk group was related to stem-cell downregulated, TNF signaling (Figure 5D,E). Importantly, the GO terms including renal system process and kidney epithelium development, highly correlated with normal physiological progress of the kidney, were enhanced in the low-risk group, showing a great difference from the high-risk group (Figure 5F,G).

### 3.5. Immune Analysis

**Figure 6:**
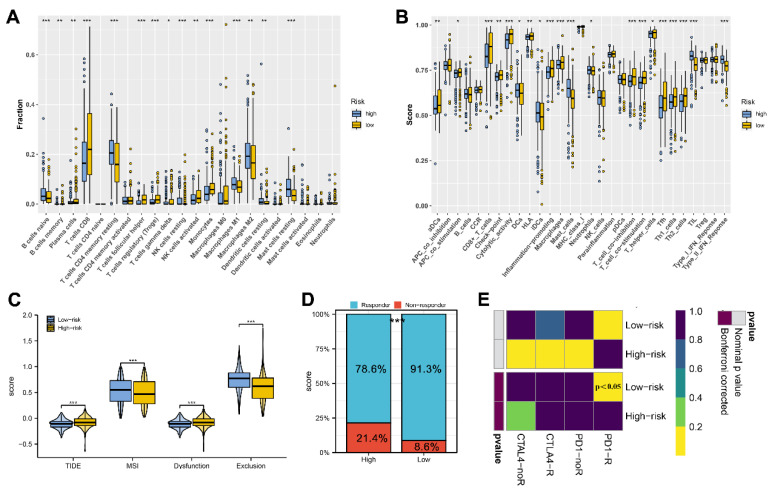
Immune infiltration analysis and immunotherapy response prediction between two risk groups. Notes: (**A**) Comparison of tumor-infiltrating immune cell proportion between the high-risk group and low-risk group. (**B**) Comparison of immune-related functions proportion between the high-risk group and low-risk group. (**C**) Unsupervised clustering of 510 KIRC patients using single-sample gene set enrichment analysis scores from 27 immune cell types. (**D**) Comparisons of TIDE score for chemotherapeutics and targeted therapy of the signature revealed that low-risk patients were more likely to be suitable for immunotherapy. (**E**) Aubmap analysis indicated that KIRC patients in the low-risk group could be more sensitive to the programmed cell death protein 1 inhibitor.

Diverse immune cells and functions infiltrate the tumor microenvironment and regulate the antitumor response. The results of CIBERSORT indicated a higher infiltration level of resting CD4+ T cells, M2 macrophages, and resting mast cells, while a lower infiltration level of CD8+ T cells was noted in high-risk patients (Figure 6A). Meanwhile, the result of an ssGSEA indicated a higher level of immature dendritic cells (iDCs), mast cells, tumor-infiltrating lymphocytes (TIL), and type II IFN response, yet a lower level of activated dendritic cells (aDCs), Th2, CD8+ T cells and Tfh cells was found in high-risk patients (Figure 6B). According to TIDE results, high-risk patients had higher TIDE scores and a lower percentage of immunotherapy responders than patients in low-risk groups (Figure 6C,D). Correspondingly, a subclass analysis found that the Bonferroni corrected *p* value of PD1-R in low-risk patients was less than 0.05, indicating that low-risk patients might respond better to PD-1 therapy (Figure 6E).

### 3.6. Exploring the Effect of LINC00941 in ccRCC

**Figure 7:**
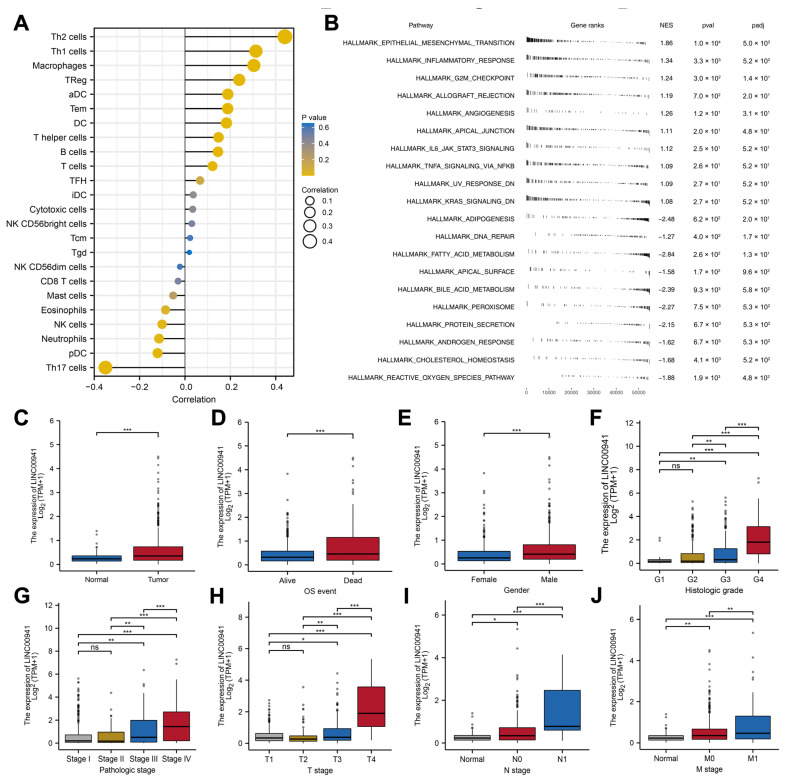
Immunity features and Clinical correlation analysis of LINC00941. Notes: (**A**) Lollipop-diagram showed that LINC00941 has a strong correlation with some immune cells including Th2 cells. (**B**) Pathway enrichment analysis of patients with high and low LINC00941 expression. (**C**–**J**) Box diagram showing that the LINC00941 value was significantly upregulated in the medium or late stages of KIRC, including the Grade 4, Stage IV, T4, N1, and M1 stages.

Considering that the LINC00941 had the most significant *p* value of the multivariate Cox regression and has not been reported in ccRCC previously, we selected it for further analysis to illustrate its role in ccRCC. According to an immune infiltration analysis, LINC00941 was positively correlated with Th2 cells, Th1 cells, macrophages, and Treg, but was negatively correlated with Th17 cells (Figure 7A). GSEA results showed that the pathways of the inflammatory response, IL6/JAK/STAT3 signaling and G2M checkpoint were upregulated in patients with high LINC00941 expression (Figure 7B). A clinical correlation showed that LINC00941 was overexpressed in ccRCC tissue and associated with more progressive clinicopathological parameters, including grade, clinical stage, and TNM classifications (Figure 7C–J).

### 3.7. LINC00941 Promotes Cancer Cell Proliferation, Invasion, and Migration

**Figure 8:**
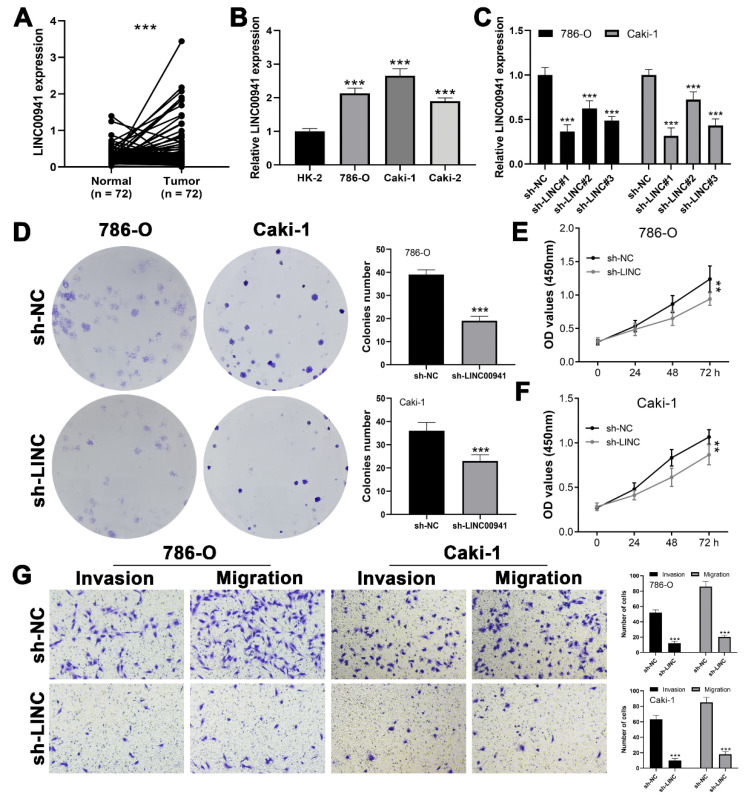
The expression levels of LINC00941 promotes KIRC cell proliferation, invasion, and migration in vitro. Notes: (**A**) LINC00941 expression in paired KIRC tissues, *** = *p* < 0.001. (**B**) LINC00941 mRNA expression in KIRC cell lines and normal renal epithelial cell lines, *** = *p* < 0.001. (**C**) mRNA expression in LINC00941 knockdown cell lines, *** = *p* < 0.001. (**D**) Colony formation assay of 786-O and A498 after the knockdown of LINC00941, *** = *p* < 0.001. (**E**) CCK8 assay of 786-O and A498 after the knockdown of LINC00941, ** = *p* < 0.01. (**G**) The downregulation of LINC00941 reduced the number of migration and invasion cells in the Transwell assay, *** = *p* < 0.001.

We further investigated whether LINC00941 could promote ccRCC cell malignant behavior. A higher level of LINC00941 was noticed in 72 paired ccRCC and adjacent tissues obtained from the TCGA database (Figure 8A). Furthermore, the renal cancer cell lines all showed a higher LINC00941 expression level than the normal HK-2 cell line (Figure 8B). The knockdown efficiency of LINC00941 was then validated (Figure 8C). According to CCK8 and colony formation assays, the inhibition of LINC00941 significantly hampered cancer cell proliferation (Figure 8D–F). In addition, the inhibition of LINC00941 could significantly decrease the invasion and migration ability of cancer cells (Figure 8G).

## 4. Discussion

RCC is still a serious health concern due to its high probability of metastasis and recurrence, in which ccRCC is the most dominant pathologic subtype [14]. Meanwhile, approximately 30% of patients diagnosed with ccRCC have metastasized at the time of their diagnosis because of their insidious symptoms at the time of diagnosis [15]. Thus, identifying new biomarkers associated with ccRCC diagnosis and treatment is important.

In this study, we first identified 1736 CMLs based on a co-expression analysis. Based on a univariate Cox regression analysis, 219 lncRNAs related to prognosis were screened. Furthermore, a CMLs-based prognosis model was established based on five lncRNAs: LINC00941, AC016773.1, AL162171.1, HOTAIRM1, and AL109741.1 through survival forest algorithm and multivariate Cox regression analyses, among which LINC00941 had the highest importance. Both the training and validation cohorts showed a high level of OS prediction efficiency. We further constructed a nomogram based on the risk score and other clinicopathological features to provide a quantitative approach for clinicians. The 3, 5 and 8 years of calibration analysis demonstrated that this nomogram provided accurate survival predictions. Subsequently, a GSEA enrichment analysis showed that metabolism, tumorigenesis, and immune-related functions pathways were highly activated in the high-risk group, suggesting the possible mechanism affecting the cancer progression [16]. Furthermore, patients at high and low risk showed a different pattern in the immune microenvironment. Moreover, we found that low-risk patients might respond better to PD-1 therapy.

On the one hand, emerging studies have corroborated that cancer cells can functionally reprogram the surrounding cells, including the innate immune cells (monocytes and macrophages), affecting the development of various cancer including ccRCC [17]. Our study identified a higher level of TIL, iDCs, tumor-infiltrating mast cells, M2 macrophages, and type II IFN response in high-risk patients, yet a lower level of CD8+ T and Th2 cells. Correspondingly, previous publications by Vuong et al. revealed that there was a correlation between higher levels of TILs identified by morphology and higher recurrence rate in ccRCC [18]. In addition, tumor-infiltrating mast cells could contribute to the evasion of anti-tumor immunity through the release of IL-10 and TGF-β in ccRCC [19]. In addition, the lower CD8+ T cell level in high-risk patients might be one reason for its more progressive clinical status. For tumor-associated macrophages (TAMs), M0 macrophages can differentiate into M1 and M2 macrophages, among which M2 macrophages generally exert a cancer-promoting role [20]. All of these findings in our study about immune cells and status indicated that these members play important roles in the regulation of the ccRCC immune response and might be responsible for the prognosis difference in patients from different risk groups.

On the other hand, a pathway enrichment analysis indicated that the G2/M checkpoints and E2F targets were activated in high-risk groups. In the cell cycle, the G2/M checkpoint is crucial. The abnormal state of the G2/M checkpoint could lead to unrestricted proliferation ability [21]. Most tumors lack tumor suppressor genes, so the upstream G1/S checkpoint is inactivated, allowing them to survive. In the meantime, to protect their genome from disrupting factors, tumor cells including ccRCC rely heavily on the G2/M checkpoint [22]. Consequently, the difference in the G2/M checkpoints pathway in high- and low-risk groups might result in a difference in prognosis. A major function of E2F target genes is to ensure DNA replication and the progression of the cell cycle [23]. There is evidence that many cancer patients have shown poor prognoses when targeting members of the E2F target gene set [24]. These functional pathways suggested the underlying mechanism of prognosis difference between the high- and low-risk groups.

Based on our analysis, LINC00941 had the highest importance in identified CMLs and was therefore selected for further research. LINC00941 has been reported in multiple cancers. For example, in colon cancer, Wu et al. indicated that by preventing the degradation of SMAD4 protein, LINC00941 facilitates cancer metastasis [25]. In papillary thyroid cancers, Gugnoni et al. found that LINC00941 is a TGF-β target gene that could facilitate cancer cell metastasis by regulating CDH6 [26]. Xu et al. revealed that the interaction between LINC00941 and MST1 enhanced glycolysis and the development of pancreatic cancer [27]. Research on LINC00941 in ccRCC, however, is limited. Our study is the first one exploring the role of LINC00941 in ccRCC through bioinformatics analysis and in vitro experiments, which enriched the regulatory effect of LINC00941 in cancer. In fact, plenty of RNA therapeutics are in phase II or III clinical trials [28]. The extensive regulatory and diverse functions of lncRNA provide numerous opportunities for targeted therapeutics, whose effect patterns include transcriptional and post-transcriptional inhibition, protein structural block, steric inhibition, etc. [29]. For example, some preclinical studies have begun to focus on the potential of targeting certain specific lncRNAs to complete tumor therapy and related drug development, such as H19, HOTAIR, LUNAR1, etc. [30]. Based on the patient-derived xenograft models (PDX), HOTAIR [31] and SAMMSON [32] were targeted using siRNAs or ASOs in breast and melanoma models, respectively. Furthermore, studies have shown that the BC819 (also named DTA-H19), a double-stranded DNA plasmid, could cause an anti-tumor effect in a variety of solid tumors under the regulation of H19 gene promoters, which has gotten satisfactory results in phase I/IIa clinical trials in patients with invasive bladder cancer [33]. Furthermore, CRISPR-Cas9 and 3D organoid systems have been used in lncRNA targeting and related studies [34]. In general, the development of lncRNA drugs should be based on a comprehensive understanding of their action mode in diseases. Consequently, we think more studies focused on the specific biological mechanism of LINC00941 in ccRCC are required in the future.

In this study, we successfully constructed a CMLs-based prognosis model and demonstrated the oncogenic role of LINC00941 in ccRCC. However, it invariably has some limitations that need to be addressed. Firstly, the patients enrolled in the analysis were predominantly Caucasian, which introduced a bias to the application of our conclusion to other populations. Secondly, due to the limitations of data, we only performed an internal validation for our survival prediction model based on TCGA data. Thirdly, the TCGA database has no laboratory findings of enrolled patients, which might bring potential bias to the evaluation of the overall condition of the patients. Fourthly, the underlying mechanism of LINC00941 in ccRCC has not been fully explored.

## Figures and Tables

**Table 1:** Clinical features of patients included in our analysis.

Features	Group	Number	Proportion (%)
Age	≤60	262	51.0
	>60	251	49.0
Gender	Female	176	34.3
	Male	337	65.7
Grade	G1	12	2.4
	G2	216	42.7
	G3	201	39.2
	G4	73	14.2
	unknown	8	1.6
Stage	Stage I	255	49.8
	Stage II	56	10.9
	Stage III	117	22.8
	Stage IV	82	16.0
	unknown	3	0.6
T-classification	T1	261	50.8
	T2	68	13.2
	T3	173	33.8
	T4	11	2.2
N-classification	N0	229	44.7
	N1	16	3.1
	unknown	268	52.3
M-classification	M0	407	79.3
	M1	78	15.2
	unknown	28	5.4

## Data Availability

All data analyzed during this study are included in this published article.

## Supplementary Materials

The following supporting information can be downloaded at: https://www.mdpi.com/article/10.3390/medicina59020187/s1, Figure S1. The univariate and multivariate Cox regression analysis of risk score. Table S1. The list of costimulatory molecules. Table S2: The identified 1736 costimulatory molecule-related lncRNAs. Table S3: The identified 219 lncRNAs strongly associated with survival.
