# Supplementary material for: LINC00941 Promotes Cell Malignant Behavior and Is One of Five Costimulatory Molecule-Related lncRNAs That Predict Prognosis in Renal Clear Cell Carcinoma
Source: Medicina (Kaunas). 2023 Jan 17;59(2):187. doi: 10.3390/medicina59020187 (PMC9964476; doi:10.3390/medicina59020187)
Supplement: Supplementary file 1 [file medicina-59-00187-s001.zip › medicina-2101867-supplementary.pdf]

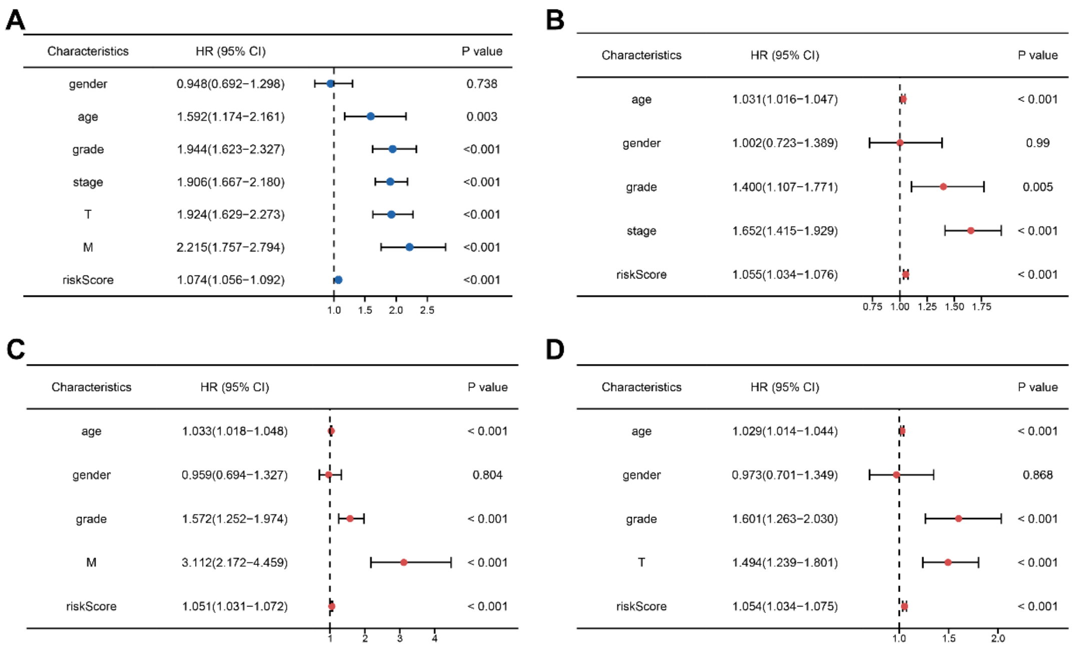


**Figure S1.** The univariate and multivariate Cox regression analysis of risk score. Notes: (**A**) Univariate Cox regression analysis of the risk score and clinical features (gender, age, grade, stage, T and M classifications). (**B**) Multivariate Cox regression analysis of the risk score and clinical features (gender, age, grade, stage). (**C**) Multivariate Cox regression analysis of the risk score and clinical features (gender, age, grade, M classification). (**D**) Multivariate Cox regression analysis of the risk score and clinical features (gender, age, grade, N classification).

| **Table S1. The list of costimulatory molecules** |
|---|
| CD27, CD274, CD276, CD28, CD40, CD40LG, CD70, CD80, CD86, CTLA4, EDA, EDA2R, EDAR, FAS, FASLG, HHLA2, ICOS, ICOSLG, LTA, LTB, LTBR, NGFR, PDCD1, PDCD1LG2, RELT, TMIGD2, TNF, TNFRSF10A, TNFRSF10B, TNFRSF10C, TNFRSF10D, TNFRSF11A, TNFRSF11B, TNFRSF12A, TNFRSF13B, TNFRSF13C, TNFRSF14, TNFRSF17, TNFRSF18, TNFRSF19, TNFRSF1A, TNFRSF1B, TNFRSF21, TNFRSF25, TNFRSF4, TNFRSF8, TNFRSF9, TNFSF10, TNFSF11, TNFSF12, TNFSF13, TNFSF13B, TNFSF14, TNFSF15, TNFSF18, TNFSF4, TNFSF8, TNFSF9, VTCN1 |

**Table S2.** The identified 1736 costimulatory molecule-related lncRNAs.

| costimulatory molecule | lncRNA | cor | pvalue | Regulation |
|---|---|---|---|---|
| TNFRSF1A | NNT-AS1 | -0.53591 | 2.13E-41 | negative |
| TNFRSF1B | LINC00426 | 0.500087 | 1.89E-35 | postive |
| RELT | AC010247.1 | 0.5001 | 1.88E-35 | postive |
| TNFRSF9 | PSMB8-AS1 | 0.500178 | 1.83E-35 | postive |
| TMIGD2 | AC010319.4 | 0.500185 | 1.82E-35 | postive |
| TNFRSF14 | AC040977.1 | 0.500264 | 1.77E-35 | postive |
| TMIGD2 | AP001527.2 | 0.500278 | 1.76E-35 | postive |
| TNFRSF25 | AC005332.4 | 0.500397 | 1.69E-35 | postive |
| TNFRSF25 | C1RL-AS1 | 0.50051 | 1.62E-35 | postive |
| TMIGD2 | AC127070.2 | 0.500513 | 1.62E-35 | postive |
| RELT | AC022182.2 | 0.500652 | 1.54E-35 | postive |
| TNFRSF18 | AC008105.3 | 0.500652 | 1.54E-35 | postive |
| TNFSF14 | ITGB2-AS1 | 0.500749 | 1.49E-35 | postive |
| TNFRSF25 | AC092171.4 | 0.500767 | 1.48E-35 | postive |

| | | | | |
|---|---|---|---|---|
| CD86 | AC243829.4 | 0.501009 | 1.35E-35 | postive |
| TMIGD2 | Z86062.2 | 0.501022 | 1.35E-35 | postive |
| CTLA4 | AC007278.1 | 0.501141 | 1.29E-35 | postive |
| PDCD1 | AC021028.1 | 0.501146 | 1.29E-35 | postive |
| TNFRSF25 | AP000254.1 | 0.501297 | 1.22E-35 | postive |
| TNFRSF25 | AC087239.1 | 0.501378 | 1.18E-35 | postive |
| TNFSF8 | TSPOAP1-AS1 | 0.501382 | 1.18E-35 | postive |
| PDCD1 | LINC00881 | 0.501668 | 1.07E-35 | postive |
| TNFRSF14 | AC008735.2 | 0.501773 | 1.03E-35 | postive |
| TNFRSF9 | AL132642.1 | 0.501785 | 1.02E-35 | postive |
| FASLG | ITGB2-AS1 | 0.502002 | 9.45E-36 | postive |
| CD80 | LINC00943 | 0.502109 | 9.09E-36 | postive |
| TNFRSF25 | LINC01138 | 0.502116 | 9.07E-36 | postive |
| TNFRSF25 | AL683813.1 | 0.502127 | 9.03E-36 | postive |
| TMIGD2 | AL512770.1 | 0.502208 | 8.77E-36 | postive |
| RELT | LINC02422 | 0.502215 | 8.75E-36 | postive |
| TNFSF14 | AC127502.2 | 0.502222 | 8.72E-36 | postive |
| TMIGD2 | AC090844.2 | 0.502237 | 8.68E-36 | postive |
| PDCD1 | AL139352.1 | 0.502259 | 8.61E-36 | postive |
| ICOS | LINC02328 | 0.502299 | 8.48E-36 | postive |
| TNFRSF14 | AC007406.2 | 0.502385 | 8.22E-36 | postive |
| CD80 | AL139125.1 | 0.502436 | 8.07E-36 | postive |
| TNFRSF25 | AC013356.2 | 0.502857 | 6.93E-36 | postive |
| TNFRSF25 | AC112722.1 | 0.503187 | 6.14E-36 | postive |
| TNFRSF25 | AC132872.3 | 0.503236 | 6.04E-36 | postive |
| LTA | LINC02273 | 0.503325 | 5.84E-36 | postive |
| TMIGD2 | AL133553.1 | 0.503578 | 5.33E-36 | postive |
| CD80 | AC006033.2 | 0.50362 | 5.25E-36 | postive |
| FAS | TNFRSF10A-AS1 | 0.503636 | 5.22E-36 | postive |
| TNFRSF25 | AL442128.2 | 0.503666 | 5.16E-36 | postive |
| TNFRSF14 | AL590822.1 | 0.504021 | 4.53E-36 | postive |
| TNFRSF25 | AL356356.1 | 0.504081 | 4.44E-36 | postive |
| TNFRSF25 | AP000692.1 | 0.504096 | 4.41E-36 | postive |
| TNFRSF25 | AC025171.4 | 0.504251 | 4.17E-36 | postive |
| EDA | AL162171.1 | 0.504357 | 4.01E-36 | postive |
| LTA | AC006033.2 | 0.504555 | 3.73E-36 | postive |
| TNFRSF14 | AC129510.1 | 0.504787 | 3.43E-36 | postive |
| CD80 | C5orf56 | 0.504829 | 3.38E-36 | postive |
| TNFRSF25 | MIR194-2HG | 0.504902 | 3.29E-36 | postive |
| TNFRSF25 | AL354892.3 | 0.504936 | 3.25E-36 | postive |
| TNFRSF14 | PSMB8-AS1 | 0.50512 | 3.04E-36 | postive |
| VTCN1 | LINC02086 | 0.505232 | 2.91E-36 | postive |
| TNFRSF25 | AC136604.2 | 0.505316 | 2.82E-36 | postive |
| TMIGD2 | AC234778.2 | 0.505326 | 2.81E-36 | postive |
| ICOS | FO393401.1 | 0.505345 | 2.79E-36 | postive |
| RELT | AL135818.2 | 0.505411 | 2.73E-36 | postive |
| TNFRSF25 | AP001107.8 | 0.505496 | 2.64E-36 | postive |
| TNFRSF25 | NFYC-AS1 | 0.505672 | 2.48E-36 | postive |
| TNFRSF9 | LINC02416 | 0.505687 | 2.47E-36 | postive |
| TNFRSF25 | AC105020.1 | 0.505689 | 2.46E-36 | postive |
| TMIGD2 | AC005089.1 | 0.505743 | 2.41E-36 | postive |
| TNFRSF25 | AL450384.2 | 0.505815 | 2.35E-36 | postive |
| TNFRSF18 | LINC02362 | 0.50586 | 2.31E-36 | postive |
| TMIGD2 | AL354928.1 | 0.506189 | 2.05E-36 | postive |
| TNFRSF9 | AC079015.1 | 0.506254 | 2.00E-36 | postive |
| ICOS | AC243829.1 | 0.506268 | 1.99E-36 | postive |
| TNFRSF8 | AC099066.2 | 0.506461 | 1.86E-36 | postive |

| | | | | |
|---|---|---|---|---|
| CD80 | AC022211.1 | 0.506599 | 1.76E-36 | postive |
| FASLG | LINC02422 | 0.506642 | 1.74E-36 | postive |
| TNFSF13B | LINC02285 | 0.50665 | 1.73E-36 | postive |
| TNFRSF25 | AC008610.1 | 0.506658 | 1.73E-36 | postive |
| TMIGD2 | AC008759.2 | 0.506795 | 1.64E-36 | postive |
| TNFRSF14 | NFYC-AS1 | 0.506872 | 1.59E-36 | postive |
| LTA | TNRC6C-AS1 | 0.506986 | 1.53E-36 | postive |
| CD80 | AC243960.1 | 0.507042 | 1.50E-36 | postive |
| TNFRSF14 | AC087500.2 | 0.507045 | 1.50E-36 | postive |
| TNFRSF14 | AC092118.2 | 0.507113 | 1.46E-36 | postive |
| TNFRSF14 | LENG8-AS1 | 0.507119 | 1.46E-36 | postive |
| CD28 | TSPOAP1-AS1 | 0.507146 | 1.44E-36 | postive |
| CD28 | AC006033.2 | 0.507203 | 1.41E-36 | postive |
| TNFRSF14 | AC012409.3 | 0.507404 | 1.31E-36 | postive |
| TNFRSF25 | AL662844.4 | 0.507407 | 1.31E-36 | postive |
| CD40LG | AC243960.1 | 0.507492 | 1.27E-36 | postive |
| TNFSF14 | AC090164.2 | 0.507517 | 1.26E-36 | postive |
| LTB | AL135818.2 | 0.507743 | 1.16E-36 | postive |
| TNFRSF25 | AP003096.1 | 0.507759 | 1.15E-36 | postive |
| TNFRSF25 | AC107081.2 | 0.507761 | 1.15E-36 | postive |
| PDCD1 | LINC02363 | 0.507774 | 1.14E-36 | postive |
| TNFRSF14 | PXN-AS1 | 0.507894 | 1.09E-36 | postive |
| TNFRSF25 | SNHG10 | 0.507917 | 1.08E-36 | postive |
| RELT | AC004585.1 | 0.508074 | 1.02E-36 | postive |
| TNFRSF14 | AC118754.1 | 0.508737 | 8.01E-37 | postive |
| TNFRSF9 | AF127936.1 | 0.509011 | 7.23E-37 | postive |
| TNFRSF14 | AC132872.2 | 0.509163 | 6.84E-37 | postive |
| TNFRSF14 | AL355353.1 | 0.509191 | 6.77E-37 | postive |
| RELT | AL139352.1 | 0.509217 | 6.70E-37 | postive |
| CD80 | AC116366.1 | 0.509355 | 6.37E-37 | postive |
| TMIGD2 | AC007613.1 | 0.509372 | 6.33E-37 | postive |
| TNFRSF14 | AL360181.2 | 0.509409 | 6.24E-37 | postive |
| LTA | AC087318.1 | 0.5095 | 6.03E-37 | postive |
| PDCD1LG2 | AL596094.1 | 0.509618 | 5.78E-37 | postive |
| TNFRSF25 | LIX1L-AS1 | 0.509623 | 5.77E-37 | postive |
| TNFSF14 | AC145423.2 | 0.509652 | 5.70E-37 | postive |
| TNFSF4 | AC091057.1 | 0.50969 | 5.62E-37 | postive |
| TNFRSF11A | LINC00900 | 0.509716 | 5.57E-37 | postive |
| TNFRSF25 | AC091185.1 | 0.50973 | 5.54E-37 | postive |
| LTB | LINC00426 | 0.509796 | 5.40E-37 | postive |
| TNFRSF13C | AL365361.1 | 0.510016 | 4.98E-37 | postive |
| FASLG | AL365361.1 | 0.510168 | 4.71E-37 | postive |
| TNFRSF14 | DM1-AS | 0.510176 | 4.69E-37 | postive |
| CD80 | LINC00861 | 0.510287 | 4.50E-37 | postive |
| TNFRSF14 | C9orf139 | 0.510332 | 4.43E-37 | postive |
| TNFRSF25 | AC136475.2 | 0.510426 | 4.28E-37 | postive |
| CD80 | AC090948.2 | 0.51051 | 4.14E-37 | postive |
| CTLA4 | AC091057.1 | 0.510549 | 4.08E-37 | postive |
| TMIGD2 | ZNF114-AS1 | 0.510588 | 4.02E-37 | postive |
| CD40LG | LINC00861 | 0.51059 | 4.02E-37 | postive |
| TNFRSF25 | LINC02145 | 0.510657 | 3.92E-37 | postive |
| TMIGD2 | AC074011.1 | 0.510714 | 3.84E-37 | postive |
| CD28 | AC243960.1 | 0.510756 | 3.78E-37 | postive |
| TNFRSF9 | AC006369.1 | 0.510917 | 3.56E-37 | postive |
| TMIGD2 | AC107081.2 | 0.510993 | 3.46E-37 | postive |
| PDCD1 | AL135818.2 | 0.511034 | 3.41E-37 | postive |
| LTB | AC011899.3 | 0.511036 | 3.41E-37 | postive |

| | | | | |
|---|---|---|---|---|
| CD80 | AC073046.1 | 0.51118 | 3.23E-37 | postive |
| TMIGD2 | AL139260.1 | 0.511186 | 3.22E-37 | postive |
| RELT | AC091057.1 | 0.511261 | 3.13E-37 | postive |
| CD80 | AC116158.1 | 0.511279 | 3.11E-37 | postive |
| TNFSF15 | AC073283.2 | 0.511289 | 3.10E-37 | postive |
| TNFRSF17 | AC022182.2 | 0.51132 | 3.06E-37 | postive |
| TNFSF4 | MIR155HG | 0.511662 | 2.70E-37 | postive |
| TNFRSF14 | AC069281.2 | 0.511711 | 2.65E-37 | postive |
| CD80 | IFNG-AS1 | 0.511763 | 2.59E-37 | postive |
| CD86 | USP30-AS1 | 0.511895 | 2.47E-37 | postive |
| TNFRSF14 | AC132192.2 | 0.511905 | 2.46E-37 | postive |
| TNFRSF25 | ZFHX2-AS1 | 0.51191 | 2.46E-37 | postive |
| CD80 | LINC01146 | 0.512067 | 2.32E-37 | postive |
| LTA | AC008105.3 | 0.512099 | 2.29E-37 | postive |
| TNFRSF14 | AC125494.2 | 0.512128 | 2.26E-37 | postive |
| CD80 | AC002091.2 | 0.512176 | 2.22E-37 | postive |
| TNFRSF25 | AC068620.2 | 0.512179 | 2.22E-37 | postive |
| VTCN1 | AC024022.1 | 0.512306 | 2.12E-37 | postive |
| TNFRSF25 | LINC02062 | 0.512311 | 2.11E-37 | postive |
| RELT | AC008750.1 | 0.512323 | 2.10E-37 | postive |
| TNFRSF25 | LINC01011 | 0.51242 | 2.03E-37 | postive |
| RELT | ITGB2-AS1 | 0.512421 | 2.03E-37 | postive |
| RELT | AC009133.3 | 0.512435 | 2.02E-37 | postive |
| TNFRSF9 | AL390729.1 | 0.512599 | 1.90E-37 | postive |
| TNFRSF14 | AC093726.2 | 0.513047 | 1.60E-37 | postive |
| CD80 | AL365361.1 | 0.513047 | 1.60E-37 | postive |
| TNFRSF25 | AC018809.1 | 0.513062 | 1.59E-37 | postive |
| TNFRSF25 | VPS9D1-AS1 | 0.513116 | 1.56E-37 | postive |
| TNFRSF25 | AC017104.1 | 0.513259 | 1.48E-37 | postive |
| TNFRSF14 | AL161452.1 | 0.513274 | 1.47E-37 | postive |
| LTB | PSMB8-AS1 | 0.513351 | 1.43E-37 | postive |
| CD80 | AC010186.3 | 0.513388 | 1.41E-37 | postive |
| TMIGD2 | HLX-AS1 | 0.513417 | 1.40E-37 | postive |
| CTLA4 | AC004494.1 | 0.513526 | 1.34E-37 | postive |
| CD28 | AF127936.1 | 0.51356 | 1.32E-37 | postive |
| RELT | TRG-AS1 | 0.513572 | 1.32E-37 | postive |
| TNFRSF25 | AL133215.1 | 0.513677 | 1.27E-37 | postive |
| TNFRSF14 | AL021707.6 | 0.513961 | 1.14E-37 | postive |
| CD80 | AL357060.1 | 0.513968 | 1.13E-37 | postive |
| RELT | AC011899.2 | 0.513971 | 1.13E-37 | postive |
| CD80 | LINC02328 | 0.513981 | 1.13E-37 | postive |
| TNFRSF25 | AC005740.4 | 0.514003 | 1.12E-37 | postive |
| TNFSF4 | AC004585.1 | 0.514117 | 1.07E-37 | postive |
| TNFRSF25 | AC093726.2 | 0.514314 | 9.95E-38 | postive |
| TNFRSF25 | AC092301.1 | 0.514452 | 9.44E-38 | postive |
| TNFRSF25 | AC079807.1 | 0.514513 | 9.23E-38 | postive |
| CD80 | AC090559.1 | 0.514585 | 8.98E-38 | postive |
| TNFRSF25 | KMT2E-AS1 | 0.514738 | 8.48E-38 | postive |
| TNFRSF14 | LINC00342 | 0.51492 | 7.91E-38 | postive |
| TNFRSF14 | ARHGAP27P1-BPTFP1-KPNA2P3 | 0.515036 | 7.57E-38 | postive |
| TNFRSF25 | AL139089.1 | 0.515139 | 7.28E-38 | postive |
| PDCD1 | AC068196.1 | 0.515214 | 7.08E-38 | postive |
| CD40LG | AL365361.1 | 0.515239 | 7.01E-38 | postive |
| LTB | AC006369.1 | 0.515257 | 6.96E-38 | postive |
| TMIGD2 | AL592494.3 | 0.515302 | 6.85E-38 | postive |
| TNFRSF25 | AC084018.2 | 0.515361 | 6.70E-38 | postive |
| PDCD1LG2 | AF127936.1 | 0.515442 | 6.49E-38 | postive |

| | | | | |
|---|---|---|---|---|
| TNFRSF25 | AC018638.7 | 0.515479 | 6.40E-38 | postive |
| TNFRSF14 | AC079906.1 | 0.515479 | 6.40E-38 | postive |
| TNFRSF25 | CACNA1C-AS2 | 0.515607 | 6.10E-38 | postive |
| RELT | AC243960.1 | 0.515645 | 6.01E-38 | postive |
| CD28 | AC006369.1 | 0.515693 | 5.90E-38 | postive |
| TNFRSF14 | AC015726.1 | 0.515948 | 5.36E-38 | postive |
| LTA | LINC02363 | 0.516239 | 4.80E-38 | postive |
| LTA | AC011899.3 | 0.516378 | 4.55E-38 | postive |
| TMIGD2 | AC092338.1 | 0.516491 | 4.36E-38 | postive |
| TNFRSF14 | AC017083.1 | 0.516531 | 4.29E-38 | postive |
| TNFRSF25 | MATN1-AS1 | 0.516571 | 4.23E-38 | postive |
| TNFRSF25 | AC118754.1 | 0.516637 | 4.13E-38 | postive |
| CD28 | AL591468.1 | 0.51683 | 3.83E-38 | postive |
| ICOS | AL135818.2 | 0.516848 | 3.81E-38 | postive |
| TNFRSF18 | AC004865.2 | 0.517286 | 3.22E-38 | postive |
| ICOS | LINC02422 | 0.517306 | 3.20E-38 | postive |
| RELT | AL731567.1 | 0.517331 | 3.17E-38 | postive |
| PDCD1LG2 | AC106786.1 | 0.517561 | 2.90E-38 | postive |
| TNFSF8 | LINC01150 | 0.517582 | 2.88E-38 | postive |
| TNFRSF13C | AC109446.3 | 0.517645 | 2.81E-38 | postive |
| TNFRSF25 | AC007278.1 | 0.51776 | 2.69E-38 | postive |
| CTLA4 | AC010247.1 | 0.517769 | 2.68E-38 | postive |
| TNFRSF14 | AL353622.2 | 0.517865 | 2.58E-38 | postive |
| CD80 | AC022973.4 | 0.517903 | 2.55E-38 | postive |
| CD86 | TRG-AS1 | 0.517968 | 2.48E-38 | postive |
| TNFSF4 | AC007728.2 | 0.518023 | 2.43E-38 | postive |
| TNFRSF25 | AC015802.5 | 0.518029 | 2.43E-38 | postive |
| LTA | LINC00528 | 0.518172 | 2.30E-38 | postive |
| TMIGD2 | AC026803.1 | 0.518183 | 2.29E-38 | postive |
| TNFRSF25 | AC132192.2 | 0.518389 | 2.11E-38 | postive |
| TMIGD2 | AC067750.1 | 0.518449 | 2.07E-38 | postive |
| CD86 | AL133264.2 | 0.518573 | 1.97E-38 | postive |
| TNFRSF9 | AC015911.3 | 0.518694 | 1.88E-38 | postive |
| TNFRSF14 | MMP25-AS1 | 0.518727 | 1.86E-38 | postive |
| TNFRSF8 | LINC02550 | 0.51876 | 1.84E-38 | postive |
| TMIGD2 | LINC01703 | 0.518867 | 1.76E-38 | postive |
| TNFRSF14 | LINC01786 | 0.518883 | 1.75E-38 | postive |
| CD28 | AC011899.2 | 0.518896 | 1.74E-38 | postive |
| TNFRSF11A | AC016405.2 | 0.519107 | 1.61E-38 | postive |
| TMIGD2 | AL021937.1 | 0.519273 | 1.51E-38 | postive |
| RELT | AL590764.1 | 0.519332 | 1.47E-38 | postive |
| VTCN1 | AL121820.2 | 0.519521 | 1.37E-38 | postive |
| TNFRSF14 | AC135050.3 | 0.519602 | 1.33E-38 | postive |
| TMIGD2 | AC027449.1 | 0.519617 | 1.32E-38 | postive |
| TNFRSF25 | AL354733.3 | 0.519674 | 1.29E-38 | postive |
| CTLA4 | LINC00892 | 0.519782 | 1.24E-38 | postive |
| TNFRSF25 | AC009107.2 | 0.51993 | 1.17E-38 | postive |
| CD80 | AC074011.1 | 0.520101 | 1.10E-38 | postive |
| ICOS | HCP5 | 0.5204 | 9.77E-39 | postive |
| TMIGD2 | Z83851.1 | 0.52062 | 8.98E-39 | postive |
| TNFRSF14 | LINC01144 | 0.521018 | 7.70E-39 | postive |
| TMIGD2 | AC093249.2 | 0.521019 | 7.70E-39 | postive |
| TMIGD2 | AC006273.1 | 0.521071 | 7.55E-39 | postive |
| TMIGD2 | AC009226.1 | 0.521084 | 7.51E-39 | postive |
| TNFRSF1B | AL591468.1 | 0.52112 | 7.40E-39 | postive |
| TNFRSF14 | ERVK9-11 | 0.521256 | 7.02E-39 | postive |
| TNFRSF25 | AL031846.2 | 0.521282 | 6.95E-39 | postive |

| | | | | |
|---|---|---|---|---|
| TNFRSF14 | AC015660.3 | 0.521488 | 6.42E-39 | postive |
| TNFRSF4 | HSPC324 | 0.521534 | 6.31E-39 | postive |
| TNFRSF14 | AC087289.2 | 0.52155 | 6.27E-39 | postive |
| TNFSF4 | AL591468.1 | 0.521617 | 6.11E-39 | postive |
| FASLG | AC011899.2 | 0.521661 | 6.01E-39 | postive |
| TNFRSF25 | C3orf35 | 0.521719 | 5.87E-39 | postive |
| PDCD1 | AC087318.1 | 0.52175 | 5.80E-39 | postive |
| TNFRSF14 | ADORA2A-AS1 | 0.521943 | 5.39E-39 | postive |
| TNFRSF25 | AF111169.3 | 0.522102 | 5.06E-39 | postive |
| TNFRSF14 | AC124944.1 | 0.522226 | 4.83E-39 | postive |
| TMIGD2 | AL360091.2 | 0.52254 | 4.27E-39 | postive |
| TNFRSF25 | AC011498.6 | 0.522552 | 4.25E-39 | postive |
| TMIGD2 | RNF217-AS1 | 0.52262 | 4.14E-39 | postive |
| RELT | LINC00426 | 0.522805 | 3.86E-39 | postive |
| TNFRSF25 | AP006621.4 | 0.522934 | 3.67E-39 | postive |
| TNFRSF14 | LINC00174 | 0.522974 | 3.61E-39 | postive |
| TMIGD2 | AL031727.2 | 0.523129 | 3.40E-39 | postive |
| TNFSF4 | PCED1B-AS1 | 0.523186 | 3.33E-39 | postive |
| TMIGD2 | LINC02569 | 0.523266 | 3.22E-39 | postive |
| TNFRSF1B | AC018755.4 | 0.523292 | 3.19E-39 | postive |
| CD80 | AC008115.3 | 0.523329 | 3.15E-39 | postive |
| TNFRSF25 | LINC00861 | 0.523797 | 2.62E-39 | postive |
| TNFRSF25 | AC067945.3 | 0.523834 | 2.58E-39 | postive |
| ICOS | LINC01934 | 0.523849 | 2.57E-39 | postive |
| TNFRSF25 | CAHM | 0.523867 | 2.55E-39 | postive |
| TNFRSF14 | C1RL-AS1 | 0.524104 | 2.33E-39 | postive |
| RELT | TNRC6C-AS1 | 0.524374 | 2.09E-39 | postive |
| TNFRSF14 | RAD51-AS1 | 0.524377 | 2.09E-39 | postive |
| RELT | LINC00926 | 0.524452 | 2.03E-39 | postive |
| TNFRSF25 | AL162274.2 | 0.524716 | 1.83E-39 | postive |
| ICOS | AC004494.1 | 0.524721 | 1.83E-39 | postive |
| ICOS | AC011899.2 | 0.524784 | 1.78E-39 | postive |
| TNFRSF25 | AC078864.1 | 0.524861 | 1.73E-39 | postive |
| TMIGD2 | AL359878.2 | 0.524868 | 1.73E-39 | postive |
| TNFRSF14 | AC005332.5 | 0.524885 | 1.72E-39 | postive |
| FASLG | AC015911.3 | 0.524885 | 1.71E-39 | postive |
| TNFRSF13C | AC022182.2 | 0.52508 | 1.59E-39 | postive |
| CD80 | AC004585.1 | 0.525112 | 1.57E-39 | postive |
| TNFRSF25 | AC239803.3 | 0.525451 | 1.37E-39 | postive |
| TNFRSF14 | AC084125.2 | 0.525529 | 1.33E-39 | postive |
| TMIGD2 | LINC01593 | 0.525552 | 1.32E-39 | postive |
| TNFRSF25 | AC013731.1 | 0.525568 | 1.31E-39 | postive |
| TNFRSF25 | AC078909.2 | 0.525682 | 1.26E-39 | postive |
| TNFRSF14 | AL662844.4 | 0.525688 | 1.25E-39 | postive |
| TNFRSF14 | AC009283.1 | 0.525935 | 1.14E-39 | postive |
| TMIGD2 | AC107057.1 | 0.525945 | 1.13E-39 | postive |
| TMIGD2 | AL139158.2 | 0.525972 | 1.12E-39 | postive |
| FASLG | AC006033.2 | 0.526029 | 1.10E-39 | postive |
| TMIGD2 | AC067747.1 | 0.526279 | 9.93E-40 | postive |
| TNFRSF13C | FAM30A | 0.526869 | 7.87E-40 | postive |
| TNFRSF25 | GEMIN7-AS1 | 0.52702 | 7.42E-40 | postive |
| TNFRSF25 | OGFR-AS1 | 0.52708 | 7.25E-40 | postive |
| TNFRSF14 | AC004253.1 | 0.52719 | 6.94E-40 | postive |
| TNFRSF25 | OBSCN-AS1 | 0.527371 | 6.46E-40 | postive |
| CD28 | AC009133.3 | 0.527474 | 6.20E-40 | postive |
| TNFRSF25 | HOTAIRM1 | 0.527511 | 6.12E-40 | postive |
| TMIGD2 | CATIP-AS2 | 0.527569 | 5.98E-40 | postive |

| | | | | |
|---|---|---|---|---|
| CD86 | AC004921.1 | 0.527595 | 5.92E-40 | postive |
| LTA | AC090152.1 | 0.527633 | 5.83E-40 | postive |
| LTB | AC004687.1 | 0.527912 | 5.22E-40 | postive |
| TNFRSF25 | AC120053.1 | 0.527927 | 5.19E-40 | postive |
| TMIGD2 | AL162430.2 | 0.527959 | 5.13E-40 | postive |
| TNFRSF14 | AC004148.2 | 0.528051 | 4.94E-40 | postive |
| CD86 | AC098613.1 | 0.528064 | 4.92E-40 | postive |
| FASLG | AC090152.1 | 0.528243 | 4.58E-40 | postive |
| TNFRSF25 | NDUFA6-DT | 0.528246 | 4.58E-40 | postive |
| TNFRSF4 | BX255925.1 | 0.528393 | 4.32E-40 | postive |
| TNFRSF25 | AC027020.2 | 0.528824 | 3.64E-40 | postive |
| CTLA4 | LINC01943 | 0.528943 | 3.47E-40 | postive |
| CTLA4 | AP002807.1 | 0.529014 | 3.38E-40 | postive |
| PDCD1LG2 | AC099850.3 | 0.529093 | 3.27E-40 | postive |
| TMIGD2 | LINC02541 | 0.529193 | 3.14E-40 | postive |
| CTLA4 | AC069281.2 | 0.529197 | 3.14E-40 | postive |
| TNFRSF17 | AC243960.1 | 0.52932 | 2.99E-40 | postive |
| TMIGD2 | AC022211.1 | 0.529323 | 2.99E-40 | postive |
| TNFRSF14 | SNHG20 | 0.529458 | 2.83E-40 | postive |
| TMIGD2 | AC087645.2 | 0.529529 | 2.75E-40 | postive |
| CD80 | DLEU7-AS1 | 0.529587 | 2.69E-40 | postive |
| TNFRSF14 | AL161669.3 | 0.529622 | 2.65E-40 | postive |
| TNFRSF25 | LINC02019 | 0.529684 | 2.59E-40 | postive |
| TNFRSF25 | AGAP11 | 0.52983 | 2.44E-40 | postive |
| TNFRSF25 | AC012409.3 | 0.529928 | 2.35E-40 | postive |
| CD40LG | LINC01215 | 0.529933 | 2.34E-40 | postive |
| PDCD1 | LINC02422 | 0.529961 | 2.32E-40 | postive |
| TNFRSF13C | AL133467.1 | 0.530268 | 2.05E-40 | postive |
| TNFRSF14 | AC104809.2 | 0.530938 | 1.57E-40 | postive |
| TNFRSF25 | AC104758.2 | 0.531036 | 1.51E-40 | postive |
| TMIGD2 | AL121890.5 | 0.531058 | 1.50E-40 | postive |
| TNFRSF25 | AL359532.1 | 0.531461 | 1.27E-40 | postive |
| TNFRSF25 | AC104809.2 | 0.531597 | 1.21E-40 | postive |
| TNFRSF14 | HRAT92 | 0.531748 | 1.14E-40 | postive |
| TNFRSF25 | GARS-DT | 0.531767 | 1.13E-40 | postive |
| TNFRSF25 | AL161935.1 | 0.532061 | 1.00E-40 | postive |
| TNFRSF13C | AC008083.2 | 0.532161 | 9.63E-41 | postive |
| TMIGD2 | AL021707.2 | 0.532203 | 9.47E-41 | postive |
| TNFRSF14 | BCRP3 | 0.532341 | 8.96E-41 | postive |
| TMIGD2 | AL607028.1 | 0.532415 | 8.70E-41 | postive |
| TNFRSF4 | AL391845.2 | 0.532484 | 8.46E-41 | postive |
| TNFRSF14 | AL121992.3 | 0.532515 | 8.36E-41 | postive |
| TMIGD2 | AL139125.1 | 0.532776 | 7.53E-41 | postive |
| RELT | AC069281.2 | 0.532783 | 7.51E-41 | postive |
| CTLA4 | LINC02195 | 0.532849 | 7.31E-41 | postive |
| TNFSF13B | AC243960.1 | 0.532942 | 7.04E-41 | postive |
| TNFRSF25 | AC016590.1 | 0.532995 | 6.89E-41 | postive |
| TNFRSF25 | UBE2Q1-AS1 | 0.533028 | 6.80E-41 | postive |
| TNFRSF25 | AL159169.2 | 0.533078 | 6.67E-41 | postive |
| TNFRSF25 | AL161669.3 | 0.533138 | 6.51E-41 | postive |
| RELT | LINC01943 | 0.533178 | 6.41E-41 | postive |
| RELT | AC004771.1 | 0.533236 | 6.26E-41 | postive |
| TNFRSF25 | AC015961.2 | 0.533245 | 6.24E-41 | postive |
| TNFRSF25 | AL355488.1 | 0.533289 | 6.13E-41 | postive |
| TNFRSF25 | AL451050.2 | 0.53342 | 5.81E-41 | postive |
| TNFRSF25 | U47924.3 | 0.53384 | 4.91E-41 | postive |
| TMIGD2 | AC005342.2 | 0.533965 | 4.67E-41 | postive |

| | | | | |
|---|---|---|---|---|
| TMIGD2 | AC002128.2 | 0.534223 | 4.21E-41 | postive |
| TNFRSF14 | PRKCZ-AS1 | 0.534306 | 4.07E-41 | postive |
| TMIGD2 | AC104667.2 | 0.53431 | 4.06E-41 | postive |
| CD80 | AC035139.1 | 0.534441 | 3.85E-41 | postive |
| TNFRSF25 | AC009404.1 | 0.534529 | 3.72E-41 | postive |
| TNFRSF1B | AC004865.2 | 0.534591 | 3.63E-41 | postive |
| CTLA4 | LINC02328 | 0.534856 | 3.26E-41 | postive |
| TNFRSF14 | PLA2G4C-AS1 | 0.534982 | 3.10E-41 | postive |
| TNFRSF17 | AC087752.3 | 0.535106 | 2.94E-41 | postive |
| TNFRSF14 | AL022322.1 | 0.535114 | 2.93E-41 | postive |
| TNFRSF1B | AC010247.1 | 0.535127 | 2.92E-41 | postive |
| CD40LG | AC006369.1 | 0.5352 | 2.84E-41 | postive |
| TNFRSF14 | HPN-AS1 | 0.535211 | 2.82E-41 | postive |
| CD86 | AC018755.4 | 0.535351 | 2.67E-41 | postive |
| TMIGD2 | AL138921.2 | 0.535369 | 2.65E-41 | postive |
| TNFRSF25 | AC099568.1 | 0.535428 | 2.59E-41 | postive |
| TNFRSF25 | AL133410.1 | 0.53553 | 2.48E-41 | postive |
| LTA | AL365361.1 | 0.535749 | 2.27E-41 | postive |
| TNFSF4 | AL139352.1 | 0.535824 | 2.20E-41 | postive |
| TMIGD2 | AL391261.2 | 0.536105 | 1.96E-41 | postive |
| TNFRSF9 | AL139352.1 | 0.536164 | 1.92E-41 | postive |
| TNFRSF25 | AC016026.1 | 0.536387 | 1.75E-41 | postive |
| TNFRSF25 | AC087289.5 | 0.536398 | 1.74E-41 | postive |
| TMIGD2 | MIR583HG | 0.536439 | 1.72E-41 | postive |
| LTB | LINC01215 | 0.536471 | 1.69E-41 | postive |
| TMIGD2 | AC007036.2 | 0.536539 | 1.65E-41 | postive |
| TNFRSF18 | AC147067.1 | 0.536573 | 1.62E-41 | postive |
| TNFSF4 | AL135818.1 | 0.536682 | 1.55E-41 | postive |
| TMIGD2 | LINC01750 | 0.536864 | 1.44E-41 | postive |
| TNFSF8 | AL365361.1 | 0.536998 | 1.37E-41 | postive |
| CTLA4 | HLA-DQB1-AS1 | 0.537173 | 1.27E-41 | postive |
| LTB | AC012645.3 | 0.537208 | 1.25E-41 | postive |
| TNFRSF13C | LINC01215 | 0.537211 | 1.25E-41 | postive |
| CTLA4 | RRN3P2 | 0.537392 | 1.16E-41 | postive |
| TNFRSF1B | AC004585.1 | 0.537423 | 1.15E-41 | postive |
| TNFRSF25 | AC048341.2 | 0.537586 | 1.08E-41 | postive |
| CD274 | CASC19 | 0.537628 | 1.06E-41 | postive |
| TNFRSF25 | BREA2 | 0.537741 | 1.01E-41 | postive |
| FASLG | HCP5 | 0.537818 | 9.78E-42 | postive |
| TNFRSF25 | AC015726.1 | 0.537847 | 9.67E-42 | postive |
| TMIGD2 | AC008915.2 | 0.538012 | 9.04E-42 | postive |
| LTA | LINC01943 | 0.538022 | 9.00E-42 | postive |
| TNFRSF25 | AC084117.1 | 0.538047 | 8.91E-42 | postive |
| TNFRSF25 | LINC00173 | 0.538064 | 8.85E-42 | postive |
| TNFRSF8 | AC124067.2 | 0.538378 | 7.78E-42 | postive |
| TMIGD2 | AL365295.1 | 0.538455 | 7.54E-42 | postive |
| TMIGD2 | AC092718.2 | 0.538806 | 6.53E-42 | postive |
| TMIGD2 | AP000346.1 | 0.539064 | 5.88E-42 | postive |
| TNFRSF14 | AL359504.2 | 0.539249 | 5.45E-42 | postive |
| TMIGD2 | AC021188.1 | 0.539259 | 5.43E-42 | postive |
| TNFRSF25 | IGBP1-AS1 | 0.539514 | 4.89E-42 | postive |
| ICOS | AC012645.3 | 0.539701 | 4.52E-42 | postive |
| TNFRSF13C | AC104971.3 | 0.539982 | 4.03E-42 | postive |
| TNFSF13B | USP30-AS1 | 0.540028 | 3.96E-42 | postive |
| TMIGD2 | AC090907.2 | 0.540093 | 3.85E-42 | postive |
| TMIGD2 | RC3H1-IT1 | 0.540133 | 3.79E-42 | postive |
| TNFRSF4 | SENCR | 0.54032 | 3.51E-42 | postive |

| | | | | |
|---|---|---|---|---|
| TNFRSF25 | AC008105.1 | 0.540356 | 3.46E-42 | postive |
| FASLG | AC010247.1 | 0.54038 | 3.42E-42 | postive |
| TNFRSF14 | Z69706.1 | 0.540422 | 3.36E-42 | postive |
| TNFRSF25 | INE1 | 0.54046 | 3.31E-42 | postive |
| TNFSF13B | AC011899.3 | 0.540557 | 3.18E-42 | postive |
| TNFRSF25 | AL008582.1 | 0.540625 | 3.09E-42 | postive |
| TMIGD2 | AC023355.1 | 0.540817 | 2.86E-42 | postive |
| TNFRSF14 | AP006284.1 | 0.540834 | 2.84E-42 | postive |
| TMIGD2 | AL807757.2 | 0.540835 | 2.84E-42 | postive |
| TMIGD2 | AC015818.2 | 0.540856 | 2.81E-42 | postive |
| TNFRSF25 | AC044849.1 | 0.540957 | 2.70E-42 | postive |
| TNFRSF25 | AC090510.2 | 0.540996 | 2.66E-42 | postive |
| TNFRSF25 | AC087289.1 | 0.541175 | 2.47E-42 | postive |
| RELT | AC025857.2 | 0.541179 | 2.46E-42 | postive |
| LTB | LINC00528 | 0.541356 | 2.29E-42 | postive |
| CTLA4 | LINC00937 | 0.541372 | 2.27E-42 | postive |
| TNFSF14 | AL596223.2 | 0.541456 | 2.20E-42 | postive |
| TNFRSF25 | GATA6-AS1 | 0.541485 | 2.17E-42 | postive |
| TNFRSF25 | AL137784.2 | 0.541571 | 2.09E-42 | postive |
| TNFRSF25 | AC010326.3 | 0.541829 | 1.88E-42 | postive |
| TNFRSF25 | AC023908.3 | 0.541849 | 1.87E-42 | postive |
| TMIGD2 | AC145423.3 | 0.541981 | 1.77E-42 | postive |
| CD80 | BHLHE40-AS1 | 0.54219 | 1.62E-42 | postive |
| TNFRSF25 | MCCC1-AS1 | 0.542313 | 1.54E-42 | postive |
| FASLG | AC243829.1 | 0.542375 | 1.50E-42 | postive |
| CD28 | AL590764.1 | 0.542436 | 1.46E-42 | postive |
| CTLA4 | AL158071.3 | 0.542514 | 1.42E-42 | postive |
| TNFRSF25 | LINC00685 | 0.542576 | 1.38E-42 | postive |
| TNFRSF25 | AP4B1-AS1 | 0.542616 | 1.36E-42 | postive |
| TNFRSF25 | ATP2C2-AS1 | 0.54301 | 1.15E-42 | postive |
| TNFSF4 | LINC00158 | 0.543012 | 1.15E-42 | postive |
| LTB | TRG-AS1 | 0.543102 | 1.11E-42 | postive |
| TNFSF8 | TRG-AS1 | 0.54315 | 1.09E-42 | postive |
| TNFRSF25 | AL512791.1 | 0.543199 | 1.07E-42 | postive |
| CD80 | AC025857.2 | 0.543207 | 1.06E-42 | postive |
| TMIGD2 | AP001160.4 | 0.543293 | 1.03E-42 | postive |
| TMIGD2 | AC008731.1 | 0.543302 | 1.02E-42 | postive |
| TMIGD2 | AC124303.1 | 0.54331 | 1.02E-42 | postive |
| CD40LG | PCED1B-AS1 | 0.543536 | 9.27E-43 | postive |
| LTB | AL135818.1 | 0.543739 | 8.52E-43 | postive |
| TNFRSF25 | GRPEL2-AS1 | 0.543879 | 8.04E-43 | postive |
| CTLA4 | AC008750.1 | 0.543907 | 7.94E-43 | postive |
| TMIGD2 | AC048382.1 | 0.54405 | 7.48E-43 | postive |
| TNFRSF25 | AC092809.4 | 0.544278 | 6.80E-43 | postive |
| TNFRSF25 | AL731571.1 | 0.544573 | 6.02E-43 | postive |
| TNFRSF14 | AL121845.4 | 0.544601 | 5.95E-43 | postive |
| TNFSF4 | AC243829.4 | 0.544712 | 5.68E-43 | postive |
| TNFRSF18 | LINC01871 | 0.544957 | 5.12E-43 | postive |
| TMIGD2 | AC010149.1 | 0.54499 | 5.05E-43 | postive |
| TNFRSF25 | AL031282.2 | 0.545054 | 4.92E-43 | postive |
| CD80 | PCED1B-AS1 | 0.545147 | 4.73E-43 | postive |
| TNFRSF25 | AC139530.1 | 0.545147 | 4.73E-43 | postive |
| TMIGD2 | LIX1L-AS1 | 0.545176 | 4.68E-43 | postive |
| TNFRSF25 | MIR210HG | 0.545301 | 4.44E-43 | postive |
| TMIGD2 | AL118511.1 | 0.545403 | 4.25E-43 | postive |
| RELT | AC004865.2 | 0.545584 | 3.94E-43 | postive |
| FASLG | AL135818.2 | 0.545612 | 3.90E-43 | postive |

| | | | | |
|---|---|---|---|---|
| TNFRSF25 | AP001458.1 | 0.545654 | 3.83E-43 | postive |
| TMIGD2 | AC012360.2 | 0.545677 | 3.79E-43 | postive |
| TNFRSF14 | AC004918.1 | 0.545696 | 3.76E-43 | postive |
| LTB | LINC02422 | 0.545749 | 3.68E-43 | postive |
| TNFRSF25 | Z84485.1 | 0.545847 | 3.53E-43 | postive |
| TNFRSF18 | AC012236.1 | 0.545983 | 3.33E-43 | postive |
| TNFRSF14 | AL136304.1 | 0.546091 | 3.19E-43 | postive |
| TMIGD2 | DAPK1-IT1 | 0.546219 | 3.02E-43 | postive |
| TNFRSF1B | TRG-AS1 | 0.546377 | 2.83E-43 | postive |
| TNFRSF25 | AC092119.2 | 0.546388 | 2.81E-43 | postive |
| TNFSF8 | PCED1B-AS1 | 0.546816 | 2.35E-43 | postive |
| LTB | LINC02084 | 0.546833 | 2.33E-43 | postive |
| TMIGD2 | LINC02084 | 0.54728 | 1.93E-43 | postive |
| TNFRSF14 | ZNF213-AS1 | 0.547539 | 1.73E-43 | postive |
| TNFRSF9 | MIR155HG | 0.5476 | 1.69E-43 | postive |
| TNFRSF14 | AC024337.2 | 0.547667 | 1.64E-43 | postive |
| TNFRSF25 | AC087500.1 | 0.547924 | 1.47E-43 | postive |
| TNFRSF4 | AL355803.1 | 0.54829 | 1.26E-43 | postive |
| TNFRSF25 | AC087481.3 | 0.548361 | 1.22E-43 | postive |
| TMIGD2 | AP001010.1 | 0.548668 | 1.08E-43 | postive |
| TNFRSF25 | AC073611.1 | 0.548731 | 1.05E-43 | postive |
| TNFRSF25 | AC010245.2 | 0.548811 | 1.01E-43 | postive |
| FASLG | AL132642.1 | 0.549009 | 9.31E-44 | postive |
| TMIGD2 | AC087501.4 | 0.549092 | 8.99E-44 | postive |
| TNFRSF25 | AC024060.1 | 0.549341 | 8.09E-44 | postive |
| TMIGD2 | AC006270.1 | 0.549384 | 7.94E-44 | postive |
| LTB | AL391069.3 | 0.549491 | 7.59E-44 | postive |
| TMIGD2 | AC073592.1 | 0.549499 | 7.56E-44 | postive |
| ICOS | PSMB8-AS1 | 0.549501 | 7.56E-44 | postive |
| LTB | AC022182.2 | 0.549567 | 7.35E-44 | postive |
| TNFRSF14 | AC078864.1 | 0.549602 | 7.24E-44 | postive |
| TNFRSF17 | LINC02422 | 0.549809 | 6.63E-44 | postive |
| TNFRSF14 | AC012615.6 | 0.549851 | 6.51E-44 | postive |
| TMIGD2 | AL161909.2 | 0.549896 | 6.39E-44 | postive |
| TNFRSF8 | AC099786.1 | 0.549916 | 6.34E-44 | postive |
| TNFRSF25 | AC007566.1 | 0.549932 | 6.29E-44 | postive |
| TNFRSF25 | AC127521.1 | 0.549953 | 6.24E-44 | postive |
| TNFRSF25 | AP000892.2 | 0.549987 | 6.15E-44 | postive |
| TMIGD2 | AP001029.1 | 0.550096 | 5.87E-44 | postive |
| TNFRSF25 | AL109741.1 | 0.550141 | 5.76E-44 | postive |
| TMIGD2 | AC100861.1 | 0.550163 | 5.70E-44 | postive |
| CD80 | LINC01934 | 0.550221 | 5.56E-44 | postive |
| TNFRSF25 | AL022238.3 | 0.550294 | 5.39E-44 | postive |
| TNFRSF25 | AL138921.1 | 0.550392 | 5.17E-44 | postive |
| PDCD1 | PSMB8-AS1 | 0.550392 | 5.17E-44 | postive |
| TNFRSF13C | AC243960.1 | 0.550397 | 5.16E-44 | postive |
| TMIGD2 | AL031848.1 | 0.550408 | 5.14E-44 | postive |
| CD28 | AC007728.2 | 0.550568 | 4.80E-44 | postive |
| TNFRSF25 | AC104463.2 | 0.550887 | 4.19E-44 | postive |
| TMIGD2 | LINC00299 | 0.550919 | 4.13E-44 | postive |
| TNFRSF25 | AL121852.1 | 0.550983 | 4.02E-44 | postive |
| TMIGD2 | AL133243.1 | 0.551035 | 3.93E-44 | postive |
| RELT | LINC00528 | 0.551119 | 3.79E-44 | postive |
| TNFRSF25 | AC104964.3 | 0.551125 | 3.78E-44 | postive |
| TNFRSF25 | AC012645.3 | 0.551152 | 3.74E-44 | postive |
| TMIGD2 | AC078962.1 | 0.551568 | 3.13E-44 | postive |
| TNFRSF25 | Z97832.2 | 0.55157 | 3.13E-44 | postive |

| | | | | |
|---|---|---|---|---|
| ICOS | AL139352.1 | 0.551633 | 3.05E-44 | postive |
| TNFRSF25 | MIR3936HG | 0.551819 | 2.81E-44 | postive |
| TNFRSF25 | AL592211.1 | 0.551921 | 2.69E-44 | postive |
| TNFRSF25 | AL021707.1 | 0.552055 | 2.54E-44 | postive |
| TMIGD2 | PGM5P4-AS1 | 0.552101 | 2.49E-44 | postive |
| LTA | ITGB2-AS1 | 0.552189 | 2.40E-44 | postive |
| CTLA4 | LINC02363 | 0.552223 | 2.37E-44 | postive |
| TNFSF8 | AC243829.4 | 0.552381 | 2.21E-44 | postive |
| TNFRSF25 | AL133406.2 | 0.552387 | 2.21E-44 | postive |
| TNFSF13B | AL365361.1 | 0.552468 | 2.13E-44 | postive |
| TNFRSF25 | AL354760.1 | 0.552543 | 2.06E-44 | postive |
| TNFRSF9 | HCP5 | 0.552557 | 2.05E-44 | postive |
| CTLA4 | AL365361.1 | 0.552649 | 1.97E-44 | postive |
| TNFRSF4 | AL359853.1 | 0.55273 | 1.90E-44 | postive |
| TNFRSF25 | AC024337.2 | 0.552739 | 1.90E-44 | postive |
| RELT | AP002807.1 | 0.552758 | 1.88E-44 | postive |
| TNFSF13B | AC004585.1 | 0.552922 | 1.75E-44 | postive |
| TMIGD2 | AC084357.2 | 0.553136 | 1.60E-44 | postive |
| CD80 | RRN3P2 | 0.553141 | 1.60E-44 | postive |
| LTB | LINC00892 | 0.553184 | 1.57E-44 | postive |
| TNFRSF14 | MHENCR | 0.553241 | 1.53E-44 | postive |
| TNFRSF13C | LINC02422 | 0.553482 | 1.38E-44 | postive |
| TMIGD2 | AC009630.1 | 0.553544 | 1.34E-44 | postive |
| TMIGD2 | AL358472.3 | 0.553713 | 1.25E-44 | postive |
| TNFSF8 | AL591468.1 | 0.553846 | 1.18E-44 | postive |
| TNFRSF1B | AC007728.2 | 0.553889 | 1.16E-44 | postive |
| CD86 | AC007728.2 | 0.554031 | 1.09E-44 | postive |
| CD28 | AL135818.1 | 0.554057 | 1.08E-44 | postive |
| TMIGD2 | AC011481.2 | 0.554166 | 1.03E-44 | postive |
| LTB | ITGB2-AS1 | 0.554247 | 9.91E-45 | postive |
| TNFRSF25 | AC106782.6 | 0.554488 | 8.93E-45 | postive |
| TMIGD2 | LINC01786 | 0.554523 | 8.80E-45 | postive |
| TNFRSF25 | AL096701.3 | 0.554585 | 8.57E-45 | postive |
| TNFRSF25 | LINC01311 | 0.554655 | 8.31E-45 | postive |
| TNFRSF14 | LINC01569 | 0.555036 | 7.05E-45 | postive |
| TMIGD2 | FLVCR1-DT | 0.555089 | 6.89E-45 | postive |
| TNFRSF25 | AC020558.2 | 0.555123 | 6.79E-45 | postive |
| TMIGD2 | CDKN2B-AS1 | 0.555464 | 5.86E-45 | postive |
| TMIGD2 | AC055713.1 | 0.555754 | 5.17E-45 | postive |
| TNFRSF25 | AC017083.1 | 0.556029 | 4.59E-45 | postive |
| TNFRSF14 | AC245052.4 | 0.556088 | 4.47E-45 | postive |
| TMIGD2 | AC145343.1 | 0.556179 | 4.30E-45 | postive |
| FASLG | LINC00861 | 0.556182 | 4.29E-45 | postive |
| TNFRSF25 | AC005840.2 | 0.556399 | 3.90E-45 | postive |
| CD80 | AL133371.2 | 0.556455 | 3.81E-45 | postive |
| TNFRSF25 | KDM4A-AS1 | 0.556527 | 3.69E-45 | postive |
| CD80 | CELF2-AS1 | 0.55654 | 3.67E-45 | postive |
| LTA | AC079015.1 | 0.556544 | 3.67E-45 | postive |
| FASLG | AC006369.1 | 0.55671 | 3.41E-45 | postive |
| TNFRSF25 | AC018653.3 | 0.556776 | 3.31E-45 | postive |
| TMIGD2 | AC078883.2 | 0.55684 | 3.22E-45 | postive |
| TNFRSF25 | AC034236.2 | 0.556913 | 3.12E-45 | postive |
| TMIGD2 | AC091212.1 | 0.556955 | 3.07E-45 | postive |
| LTA | LINC00861 | 0.556966 | 3.05E-45 | postive |
| TNFRSF25 | AP001453.1 | 0.557213 | 2.74E-45 | postive |
| TNFRSF25 | AC244197.2 | 0.557257 | 2.69E-45 | postive |
| TMIGD2 | AL136295.2 | 0.557294 | 2.64E-45 | postive |

| | | | | |
|---|---|---|---|---|
| TMIGD2 | AL078587.1 | 0.557429 | 2.49E-45 | postive |
| TMIGD2 | AC011978.2 | 0.557549 | 2.37E-45 | postive |
| TNFRSF25 | AC004832.6 | 0.557594 | 2.32E-45 | postive |
| TNFRSF25 | UTAT33 | 0.557596 | 2.32E-45 | postive |
| TMIGD2 | PRR7-AS1 | 0.557604 | 2.31E-45 | postive |
| TMIGD2 | AC084809.1 | 0.557855 | 2.07E-45 | postive |
| TNFRSF13C | IFNG-AS1 | 0.557953 | 1.98E-45 | postive |
| TNFRSF25 | AL671710.1 | 0.557989 | 1.95E-45 | postive |
| TNFRSF25 | AP006621.2 | 0.558044 | 1.91E-45 | postive |
| TMIGD2 | AC011479.2 | 0.558047 | 1.90E-45 | postive |
| TNFRSF25 | AP002490.1 | 0.558534 | 1.54E-45 | postive |
| LTA | LINC02422 | 0.558714 | 1.42E-45 | postive |
| CTLA4 | AC004687.1 | 0.558811 | 1.36E-45 | postive |
| TMIGD2 | AC005759.1 | 0.558817 | 1.36E-45 | postive |
| TNFRSF25 | PXN-AS1 | 0.55882 | 1.36E-45 | postive |
| TNFRSF25 | AC073842.2 | 0.558867 | 1.33E-45 | postive |
| TMIGD2 | WWTR1-IT1 | 0.558889 | 1.32E-45 | postive |
| TNFSF4 | TRG-AS1 | 0.558928 | 1.30E-45 | postive |
| CD80 | AC138207.5 | 0.559096 | 1.20E-45 | postive |
| TNFRSF25 | AC010542.5 | 0.559108 | 1.20E-45 | postive |
| TMIGD2 | SLC16A1-AS1 | 0.559325 | 1.09E-45 | postive |
| VTCN1 | AC004846.1 | 0.559408 | 1.05E-45 | postive |
| TNFRSF25 | AC010761.1 | 0.55954 | 9.90E-46 | postive |
| TNFRSF14 | U62317.2 | 0.559643 | 9.46E-46 | postive |
| CD86 | AL133371.2 | 0.559884 | 8.51E-46 | postive |
| TNFRSF25 | PHKA2-AS1 | 0.560036 | 7.96E-46 | postive |
| TNFRSF25 | AC005899.6 | 0.560069 | 7.85E-46 | postive |
| CTLA4 | FO393401.1 | 0.560111 | 7.70E-46 | postive |
| TNFSF8 | AL590764.1 | 0.560177 | 7.48E-46 | postive |
| TNFRSF25 | Z99916.1 | 0.560217 | 7.35E-46 | postive |
| TNFRSF25 | AC127024.6 | 0.560359 | 6.90E-46 | postive |
| LTB | AC147067.1 | 0.560474 | 6.56E-46 | postive |
| TNFRSF25 | AL031709.1 | 0.560793 | 5.70E-46 | postive |
| LTB | LINC01871 | 0.560829 | 5.61E-46 | postive |
| PDCD1LG2 | AC004816.2 | 0.560911 | 5.41E-46 | postive |
| LTA | AC068196.1 | 0.561003 | 5.20E-46 | postive |
| TNFRSF11A | AC010247.2 | 0.561232 | 4.70E-46 | postive |
| VTCN1 | ERVE-1 | 0.561236 | 4.69E-46 | postive |
| TNFRSF25 | AL096865.1 | 0.56125 | 4.66E-46 | postive |
| TMIGD2 | AC015689.1 | 0.561251 | 4.66E-46 | postive |
| TNFRSF25 | NALT1 | 0.561387 | 4.39E-46 | postive |
| CD86 | AC145098.1 | 0.561498 | 4.18E-46 | postive |
| TNFRSF25 | AL359921.1 | 0.561553 | 4.08E-46 | postive |
| TMIGD2 | AC106028.3 | 0.561558 | 4.07E-46 | postive |
| RELT | AC012645.3 | 0.561719 | 3.79E-46 | postive |
| TMIGD2 | AC244021.1 | 0.562238 | 3.01E-46 | postive |
| TNFRSF14 | AP000345.2 | 0.562396 | 2.81E-46 | postive |
| TMIGD2 | AL049539.1 | 0.562426 | 2.77E-46 | postive |
| VTCN1 | LINC00686 | 0.562546 | 2.63E-46 | postive |
| TNFRSF9 | AC243960.1 | 0.562608 | 2.55E-46 | postive |
| TNFRSF25 | AC145285.6 | 0.56265 | 2.51E-46 | postive |
| RELT | AC007728.2 | 0.562706 | 2.45E-46 | postive |
| TNFRSF25 | AC008735.1 | 0.56278 | 2.37E-46 | postive |
| CD80 | AL135818.1 | 0.562829 | 2.32E-46 | postive |
| TNFSF8 | LINC00426 | 0.563203 | 1.96E-46 | postive |
| TNFRSF14 | AC009974.1 | 0.563291 | 1.89E-46 | postive |
| TNFRSF25 | AC002059.1 | 0.563578 | 1.66E-46 | postive |

| | | | | |
|---|---|---|---|---|
| CD80 | AC011899.2 | 0.563932 | 1.42E-46 | postive |
| TMIGD2 | AC114980.1 | 0.563981 | 1.39E-46 | postive |
| TNFRSF25 | AC124944.1 | 0.564005 | 1.37E-46 | postive |
| TMIGD2 | AL158825.2 | 0.564009 | 1.37E-46 | postive |
| TMIGD2 | AC104463.2 | 0.564245 | 1.23E-46 | postive |
| TNFRSF25 | AP001010.1 | 0.564322 | 1.19E-46 | postive |
| TNFRSF25 | AC024075.2 | 0.564504 | 1.10E-46 | postive |
| TNFRSF25 | AL049840.4 | 0.564536 | 1.08E-46 | postive |
| LTA | LINC01871 | 0.5646 | 1.05E-46 | postive |
| TNFSF13B | AC018755.4 | 0.565144 | 8.25E-47 | postive |
| TNFRSF25 | AL158212.2 | 0.565177 | 8.13E-47 | postive |
| TMIGD2 | AP003419.3 | 0.565239 | 7.91E-47 | postive |
| TNFRSF25 | AC055713.1 | 0.565251 | 7.86E-47 | postive |
| TNFSF8 | AC007728.2 | 0.565355 | 7.50E-47 | postive |
| TNFRSF25 | AC008735.4 | 0.56557 | 6.82E-47 | postive |
| TMIGD2 | AC116348.1 | 0.565587 | 6.77E-47 | postive |
| TMIGD2 | AC026741.1 | 0.565596 | 6.74E-47 | postive |
| TNFRSF25 | CAPN10-DT | 0.565764 | 6.25E-47 | postive |
| TNFRSF25 | LINC00487 | 0.565782 | 6.20E-47 | postive |
| TNFRSF25 | AC100793.3 | 0.56597 | 5.70E-47 | postive |
| TNFRSF1B | AC090559.1 | 0.566027 | 5.55E-47 | postive |
| TNFRSF14 | AC004846.2 | 0.566072 | 5.44E-47 | postive |
| CD86 | LINC01150 | 0.566082 | 5.42E-47 | postive |
| ICOS | AC006369.1 | 0.566178 | 5.19E-47 | postive |
| TNFRSF17 | IFNG-AS1 | 0.566571 | 4.35E-47 | postive |
| TNFRSF14 | AL139287.1 | 0.566593 | 4.31E-47 | postive |
| TNFRSF14 | AC022144.1 | 0.566727 | 4.05E-47 | postive |
| PDCD1 | LINC00539 | 0.56681 | 3.91E-47 | postive |
| TNFRSF14 | AC105020.6 | 0.566825 | 3.88E-47 | postive |
| CD80 | FO393401.1 | 0.56683 | 3.87E-47 | postive |
| TNFSF13B | AC007728.2 | 0.567028 | 3.54E-47 | postive |
| TMIGD2 | LINC01376 | 0.567288 | 3.15E-47 | postive |
| TMIGD2 | LINC02487 | 0.567409 | 2.98E-47 | postive |
| TNFRSF25 | AC084876.1 | 0.567497 | 2.87E-47 | postive |
| CD80 | AC091057.1 | 0.567512 | 2.85E-47 | postive |
| TNFRSF25 | AC002553.1 | 0.567621 | 2.71E-47 | postive |
| CTLA4 | U62317.2 | 0.567663 | 2.66E-47 | postive |
| TNFRSF25 | AP003352.1 | 0.567672 | 2.65E-47 | postive |
| LTA | AL135818.2 | 0.567705 | 2.61E-47 | postive |
| PDCD1 | AC012645.3 | 0.567776 | 2.53E-47 | postive |
| TMIGD2 | AC114956.2 | 0.567811 | 2.49E-47 | postive |
| TMIGD2 | AL731569.1 | 0.567875 | 2.42E-47 | postive |
| TNFRSF25 | AC006064.3 | 0.567976 | 2.31E-47 | postive |
| FASLG | AC087318.1 | 0.567999 | 2.28E-47 | postive |
| LTB | LINC01943 | 0.568033 | 2.25E-47 | postive |
| CD80 | AC007000.3 | 0.568174 | 2.11E-47 | postive |
| TNFRSF25 | Z82188.2 | 0.568229 | 2.06E-47 | postive |
| TNFRSF8 | LINC01686 | 0.568236 | 2.05E-47 | postive |
| TMIGD2 | AC024941.2 | 0.568273 | 2.02E-47 | postive |
| TMIGD2 | AC137932.3 | 0.56836 | 1.94E-47 | postive |
| LTA | MIR155HG | 0.568475 | 1.84E-47 | postive |
| PDCD1LG2 | AC004825.2 | 0.568575 | 1.76E-47 | postive |
| TMIGD2 | AC127024.2 | 0.568642 | 1.71E-47 | postive |
| TNFRSF25 | AC004951.4 | 0.568682 | 1.68E-47 | postive |
| TNFRSF1B | AC011899.3 | 0.569135 | 1.37E-47 | postive |
| TNFRSF14 | STAG3L5P-PVRIG2P-PILRB | 0.569146 | 1.36E-47 | postive |
| TNFRSF25 | AL008729.2 | 0.569199 | 1.33E-47 | postive |

| | | | | |
|---|---|---|---|---|
| TNFRSF14 | AL353622.1 | 0.569313 | 1.26E-47 | postive |
| TMIGD2 | SUCLA2-AS1 | 0.569453 | 1.18E-47 | postive |
| TMIGD2 | AC004921.1 | 0.569454 | 1.18E-47 | postive |
| TNFSF4 | AC022126.1 | 0.569492 | 1.16E-47 | postive |
| TNFRSF25 | AC007292.1 | 0.569589 | 1.11E-47 | postive |
| LTA | AC011899.2 | 0.569696 | 1.06E-47 | postive |
| TNFRSF25 | AC084824.5 | 0.569765 | 1.03E-47 | postive |
| CD80 | LINC00158 | 0.569803 | 1.01E-47 | postive |
| TMIGD2 | WNT5A-AS1 | 0.569897 | 9.66E-48 | postive |
| TNFRSF25 | AL121987.2 | 0.57001 | 9.18E-48 | postive |
| LTA | LINC00539 | 0.570371 | 7.78E-48 | postive |
| CD40LG | LINC02273 | 0.570467 | 7.45E-48 | postive |
| TNFRSF25 | AC012360.3 | 0.570746 | 6.56E-48 | postive |
| TNFSF4 | LINC00426 | 0.571083 | 5.62E-48 | postive |
| TMIGD2 | AC009950.1 | 0.571392 | 4.88E-48 | postive |
| TNFRSF25 | HCG27 | 0.571421 | 4.82E-48 | postive |
| CD80 | AC243829.4 | 0.571532 | 4.58E-48 | postive |
| CD28 | AC243829.4 | 0.571882 | 3.90E-48 | postive |
| TNFRSF25 | AC004034.1 | 0.572183 | 3.40E-48 | postive |
| TNFRSF25 | HPN-AS1 | 0.572275 | 3.26E-48 | postive |
| TMIGD2 | AC008514.1 | 0.572432 | 3.03E-48 | postive |
| PDCD1 | TSPOAP1-AS1 | 0.572574 | 2.84E-48 | postive |
| TNFRSF25 | AC116914.2 | 0.572624 | 2.78E-48 | postive |
| TMIGD2 | AC002059.1 | 0.572726 | 2.65E-48 | postive |
| CTLA4 | TNRC6C-AS1 | 0.572779 | 2.59E-48 | postive |
| TMIGD2 | AC132192.1 | 0.572863 | 2.49E-48 | postive |
| TMIGD2 | PRC1-AS1 | 0.573001 | 2.33E-48 | postive |
| TMIGD2 | AC074032.1 | 0.573164 | 2.17E-48 | postive |
| TNFRSF25 | PLA2G4C-AS1 | 0.57335 | 1.99E-48 | postive |
| TMIGD2 | AC018557.2 | 0.573667 | 1.72E-48 | postive |
| TNFRSF25 | AC015813.1 | 0.573777 | 1.63E-48 | postive |
| TNFRSF25 | TPT1-AS1 | 0.573952 | 1.51E-48 | postive |
| TNFRSF25 | AP001107.4 | 0.574049 | 1.44E-48 | postive |
| TMIGD2 | AC022613.2 | 0.574217 | 1.33E-48 | postive |
| TNFRSF14 | AC040162.3 | 0.574261 | 1.31E-48 | postive |
| TNFRSF12A | AC093673.1 | 0.574389 | 1.23E-48 | postive |
| CD80 | AC087318.1 | 0.574457 | 1.19E-48 | postive |
| TMIGD2 | Z93241.1 | 0.575001 | 9.28E-49 | postive |
| CD80 | TRG-AS1 | 0.575208 | 8.43E-49 | postive |
| TMIGD2 | LINC01934 | 0.5753 | 8.08E-49 | postive |
| TNFRSF25 | ZNF32-AS1 | 0.575387 | 7.76E-49 | postive |
| CD28 | LINC00426 | 0.575412 | 7.67E-49 | postive |
| TMIGD2 | AC022154.1 | 0.575474 | 7.45E-49 | postive |
| TNFRSF25 | AC009133.1 | 0.575794 | 6.42E-49 | postive |
| TNFRSF9 | AC090152.1 | 0.575876 | 6.18E-49 | postive |
| TMIGD2 | AC105339.2 | 0.575942 | 6.00E-49 | postive |
| TNFRSF25 | AC015660.3 | 0.576157 | 5.43E-49 | postive |
| TMIGD2 | AP000688.1 | 0.576509 | 4.61E-49 | postive |
| LTA | AC010247.1 | 0.576546 | 4.53E-49 | postive |
| TNFRSF25 | LINC00921 | 0.576688 | 4.24E-49 | postive |
| TNFRSF25 | AC008870.2 | 0.57693 | 3.79E-49 | postive |
| CTLA4 | LINC01871 | 0.577052 | 3.58E-49 | postive |
| PDCD1LG2 | AC004816.1 | 0.577092 | 3.51E-49 | postive |
| TMIGD2 | AL121890.4 | 0.577131 | 3.45E-49 | postive |
| TMIGD2 | AC037198.2 | 0.577141 | 3.43E-49 | postive |
| TNFRSF14 | AC139530.1 | 0.577218 | 3.31E-49 | postive |
| TNFRSF14 | AL022328.1 | 0.5777 | 2.64E-49 | postive |

| | | | | |
|---|---|---|---|---|
| TNFRSF25 | NARF-IT1 | 0.577798 | 2.53E-49 | postive |
| PDCD1 | MIR155HG | 0.57781 | 2.51E-49 | postive |
| TNFSF13B | LINC00426 | 0.577828 | 2.49E-49 | postive |
| TNFRSF17 | AC109446.3 | 0.577857 | 2.46E-49 | postive |
| CD28 | TRG-AS1 | 0.577885 | 2.43E-49 | postive |
| TNFRSF13C | LINC02362 | 0.578658 | 1.69E-49 | postive |
| TNFRSF25 | AL136304.1 | 0.578663 | 1.68E-49 | postive |
| TNFRSF25 | AL390066.1 | 0.578695 | 1.66E-49 | postive |
| ICOS | AC079015.1 | 0.578841 | 1.55E-49 | postive |
| RELT | AC008105.3 | 0.578902 | 1.51E-49 | postive |
| TNFRSF14 | RUSC1-AS1 | 0.578918 | 1.49E-49 | postive |
| TNFRSF25 | LINC01355 | 0.579086 | 1.38E-49 | postive |
| TNFRSF25 | FAM13A-AS1 | 0.579087 | 1.38E-49 | postive |
| TNFRSF25 | AC008760.1 | 0.579262 | 1.27E-49 | postive |
| TNFSF8 | AL161785.1 | 0.579307 | 1.25E-49 | postive |
| CTLA4 | AC067945.3 | 0.579432 | 1.17E-49 | postive |
| TMIGD2 | AP000229.1 | 0.579503 | 1.14E-49 | postive |
| TNFRSF25 | AP001029.1 | 0.579511 | 1.13E-49 | postive |
| TNFRSF25 | AC093752.3 | 0.579641 | 1.06E-49 | postive |
| CD86 | AC002091.2 | 0.579733 | 1.02E-49 | postive |
| TNFRSF25 | ASB16-AS1 | 0.579785 | 9.95E-50 | postive |
| TMIGD2 | AC074029.3 | 0.580164 | 8.32E-50 | postive |
| TMIGD2 | AC060766.6 | 0.580524 | 7.02E-50 | postive |
| TNFRSF9 | AL158071.3 | 0.580552 | 6.93E-50 | postive |
| TMIGD2 | AC012676.4 | 0.580851 | 6.02E-50 | postive |
| TNFRSF25 | AC116667.1 | 0.580892 | 5.90E-50 | postive |
| TNFRSF25 | RNF139-AS1 | 0.580955 | 5.73E-50 | postive |
| PDCD1 | AL591468.1 | 0.581028 | 5.53E-50 | postive |
| TMIGD2 | AL583856.2 | 0.581094 | 5.36E-50 | postive |
| VTCN1 | LINC00092 | 0.581333 | 4.79E-50 | postive |
| TNFRSF25 | DM1-AS | 0.581335 | 4.79E-50 | postive |
| TNFRSF18 | AC109446.3 | 0.581403 | 4.63E-50 | postive |
| TNFRSF25 | LINC00106 | 0.581457 | 4.52E-50 | postive |
| TMIGD2 | AC004951.1 | 0.581927 | 3.61E-50 | postive |
| TNFRSF14 | AC109460.3 | 0.582217 | 3.15E-50 | postive |
| TNFRSF25 | AC073575.2 | 0.582441 | 2.83E-50 | postive |
| TMIGD2 | AC025682.1 | 0.582826 | 2.36E-50 | postive |
| TNFSF13B | TRG-AS1 | 0.582985 | 2.19E-50 | postive |
| TNFRSF25 | AL606760.2 | 0.583037 | 2.13E-50 | postive |
| TNFRSF25 | AP006284.1 | 0.583088 | 2.08E-50 | postive |
| PDCD1LG2 | PICSAR | 0.583127 | 2.04E-50 | postive |
| TMIGD2 | AC137630.1 | 0.583267 | 1.91E-50 | postive |
| TNFRSF25 | AL390728.6 | 0.583281 | 1.90E-50 | postive |
| EDAR | UCA1 | 0.583445 | 1.76E-50 | postive |
| TNFRSF25 | AC095057.3 | 0.583453 | 1.75E-50 | postive |
| TNFRSF14 | HCG27 | 0.583573 | 1.65E-50 | postive |
| TNFRSF25 | AL117379.1 | 0.583692 | 1.56E-50 | postive |
| TNFRSF25 | AC009690.2 | 0.583764 | 1.51E-50 | postive |
| TNFRSF25 | AP006623.1 | 0.5839 | 1.41E-50 | postive |
| ICOS | AL158071.3 | 0.583987 | 1.36E-50 | postive |
| TNFRSF25 | AL158834.2 | 0.584186 | 1.23E-50 | postive |
| CD80 | AL590764.1 | 0.584333 | 1.15E-50 | postive |
| TNFRSF13C | AC007569.1 | 0.584406 | 1.11E-50 | postive |
| TMIGD2 | AC022211.3 | 0.584466 | 1.08E-50 | postive |
| TNFRSF13C | AC104699.1 | 0.584901 | 8.76E-51 | postive |
| TNFRSF17 | PCED1B-AS1 | 0.585178 | 7.67E-51 | postive |
| TNFRSF14 | AC027601.1 | 0.585351 | 7.06E-51 | postive |

| | | | | |
|---|---|---|---|---|
| TMIGD2 | STK32A-AS1 | 0.58536 | 7.03E-51 | postive |
| TMIGD2 | LINC00862 | 0.585366 | 7.01E-51 | postive |
| TNFRSF25 | AL359504.2 | 0.585481 | 6.63E-51 | postive |
| TNFRSF9 | AC009133.3 | 0.585535 | 6.46E-51 | postive |
| TMIGD2 | AC016027.2 | 0.585705 | 5.96E-51 | postive |
| CD80 | TSPOAP1-AS1 | 0.585778 | 5.75E-51 | postive |
| TMIGD2 | AC025871.2 | 0.585935 | 5.33E-51 | postive |
| CD80 | AC083949.1 | 0.586389 | 4.28E-51 | postive |
| TNFRSF25 | AC105020.6 | 0.586392 | 4.28E-51 | postive |
| TNFRSF14 | CR559946.2 | 0.586789 | 3.53E-51 | postive |
| TMIGD2 | AL121999.1 | 0.587081 | 3.07E-51 | postive |
| TNFRSF25 | AC010327.5 | 0.587105 | 3.03E-51 | postive |
| ICOS | AC068196.1 | 0.58727 | 2.80E-51 | postive |
| TNFRSF25 | AC109460.2 | 0.587535 | 2.47E-51 | postive |
| TNFRSF1B | AC145098.1 | 0.587726 | 2.25E-51 | postive |
| TNFRSF25 | ZEB2-AS1 | 0.587796 | 2.17E-51 | postive |
| TNFRSF13C | AC007384.1 | 0.587948 | 2.02E-51 | postive |
| TMIGD2 | AL121944.1 | 0.588098 | 1.88E-51 | postive |
| TMIGD2 | AP001628.1 | 0.588143 | 1.84E-51 | postive |
| VTCN1 | LINC00511 | 0.588294 | 1.71E-51 | postive |
| TMIGD2 | AC005324.5 | 0.588506 | 1.54E-51 | postive |
| TMIGD2 | AC103923.1 | 0.58866 | 1.43E-51 | postive |
| LTB | USP30-AS1 | 0.588948 | 1.24E-51 | postive |
| TMIGD2 | AC026495.1 | 0.588951 | 1.24E-51 | postive |
| CTLA4 | AL683807.1 | 0.588979 | 1.22E-51 | postive |
| TNFRSF14 | AC104964.3 | 0.589075 | 1.17E-51 | postive |
| VTCN1 | AL357033.2 | 0.589194 | 1.10E-51 | postive |
| TNFRSF9 | LINC00539 | 0.589228 | 1.08E-51 | postive |
| CD80 | AC004494.1 | 0.589633 | 8.91E-52 | postive |
| TNFRSF25 | PSMA3-AS1 | 0.590116 | 7.04E-52 | postive |
| TMIGD2 | AL354892.3 | 0.590236 | 6.64E-52 | postive |
| TNFRSF25 | CCDC18-AS1 | 0.590443 | 6.00E-52 | postive |
| LTB | AC009133.3 | 0.590502 | 5.83E-52 | postive |
| TMIGD2 | AC096733.2 | 0.59065 | 5.42E-52 | postive |
| CD80 | LINC01094 | 0.591308 | 3.93E-52 | postive |
| CTLA4 | AC015911.3 | 0.591924 | 2.90E-52 | postive |
| TNFSF13 | LINC02038 | 0.591961 | 2.85E-52 | postive |
| CTLA4 | AC109446.3 | 0.592009 | 2.79E-52 | postive |
| PDCD1LG2 | LINC00941 | 0.592101 | 2.66E-52 | postive |
| TNFRSF14 | AC005306.1 | 0.592521 | 2.17E-52 | postive |
| TNFRSF25 | AC073655.2 | 0.592705 | 1.98E-52 | postive |
| CTLA4 | AL139352.1 | 0.592747 | 1.94E-52 | postive |
| VTCN1 | AC241644.2 | 0.593056 | 1.66E-52 | postive |
| TMIGD2 | RHOA-IT1 | 0.593111 | 1.62E-52 | postive |
| FASLG | AC068196.1 | 0.593111 | 1.62E-52 | postive |
| TNFRSF25 | LINC01772 | 0.593211 | 1.54E-52 | postive |
| TMIGD2 | ATP2B1-AS1 | 0.593558 | 1.30E-52 | postive |
| TMIGD2 | USP2-AS1 | 0.593721 | 1.20E-52 | postive |
| TMIGD2 | AP001107.1 | 0.593751 | 1.18E-52 | postive |
| TMIGD2 | AC099778.1 | 0.593824 | 1.14E-52 | postive |
| CTLA4 | AC068196.1 | 0.594097 | 9.95E-53 | postive |
| TMIGD2 | LINC02273 | 0.594504 | 8.14E-53 | postive |
| TMIGD2 | LINC02081 | 0.594916 | 6.64E-53 | postive |
| TNFRSF25 | AC010973.2 | 0.595103 | 6.05E-53 | postive |
| TNFRSF25 | AC092375.2 | 0.595143 | 5.93E-53 | postive |
| TNFRSF25 | AL021707.8 | 0.595483 | 5.01E-53 | postive |
| PDCD1 | AL158071.3 | 0.595896 | 4.08E-53 | postive |

| | | | | |
|---|---|---|---|---|
| TNFRSF25 | AC022144.1 | 0.596077 | 3.72E-53 | postive |
| TMIGD2 | FAM66C | 0.596101 | 3.68E-53 | postive |
| TMIGD2 | Z84484.1 | 0.596186 | 3.53E-53 | postive |
| TNFRSF25 | RFPL3S | 0.596198 | 3.51E-53 | postive |
| TNFRSF25 | AC012617.1 | 0.596201 | 3.50E-53 | postive |
| TNFRSF25 | HOXB-AS1 | 0.59631 | 3.32E-53 | postive |
| TNFRSF25 | AC087500.2 | 0.59668 | 2.76E-53 | postive |
| TNFRSF25 | AC026471.4 | 0.596984 | 2.37E-53 | postive |
| TNFRSF25 | AL590666.1 | 0.597495 | 1.83E-53 | postive |
| TNFRSF25 | AC074117.1 | 0.59756 | 1.78E-53 | postive |
| TNFRSF25 | AC004846.2 | 0.597742 | 1.62E-53 | postive |
| RELT | PCED1B-AS1 | 0.597807 | 1.57E-53 | postive |
| TMIGD2 | AL391095.1 | 0.597912 | 1.49E-53 | postive |
| VTCN1 | AC023669.1 | 0.598018 | 1.41E-53 | postive |
| TNFRSF25 | AL031186.1 | 0.598037 | 1.40E-53 | postive |
| TNFRSF8 | AC112721.2 | 0.598074 | 1.37E-53 | postive |
| TNFRSF25 | AL139123.1 | 0.598148 | 1.32E-53 | postive |
| CTLA4 | AC079015.1 | 0.598303 | 1.22E-53 | postive |
| TNFRSF1B | AC002091.1 | 0.598367 | 1.18E-53 | postive |
| TMIGD2 | AP002812.5 | 0.598404 | 1.16E-53 | postive |
| CD28 | AC004585.1 | 0.598499 | 1.11E-53 | postive |
| TNFRSF13C | LINC00926 | 0.598572 | 1.07E-53 | postive |
| TNFRSF25 | AL353622.1 | 0.598631 | 1.04E-53 | postive |
| TNFSF8 | AC006033.2 | 0.598798 | 9.54E-54 | postive |
| TMIGD2 | AC040977.1 | 0.598816 | 9.46E-54 | postive |
| TNFRSF25 | AL590096.1 | 0.598895 | 9.09E-54 | postive |
| TNFRSF14 | KMT2E-AS1 | 0.598925 | 8.95E-54 | postive |
| TNFRSF25 | TTC28-AS1 | 0.598979 | 8.71E-54 | postive |
| TNFRSF25 | AL662797.1 | 0.599088 | 8.25E-54 | postive |
| PDCD1 | AC009133.3 | 0.599096 | 8.21E-54 | postive |
| TNFRSF25 | YEATS2-AS1 | 0.599131 | 8.07E-54 | postive |
| ICOS | AL365361.1 | 0.59917 | 7.91E-54 | postive |
| TMIGD2 | AL008726.1 | 0.599422 | 6.97E-54 | postive |
| LTA | AC006369.1 | 0.599491 | 6.73E-54 | postive |
| TNFRSF25 | ZKSCAN2-DT | 0.599611 | 6.33E-54 | postive |
| TNFRSF25 | AC135178.1 | 0.599638 | 6.25E-54 | postive |
| TNFRSF25 | LINC01786 | 0.599645 | 6.23E-54 | postive |
| TNFRSF25 | AC010319.4 | 0.600279 | 4.52E-54 | postive |
| TMIGD2 | LINC02416 | 0.60029 | 4.50E-54 | postive |
| LTB | AC243960.1 | 0.600468 | 4.11E-54 | postive |
| TNFRSF25 | AC103691.1 | 0.600597 | 3.85E-54 | postive |
| LTA | AC243829.2 | 0.600641 | 3.76E-54 | postive |
| TMIGD2 | AC007216.2 | 0.600681 | 3.69E-54 | postive |
| TNFRSF14 | AC254562.3 | 0.600864 | 3.36E-54 | postive |
| TMIGD2 | AC016257.1 | 0.601204 | 2.83E-54 | postive |
| TNFRSF25 | ASMTL-AS1 | 0.601215 | 2.81E-54 | postive |
| FASLG | AL158071.3 | 0.60126 | 2.75E-54 | postive |
| CD28 | PCED1B-AS1 | 0.601627 | 2.28E-54 | postive |
| TMIGD2 | AC063943.1 | 0.601737 | 2.16E-54 | postive |
| CTLA4 | AC004771.1 | 0.601791 | 2.10E-54 | postive |
| TNFRSF14 | AL136295.7 | 0.602217 | 1.69E-54 | postive |
| TMIGD2 | AC025171.3 | 0.602512 | 1.46E-54 | postive |
| PDCD1 | LINC00158 | 0.60272 | 1.31E-54 | postive |
| TNFRSF13C | LINC00582 | 0.602752 | 1.29E-54 | postive |
| FASLG | AL139352.1 | 0.60283 | 1.24E-54 | postive |
| TNFRSF25 | AC234582.1 | 0.602843 | 1.23E-54 | postive |
| TNFRSF25 | AC048341.1 | 0.603101 | 1.08E-54 | postive |

| | | | | |
|---|---|---|---|---|
| TNFRSF25 | AC009120.2 | 0.603188 | 1.03E-54 | postive |
| TNFRSF25 | AL021707.3 | 0.603313 | 9.67E-55 | postive |
| FASLG | AC079015.1 | 0.603525 | 8.68E-55 | postive |
| TMIGD2 | AC114956.1 | 0.603605 | 8.33E-55 | postive |
| LTB | AC109446.3 | 0.603637 | 8.20E-55 | postive |
| TNFRSF25 | ZNF213-AS1 | 0.603833 | 7.41E-55 | postive |
| ICOS | LINC00861 | 0.603851 | 7.35E-55 | postive |
| TMIGD2 | AC010336.2 | 0.60389 | 7.20E-55 | postive |
| TNFRSF25 | BACE1-AS | 0.603998 | 6.81E-55 | postive |
| TMIGD2 | AC073517.1 | 0.604004 | 6.79E-55 | postive |
| TNFRSF25 | MIR503HG | 0.604264 | 5.94E-55 | postive |
| CTLA4 | AL591468.1 | 0.604335 | 5.73E-55 | postive |
| TMIGD2 | AC135178.5 | 0.604458 | 5.38E-55 | postive |
| CTLA4 | AC006369.1 | 0.604469 | 5.35E-55 | postive |
| TNFRSF25 | LINC00528 | 0.604507 | 5.25E-55 | postive |
| TMIGD2 | AP003086.2 | 0.605129 | 3.81E-55 | postive |
| TMIGD2 | AL356299.2 | 0.605205 | 3.67E-55 | postive |
| TMIGD2 | LINC00843 | 0.605339 | 3.42E-55 | postive |
| TNFRSF25 | AC005674.2 | 0.605484 | 3.18E-55 | postive |
| TNFRSF25 | AL136295.2 | 0.60569 | 2.86E-55 | postive |
| TMIGD2 | AL139289.2 | 0.605717 | 2.82E-55 | postive |
| TNFRSF25 | AC010976.2 | 0.605821 | 2.67E-55 | postive |
| TMIGD2 | AC005096.1 | 0.606073 | 2.34E-55 | postive |
| TNFRSF14 | ZEB2-AS1 | 0.606182 | 2.22E-55 | postive |
| TNFRSF14 | AC114730.3 | 0.606533 | 1.85E-55 | postive |
| TNFRSF25 | AC004466.1 | 0.606577 | 1.81E-55 | postive |
| TNFRSF14 | AL513320.1 | 0.606917 | 1.52E-55 | postive |
| TNFRSF25 | AC005253.1 | 0.60693 | 1.50E-55 | postive |
| TMIGD2 | AL591845.1 | 0.607042 | 1.42E-55 | postive |
| TMIGD2 | AC022400.1 | 0.60716 | 1.34E-55 | postive |
| TMIGD2 | AC108134.2 | 0.60729 | 1.25E-55 | postive |
| TNFRSF14 | AC087741.1 | 0.607506 | 1.12E-55 | postive |
| TNFRSF25 | AC004687.1 | 0.607512 | 1.11E-55 | postive |
| TMIGD2 | MZF1-AS1 | 0.6076 | 1.06E-55 | postive |
| TNFRSF25 | AC104564.3 | 0.607829 | 9.44E-56 | postive |
| TNFRSF25 | PTOV1-AS1 | 0.608118 | 8.13E-56 | postive |
| TNFRSF25 | AC022167.2 | 0.608161 | 7.95E-56 | postive |
| PDCD1 | AL590764.1 | 0.608278 | 7.48E-56 | postive |
| TMIGD2 | AC034102.5 | 0.608353 | 7.19E-56 | postive |
| TNFRSF25 | AC245884.8 | 0.608782 | 5.75E-56 | postive |
| TMIGD2 | AL359541.1 | 0.608837 | 5.59E-56 | postive |
| TNFRSF25 | AC016957.2 | 0.608963 | 5.23E-56 | postive |
| TNFRSF25 | IBA57-DT | 0.609062 | 4.97E-56 | postive |
| TNFRSF14 | AC009133.1 | 0.609454 | 4.05E-56 | postive |
| TMIGD2 | AC245884.9 | 0.609465 | 4.03E-56 | postive |
| TNFRSF25 | AC092143.3 | 0.6096 | 3.75E-56 | postive |
| TMIGD2 | LINC01976 | 0.609621 | 3.71E-56 | postive |
| VTCN1 | AC104072.1 | 0.609686 | 3.59E-56 | postive |
| PDCD1LG2 | AP005019.1 | 0.609767 | 3.44E-56 | postive |
| TNFSF13B | AC006033.2 | 0.61012 | 2.86E-56 | postive |
| CD80 | AC008957.1 | 0.610386 | 2.49E-56 | postive |
| FASLG | AC012645.3 | 0.610531 | 2.31E-56 | postive |
| TNFRSF14 | LINC01176 | 0.610653 | 2.16E-56 | postive |
| TNFRSF25 | AC005519.1 | 0.610914 | 1.89E-56 | postive |
| TMIGD2 | AL390729.1 | 0.610923 | 1.88E-56 | postive |
| TMIGD2 | AL445490.1 | 0.611093 | 1.72E-56 | postive |
| CD86 | AC011899.3 | 0.611214 | 1.61E-56 | postive |

| | | | | |
|---|---|---|---|---|
| TNFRSF25 | AC132872.1 | 0.611384 | 1.47E-56 | postive |
| TNFRSF25 | AC110285.2 | 0.611538 | 1.36E-56 | postive |
| TMIGD2 | HDHD5-AS1 | 0.611562 | 1.34E-56 | postive |
| TMIGD2 | AC079210.1 | 0.611693 | 1.25E-56 | postive |
| TNFRSF14 | AL135999.1 | 0.611783 | 1.19E-56 | postive |
| TNFRSF25 | AL117209.1 | 0.61193 | 1.11E-56 | postive |
| CD86 | AC006033.2 | 0.611988 | 1.07E-56 | postive |
| TNFRSF25 | AC233728.1 | 0.612153 | 9.83E-57 | postive |
| TNFSF8 | LINC02285 | 0.612266 | 9.26E-57 | postive |
| LTA | AC012645.3 | 0.612365 | 8.79E-57 | postive |
| TMIGD2 | AC010422.4 | 0.613036 | 6.16E-57 | postive |
| FASLG | LINC00539 | 0.613432 | 5.00E-57 | postive |
| TNFRSF1B | LINC02285 | 0.613779 | 4.16E-57 | postive |
| TMIGD2 | AC022431.1 | 0.613791 | 4.13E-57 | postive |
| LTA | PSMB8-AS1 | 0.613803 | 4.11E-57 | postive |
| TNFRSF14 | PTOV1-AS2 | 0.614086 | 3.53E-57 | postive |
| TMIGD2 | AC105105.4 | 0.614133 | 3.45E-57 | postive |
| ICOS | AL132642.1 | 0.61414 | 3.43E-57 | postive |
| TMIGD2 | RNF216-IT1 | 0.614254 | 3.23E-57 | postive |
| TMIGD2 | AC231981.1 | 0.614257 | 3.23E-57 | postive |
| TMIGD2 | AC007619.1 | 0.614294 | 3.16E-57 | postive |
| ICOS | LINC02084 | 0.614723 | 2.52E-57 | postive |
| PDCD1LG2 | AF196972.1 | 0.614724 | 2.52E-57 | postive |
| TMIGD2 | AP000763.3 | 0.614789 | 2.43E-57 | postive |
| TMIGD2 | AC015914.1 | 0.614828 | 2.38E-57 | postive |
| TNFRSF25 | AC010809.2 | 0.615211 | 1.94E-57 | postive |
| TMIGD2 | AC010463.3 | 0.615311 | 1.84E-57 | postive |
| FASLG | LINC02363 | 0.615392 | 1.76E-57 | postive |
| FASLG | MIR155HG | 0.615498 | 1.67E-57 | postive |
| TNFRSF14 | AL049795.1 | 0.615692 | 1.50E-57 | postive |
| TMIGD2 | AC007098.1 | 0.616001 | 1.27E-57 | postive |
| TNFRSF14 | AC010883.1 | 0.616008 | 1.27E-57 | postive |
| TNFRSF25 | AC022150.2 | 0.6164 | 1.03E-57 | postive |
| TMIGD2 | Z69666.1 | 0.616475 | 9.89E-58 | postive |
| TNFRSF18 | PCED1B-AS1 | 0.616528 | 9.61E-58 | postive |
| CD28 | AL365361.1 | 0.616763 | 8.47E-58 | postive |
| TNFRSF25 | COL4A2-AS1 | 0.616877 | 7.97E-58 | postive |
| TMIGD2 | AC084757.3 | 0.616906 | 7.84E-58 | postive |
| TNFRSF25 | AC005104.1 | 0.617172 | 6.80E-58 | postive |
| TNFRSF13C | LINC02576 | 0.617506 | 5.68E-58 | postive |
| TMIGD2 | AC073896.3 | 0.618112 | 4.10E-58 | postive |
| TMIGD2 | AL355432.1 | 0.618405 | 3.50E-58 | postive |
| TMIGD2 | AC008403.3 | 0.618471 | 3.38E-58 | postive |
| TNFRSF25 | AL354836.1 | 0.61851 | 3.31E-58 | postive |
| TNFRSF25 | AL356481.3 | 0.618521 | 3.29E-58 | postive |
| TNFRSF9 | AL590764.1 | 0.618769 | 2.88E-58 | postive |
| TNFRSF25 | AL161452.1 | 0.619037 | 2.49E-58 | postive |
| TNFRSF25 | AC008105.3 | 0.619335 | 2.12E-58 | postive |
| TMIGD2 | LINC01842 | 0.619567 | 1.87E-58 | postive |
| TNFRSF25 | AC010883.1 | 0.61977 | 1.68E-58 | postive |
| TMIGD2 | AC103810.5 | 0.619893 | 1.57E-58 | postive |
| TMIGD2 | AC103769.1 | 0.619929 | 1.54E-58 | postive |
| TNFRSF25 | AL391244.3 | 0.620128 | 1.38E-58 | postive |
| ICOS | LINC02363 | 0.620322 | 1.24E-58 | postive |
| TNFRSF9 | AC087318.1 | 0.620444 | 1.16E-58 | postive |
| TMIGD2 | AC018766.1 | 0.620524 | 1.11E-58 | postive |
| ICOS | AC004865.2 | 0.620748 | 9.85E-59 | postive |

| | | | | |
|---|---|---|---|---|
| TNFRSF25 | AC016773.1 | 0.620759 | 9.79E-59 | postive |
| TMIGD2 | AC022146.2 | 0.620897 | 9.08E-59 | postive |
| CTLA4 | LINC00528 | 0.620976 | 8.70E-59 | postive |
| TNFRSF25 | AL359921.2 | 0.621113 | 8.07E-59 | postive |
| TNFRSF25 | AL031705.1 | 0.621262 | 7.44E-59 | postive |
| TMIGD2 | LINC02289 | 0.621522 | 6.46E-59 | postive |
| ICOS | AF127936.1 | 0.621548 | 6.37E-59 | postive |
| VTCN1 | AC023669.2 | 0.621698 | 5.87E-59 | postive |
| TNFSF13B | AL590764.1 | 0.622143 | 4.60E-59 | postive |
| TMIGD2 | LINC01012 | 0.622167 | 4.54E-59 | postive |
| TNFRSF25 | AL096870.2 | 0.62239 | 4.02E-59 | postive |
| TMIGD2 | AC008050.1 | 0.622759 | 3.28E-59 | postive |
| TMIGD2 | AC024267.3 | 0.622809 | 3.20E-59 | postive |
| TNFRSF25 | ITGB2-AS1 | 0.622958 | 2.95E-59 | postive |
| TMIGD2 | AC106738.1 | 0.623175 | 2.62E-59 | postive |
| TNFRSF25 | AL035563.1 | 0.623324 | 2.41E-59 | postive |
| TMIGD2 | AP001381.1 | 0.623378 | 2.34E-59 | postive |
| ICOS | MIR155HG | 0.623558 | 2.12E-59 | postive |
| TNFRSF25 | AC254562.3 | 0.623571 | 2.10E-59 | postive |
| TNFRSF1B | AL590764.1 | 0.624155 | 1.53E-59 | postive |
| TNFRSF25 | AL022328.1 | 0.62417 | 1.51E-59 | postive |
| TNFRSF14 | AL109811.3 | 0.624579 | 1.21E-59 | postive |
| TNFRSF25 | SEMA3F-AS1 | 0.624598 | 1.20E-59 | postive |
| TNFRSF25 | AC012368.1 | 0.624953 | 9.83E-60 | postive |
| TNFRSF9 | AC068196.1 | 0.624992 | 9.62E-60 | postive |
| CTLA4 | AC087318.1 | 0.625098 | 9.07E-60 | postive |
| TNFRSF25 | AC009118.3 | 0.625113 | 9.00E-60 | postive |
| TNFRSF25 | AL360181.2 | 0.625454 | 7.45E-60 | postive |
| TMIGD2 | AC005546.1 | 0.625717 | 6.44E-60 | postive |
| TMIGD2 | AC113383.1 | 0.62587 | 5.92E-60 | postive |
| TMIGD2 | AC124242.1 | 0.625882 | 5.88E-60 | postive |
| TNFRSF25 | AL645940.1 | 0.625948 | 5.67E-60 | postive |
| TMIGD2 | AC092802.2 | 0.626091 | 5.23E-60 | postive |
| TMIGD2 | AP000873.4 | 0.626472 | 4.24E-60 | postive |
| TMIGD2 | AC008083.2 | 0.626496 | 4.18E-60 | postive |
| TMIGD2 | AC124248.1 | 0.626538 | 4.08E-60 | postive |
| TNFRSF25 | DPP9-AS1 | 0.626784 | 3.56E-60 | postive |
| TMIGD2 | DENND5B-AS1 | 0.626822 | 3.49E-60 | postive |
| TMIGD2 | AL359265.3 | 0.62694 | 3.27E-60 | postive |
| PDCD1 | AC007728.2 | 0.626965 | 3.22E-60 | postive |
| PDCD1 | AC243829.2 | 0.627052 | 3.07E-60 | postive |
| TMIGD2 | AC104971.3 | 0.627198 | 2.83E-60 | postive |
| TNFRSF25 | HM13-IT1 | 0.62764 | 2.21E-60 | postive |
| LTA | AL139352.1 | 0.627677 | 2.17E-60 | postive |
| TNFRSF25 | CTBP1-AS | 0.628098 | 1.71E-60 | postive |
| TNFSF4 | BHLHE40-AS1 | 0.628295 | 1.53E-60 | postive |
| TNFRSF25 | PDXDC2P-NPIPB14P | 0.628444 | 1.41E-60 | postive |
| FASLG | PSMB8-AS1 | 0.629565 | 7.53E-61 | postive |
| TMIGD2 | AC027279.1 | 0.62993 | 6.14E-61 | postive |
| TNFRSF25 | PRKCZ-AS1 | 0.630019 | 5.84E-61 | postive |
| TMIGD2 | AC106820.4 | 0.630253 | 5.12E-61 | postive |
| TNFRSF25 | ZNF32-AS2 | 0.630635 | 4.13E-61 | postive |
| TMIGD2 | JARID2-AS1 | 0.63088 | 3.59E-61 | postive |
| TNFRSF25 | ZNF436-AS1 | 0.631174 | 3.04E-61 | postive |
| TMIGD2 | AP003170.3 | 0.631196 | 3.01E-61 | postive |
| TMIGD2 | AC091965.1 | 0.631429 | 2.63E-61 | postive |
| CTLA4 | PSMB8-AS1 | 0.631866 | 2.06E-61 | postive |

| | | | | |
|---|---|---|---|---|
| TNFRSF14 | MIR210HG | 0.63198 | 1.93E-61 | postive |
| TNFRSF25 | AP000347.2 | 0.632639 | 1.33E-61 | postive |
| TNFRSF25 | CACTIN-AS1 | 0.632679 | 1.30E-61 | postive |
| TNFRSF8 | AL139393.2 | 0.632802 | 1.21E-61 | postive |
| CD86 | PCED1B-AS1 | 0.632891 | 1.15E-61 | postive |
| TMIGD2 | AL450384.2 | 0.632973 | 1.10E-61 | postive |
| CTLA4 | AC243829.2 | 0.633042 | 1.06E-61 | postive |
| TMIGD2 | AC027319.1 | 0.633219 | 9.54E-62 | postive |
| TNFRSF25 | AC138028.4 | 0.633506 | 8.10E-62 | postive |
| CD80 | AC015911.3 | 0.633837 | 6.71E-62 | postive |
| TMIGD2 | AC004039.1 | 0.634161 | 5.58E-62 | postive |
| PDCD1 | USP30-AS1 | 0.634238 | 5.34E-62 | postive |
| CD80 | LINC00539 | 0.634517 | 4.55E-62 | postive |
| TMIGD2 | AL158847.1 | 0.634676 | 4.15E-62 | postive |
| LTB | AC004585.1 | 0.634866 | 3.73E-62 | postive |
| TMIGD2 | AL139021.1 | 0.635045 | 3.36E-62 | postive |
| TMIGD2 | AP001269.2 | 0.635172 | 3.13E-62 | postive |
| TMIGD2 | AC100830.1 | 0.635894 | 2.07E-62 | postive |
| TNFRSF25 | AC004771.1 | 0.636031 | 1.91E-62 | postive |
| PDCD1LG2 | AC083967.1 | 0.636065 | 1.87E-62 | postive |
| TNFRSF25 | AL021707.6 | 0.636116 | 1.82E-62 | postive |
| TMIGD2 | AL359697.1 | 0.63623 | 1.70E-62 | postive |
| TNFSF13B | AC011899.2 | 0.636247 | 1.69E-62 | postive |
| TNFRSF13C | AC012236.1 | 0.636418 | 1.53E-62 | postive |
| CD86 | AF127936.1 | 0.636468 | 1.49E-62 | postive |
| VTCN1 | LINC01896 | 0.636525 | 1.44E-62 | postive |
| TNFRSF17 | AL365361.1 | 0.636737 | 1.27E-62 | postive |
| TNFRSF4 | AC007998.3 | 0.636936 | 1.13E-62 | postive |
| TNFRSF25 | LINC00893 | 0.636992 | 1.10E-62 | postive |
| TMIGD2 | AC008667.1 | 0.637053 | 1.06E-62 | postive |
| TMIGD2 | AL354809.1 | 0.637496 | 8.20E-63 | postive |
| ICOS | AC243960.1 | 0.63784 | 6.72E-63 | postive |
| TNFRSF25 | AC132872.2 | 0.638147 | 5.63E-63 | postive |
| TNFRSF25 | MHENCR | 0.638712 | 4.05E-63 | postive |
| CTLA4 | USP30-AS1 | 0.638746 | 3.98E-63 | postive |
| CD80 | AC007728.2 | 0.639232 | 3.00E-63 | postive |
| TMIGD2 | AC114271.1 | 0.639248 | 2.97E-63 | postive |
| TNFRSF25 | AC107375.1 | 0.639574 | 2.46E-63 | postive |
| TMIGD2 | LINC02544 | 0.640217 | 1.69E-63 | postive |
| TMIGD2 | AC008870.4 | 0.640599 | 1.35E-63 | postive |
| TNFRSF25 | U62317.2 | 0.640647 | 1.31E-63 | postive |
| TNFRSF25 | RAD51-AS1 | 0.64072 | 1.26E-63 | postive |
| ICOS | AC009133.3 | 0.640759 | 1.23E-63 | postive |
| TNFRSF25 | AC008764.8 | 0.641109 | 1.00E-63 | postive |
| TNFSF13B | AF127936.1 | 0.641125 | 9.92E-64 | postive |
| TMIGD2 | FAM222A-AS1 | 0.641156 | 9.74E-64 | postive |
| TNFRSF25 | AL049780.1 | 0.641442 | 8.24E-64 | postive |
| TMIGD2 | AL137779.1 | 0.641883 | 6.36E-64 | postive |
| TMIGD2 | BMPR1B-DT | 0.641894 | 6.31E-64 | postive |
| TMIGD2 | AL355512.1 | 0.642028 | 5.84E-64 | postive |
| TMIGD2 | AL139274.2 | 0.642577 | 4.23E-64 | postive |
| TNFRSF8 | RNF144A-AS1 | 0.642774 | 3.76E-64 | postive |
| TNFRSF25 | AL512770.1 | 0.642958 | 3.38E-64 | postive |
| ICOS | LINC00539 | 0.643021 | 3.25E-64 | postive |
| TNFSF8 | AC011899.2 | 0.643137 | 3.04E-64 | postive |
| TNFRSF25 | AC024361.3 | 0.643236 | 2.86E-64 | postive |
| TNFRSF1B | AC011899.2 | 0.643658 | 2.23E-64 | postive |

| | | | | |
|---|---|---|---|---|
| TMIGD2 | AC004000.1 | 0.64378 | 2.08E-64 | postive |
| TMIGD2 | AC113143.1 | 0.644208 | 1.61E-64 | postive |
| TMIGD2 | AC104653.1 | 0.644328 | 1.50E-64 | postive |
| TNFRSF25 | AC232271.1 | 0.644493 | 1.36E-64 | postive |
| TNFRSF25 | AC011462.4 | 0.644699 | 1.20E-64 | postive |
| TMIGD2 | LINC02550 | 0.645013 | 9.99E-65 | postive |
| PDCD1 | AL135818.1 | 0.645159 | 9.16E-65 | postive |
| TMIGD2 | AL512274.1 | 0.645229 | 8.79E-65 | postive |
| PDCD1LG2 | AC005291.2 | 0.645838 | 6.11E-65 | postive |
| TMIGD2 | AL354872.2 | 0.646055 | 5.37E-65 | postive |
| TNFRSF25 | AL513218.1 | 0.646309 | 4.61E-65 | postive |
| CD86 | AC090559.1 | 0.64646 | 4.21E-65 | postive |
| TNFRSF25 | AC127502.2 | 0.646752 | 3.54E-65 | postive |
| TNFRSF25 | AC016737.1 | 0.646793 | 3.45E-65 | postive |
| ICOS | AC087318.1 | 0.647059 | 2.95E-65 | postive |
| CTLA4 | LINC00539 | 0.647087 | 2.90E-65 | postive |
| CD86 | AL590764.1 | 0.647141 | 2.80E-65 | postive |
| CTLA4 | ITGB2-AS1 | 0.647328 | 2.51E-65 | postive |
| TNFRSF25 | AL513320.1 | 0.647653 | 2.06E-65 | postive |
| TNFRSF14 | AL022328.2 | 0.647935 | 1.74E-65 | postive |
| TNFRSF25 | AC084018.1 | 0.648059 | 1.62E-65 | postive |
| TNFRSF14 | AL365330.1 | 0.648216 | 1.47E-65 | postive |
| TMIGD2 | AC092112.1 | 0.648628 | 1.15E-65 | postive |
| TNFRSF25 | AC100803.3 | 0.64919 | 8.17E-66 | postive |
| TNFRSF25 | GUSBP11 | 0.64944 | 7.02E-66 | postive |
| TNFRSF9 | AC004865.2 | 0.649723 | 5.92E-66 | postive |
| TMIGD2 | AC023389.1 | 0.64973 | 5.89E-66 | postive |
| TNFSF8 | LINC01094 | 0.64995 | 5.16E-66 | postive |
| TNFRSF25 | AL109659.2 | 0.649957 | 5.14E-66 | postive |
| LTA | LINC00158 | 0.650226 | 4.37E-66 | postive |
| TNFRSF9 | PCED1B-AS1 | 0.650423 | 3.87E-66 | postive |
| TNFRSF25 | AC090589.3 | 0.650455 | 3.80E-66 | postive |
| TNFRSF25 | Z69706.1 | 0.650754 | 3.17E-66 | postive |
| TNFRSF25 | AC005332.5 | 0.650852 | 2.98E-66 | postive |
| TMIGD2 | LINC01252 | 0.650863 | 2.96E-66 | postive |
| TMIGD2 | AC011481.1 | 0.651292 | 2.28E-66 | postive |
| TNFSF8 | AL133371.2 | 0.651471 | 2.05E-66 | postive |
| TNFRSF25 | AC109460.3 | 0.651653 | 1.83E-66 | postive |
| TNFRSF25 | AC108134.3 | 0.652888 | 8.61E-67 | postive |
| LTB | AC004865.2 | 0.65316 | 7.29E-67 | postive |
| TNFRSF25 | AC073335.2 | 0.65318 | 7.21E-67 | postive |
| PDCD1 | AC243960.1 | 0.653225 | 7.01E-67 | postive |
| CTLA4 | LINC00861 | 0.653322 | 6.60E-67 | postive |
| TMIGD2 | AC067930.3 | 0.653739 | 5.11E-67 | postive |
| TNFSF13B | PCED1B-AS1 | 0.654213 | 3.82E-67 | postive |
| FASLG | LINC00158 | 0.654264 | 3.70E-67 | postive |
| TMIGD2 | AL136418.1 | 0.654372 | 3.46E-67 | postive |
| ICOS | USP30-AS1 | 0.655135 | 2.16E-67 | postive |
| TMIGD2 | AC116914.2 | 0.655485 | 1.74E-67 | postive |
| TMIGD2 | AC092919.1 | 0.655697 | 1.53E-67 | postive |
| TNFRSF25 | AL022328.3 | 0.655934 | 1.32E-67 | postive |
| TNFRSF17 | OSTN-AS1 | 0.6561 | 1.19E-67 | postive |
| TMIGD2 | AC026471.5 | 0.656244 | 1.09E-67 | postive |
| CTLA4 | TSPOAP1-AS1 | 0.656522 | 9.16E-68 | postive |
| TMIGD2 | AC015849.4 | 0.657287 | 5.69E-68 | postive |
| VTCN1 | AC005082.1 | 0.657294 | 5.67E-68 | postive |
| TNFRSF25 | TNRC6C-AS1 | 0.657557 | 4.81E-68 | postive |

| | | | | |
|---|---|---|---|---|
| TMIGD2 | AC245297.2 | 0.657607 | 4.66E-68 | postive |
| ICOS | AC015911.3 | 0.657996 | 3.66E-68 | postive |
| PDCD1LG2 | LINC02154 | 0.658133 | 3.36E-68 | postive |
| TMIGD2 | AC133550.2 | 0.6583 | 3.02E-68 | postive |
| TNFRSF25 | AC027796.4 | 0.65831 | 3.01E-68 | postive |
| FASLG | AC243960.1 | 0.65847 | 2.72E-68 | postive |
| TMIGD2 | AL050327.1 | 0.65859 | 2.52E-68 | postive |
| TMIGD2 | AC007216.3 | 0.659237 | 1.68E-68 | postive |
| TMIGD2 | AC000120.1 | 0.65933 | 1.59E-68 | postive |
| TNFRSF25 | LINC00926 | 0.659345 | 1.57E-68 | postive |
| TNFRSF14 | TNFRSF14-AS1 | 0.659397 | 1.52E-68 | postive |
| CD86 | AL591468.1 | 0.659683 | 1.27E-68 | postive |
| TNFRSF25 | AC129510.1 | 0.659696 | 1.26E-68 | postive |
| TNFRSF14 | AL139246.3 | 0.659863 | 1.13E-68 | postive |
| TMIGD2 | AL513477.2 | 0.660406 | 8.06E-69 | postive |
| TNFRSF25 | LENG8-AS1 | 0.660493 | 7.63E-69 | postive |
| CTLA4 | LINC00158 | 0.660533 | 7.44E-69 | postive |
| TMIGD2 | AC006238.1 | 0.660724 | 6.59E-69 | postive |
| TNFRSF25 | AC003070.1 | 0.661062 | 5.33E-69 | postive |
| TNFRSF25 | AL022322.1 | 0.66116 | 5.00E-69 | postive |
| CTLA4 | LINC02084 | 0.662509 | 2.13E-69 | postive |
| TMIGD2 | AC025576.2 | 0.662637 | 1.96E-69 | postive |
| TNFRSF25 | AC020931.1 | 0.662716 | 1.87E-69 | postive |
| TMIGD2 | ZFHX2-AS1 | 0.662829 | 1.74E-69 | postive |
| PDCD1 | AC243829.4 | 0.663025 | 1.53E-69 | postive |
| LTB | AL590764.1 | 0.663074 | 1.49E-69 | postive |
| TNFRSF25 | SNHG20 | 0.663176 | 1.39E-69 | postive |
| TMIGD2 | AC012653.2 | 0.663381 | 1.22E-69 | postive |
| PDCD1LG2 | AC004817.3 | 0.664214 | 7.18E-70 | postive |
| TMIGD2 | AC005776.2 | 0.664382 | 6.45E-70 | postive |
| TMIGD2 | AC073311.1 | 0.664417 | 6.30E-70 | postive |
| TNFRSF14 | AC007292.1 | 0.664443 | 6.20E-70 | postive |
| TMIGD2 | AC136475.5 | 0.664525 | 5.88E-70 | postive |
| CTLA4 | LINC02422 | 0.664593 | 5.63E-70 | postive |
| TMIGD2 | NUCB1-AS1 | 0.664713 | 5.21E-70 | postive |
| PDCD1 | BHLHE40-AS1 | 0.664802 | 4.93E-70 | postive |
| PDCD1LG2 | AL365356.5 | 0.665222 | 3.76E-70 | postive |
| TNFRSF25 | STAG3L5P-PVRIG2P-PILRB | 0.665417 | 3.32E-70 | postive |
| TMIGD2 | ASAP1-IT2 | 0.665655 | 2.85E-70 | postive |
| TNFRSF25 | AL928654.2 | 0.665697 | 2.77E-70 | postive |
| TMIGD2 | SAP30L-AS1 | 0.665971 | 2.32E-70 | postive |
| TMIGD2 | LINC01827 | 0.666286 | 1.90E-70 | postive |
| PDCD1 | AC010247.2 | 0.666849 | 1.32E-70 | postive |
| TMIGD2 | AL035461.2 | 0.666979 | 1.21E-70 | postive |
| TMIGD2 | ARF4-AS1 | 0.667009 | 1.19E-70 | postive |
| ICOS | PCED1B-AS1 | 0.667027 | 1.17E-70 | postive |
| TMIGD2 | AC002116.2 | 0.66716 | 1.08E-70 | postive |
| LTA | TSPOAP1-AS1 | 0.66721 | 1.04E-70 | postive |
| TNFRSF25 | AL162586.1 | 0.667242 | 1.02E-70 | postive |
| TMIGD2 | HCG25 | 0.668358 | 4.96E-71 | postive |
| TNFRSF25 | AL031658.1 | 0.668502 | 4.51E-71 | postive |
| TNFRSF14 | AP000347.2 | 0.668544 | 4.39E-71 | postive |
| TNFRSF1B | PCED1B-AS1 | 0.668676 | 4.03E-71 | postive |
| TNFRSF25 | AC006435.2 | 0.668782 | 3.76E-71 | postive |
| CD80 | AL591468.1 | 0.669221 | 2.83E-71 | postive |
| ICOS | AL590764.1 | 0.669732 | 2.02E-71 | postive |
| TNFRSF25 | AC005387.1 | 0.669845 | 1.88E-71 | postive |

| | | | | |
|---|---|---|---|---|
| TNFRSF25 | AC069281.2 | 0.669874 | 1.84E-71 | postive |
| TNFRSF25 | AC005785.1 | 0.670303 | 1.39E-71 | postive |
| TMIGD2 | AC104806.2 | 0.670501 | 1.22E-71 | postive |
| TMIGD2 | SPIN4-AS1 | 0.670766 | 1.03E-71 | postive |
| TNFRSF9 | LINC02084 | 0.671342 | 7.04E-72 | postive |
| TMIGD2 | AL512306.2 | 0.671661 | 5.71E-72 | postive |
| CTLA4 | AL135818.2 | 0.671755 | 5.37E-72 | postive |
| FASLG | TSPOAP1-AS1 | 0.672264 | 3.84E-72 | postive |
| FASLG | AC009133.3 | 0.672441 | 3.41E-72 | postive |
| TNFRSF25 | CR559946.2 | 0.673118 | 2.18E-72 | postive |
| TMIGD2 | AC010524.1 | 0.673182 | 2.09E-72 | postive |
| TMIGD2 | FGF12-AS2 | 0.674047 | 1.18E-72 | postive |
| TNFRSF25 | AP001160.1 | 0.674153 | 1.10E-72 | postive |
| TNFRSF25 | AC004253.1 | 0.674406 | 9.27E-73 | postive |
| TMIGD2 | AL357874.1 | 0.67477 | 7.27E-73 | postive |
| TNFRSF25 | AC005264.1 | 0.674784 | 7.21E-73 | postive |
| TNFRSF25 | AL049795.1 | 0.674856 | 6.87E-73 | postive |
| TNFRSF25 | AC055855.1 | 0.675341 | 4.97E-73 | postive |
| TMIGD2 | LINC02572 | 0.675474 | 4.54E-73 | postive |
| TMIGD2 | AC008946.1 | 0.675611 | 4.15E-73 | postive |
| TMIGD2 | PCAT1 | 0.67566 | 4.02E-73 | postive |
| TMIGD2 | AC098487.1 | 0.675877 | 3.47E-73 | postive |
| TMIGD2 | DNAJC27-AS1 | 0.676172 | 2.85E-73 | postive |
| TMIGD2 | AL132639.2 | 0.676285 | 2.64E-73 | postive |
| TNFRSF25 | PKD1P6-NPIPP1 | 0.676349 | 2.53E-73 | postive |
| PDCD1LG2 | AC004817.2 | 0.676426 | 2.40E-73 | postive |
| CTLA4 | AL590764.1 | 0.676454 | 2.36E-73 | postive |
| TMIGD2 | AC115102.1 | 0.676501 | 2.28E-73 | postive |
| TNFRSF9 | TSPOAP1-AS1 | 0.677097 | 1.53E-73 | postive |
| TMIGD2 | AL109936.2 | 0.677124 | 1.50E-73 | postive |
| TMIGD2 | AC012485.2 | 0.677292 | 1.34E-73 | postive |
| TMIGD2 | LINC01715 | 0.67734 | 1.30E-73 | postive |
| TNFRSF25 | AC004918.1 | 0.677996 | 8.35E-74 | postive |
| TMIGD2 | AL360091.1 | 0.678006 | 8.30E-74 | postive |
| TNFSF8 | AC090559.1 | 0.678594 | 5.57E-74 | postive |
| TMIGD2 | AC096642.1 | 0.678818 | 4.79E-74 | postive |
| TNFRSF25 | MMP25-AS1 | 0.678899 | 4.53E-74 | postive |
| PDCD1 | AC004865.2 | 0.678958 | 4.36E-74 | postive |
| TMIGD2 | RAD21-AS1 | 0.679248 | 3.58E-74 | postive |
| PDCD1 | LINC02084 | 0.679463 | 3.09E-74 | postive |
| PDCD1 | TRG-AS1 | 0.679971 | 2.19E-74 | postive |
| TMIGD2 | AL024508.2 | 0.680412 | 1.62E-74 | postive |
| LTA | AC243829.4 | 0.680749 | 1.29E-74 | postive |
| TNFRSF9 | LINC00158 | 0.680819 | 1.23E-74 | postive |
| TNFSF8 | AF127936.1 | 0.680855 | 1.20E-74 | postive |
| TNFRSF25 | AL031714.1 | 0.681593 | 7.23E-75 | postive |
| TNFRSF25 | LINC01160 | 0.682032 | 5.35E-75 | postive |
| CTLA4 | AC243829.4 | 0.682063 | 5.24E-75 | postive |
| LTA | AC243960.1 | 0.682605 | 3.61E-75 | postive |
| ICOS | LINC00158 | 0.683031 | 2.69E-75 | postive |
| TMIGD2 | AC005730.3 | 0.683142 | 2.49E-75 | postive |
| TMIGD2 | HNF4A-AS1 | 0.683592 | 1.83E-75 | postive |
| TMIGD2 | AC002398.1 | 0.683722 | 1.67E-75 | postive |
| TMIGD2 | AC051619.7 | 0.683758 | 1.63E-75 | postive |
| TNFRSF25 | AC009283.1 | 0.683792 | 1.59E-75 | postive |
| RELT | AC145098.1 | 0.684052 | 1.33E-75 | postive |
| TMIGD2 | AL031722.1 | 0.684107 | 1.28E-75 | postive |

| | | | | |
|---|---|---|---|---|
| TNFRSF25 | C9orf139 | 0.684198 | 1.20E-75 | postive |
| CTLA4 | AL135818.1 | 0.684371 | 1.07E-75 | postive |
| CTLA4 | AC012645.3 | 0.684609 | 9.05E-76 | postive |
| TNFSF13B | LINC01094 | 0.684827 | 7.78E-76 | postive |
| TMIGD2 | AC106864.1 | 0.685252 | 5.79E-76 | postive |
| TMIGD2 | AC044840.1 | 0.685325 | 5.50E-76 | postive |
| TMIGD2 | AP000255.1 | 0.685719 | 4.18E-76 | postive |
| TMIGD2 | AC021491.4 | 0.685776 | 4.02E-76 | postive |
| TNFRSF25 | AC087289.2 | 0.686478 | 2.46E-76 | postive |
| TMIGD2 | AL592301.1 | 0.686514 | 2.40E-76 | postive |
| CD86 | AC002091.1 | 0.686596 | 2.27E-76 | postive |
| TNFRSF25 | AC135050.3 | 0.686619 | 2.23E-76 | postive |
| FASLG | AL590764.1 | 0.686713 | 2.09E-76 | postive |
| TMIGD2 | LINC00336 | 0.686985 | 1.73E-76 | postive |
| TMIGD2 | IDH2-DT | 0.688549 | 5.76E-77 | postive |
| TNFRSF9 | AC007728.2 | 0.688708 | 5.15E-77 | postive |
| TMIGD2 | AC002310.1 | 0.688726 | 5.09E-77 | postive |
| TMIGD2 | AC078785.1 | 0.688965 | 4.30E-77 | postive |
| TMIGD2 | AC135050.4 | 0.689468 | 3.01E-77 | postive |
| CTLA4 | MIR155HG | 0.689741 | 2.48E-77 | postive |
| FASLG | AC243829.2 | 0.68997 | 2.11E-77 | postive |
| TMIGD2 | AC079313.1 | 0.690171 | 1.83E-77 | postive |
| TNFSF13B | AL591468.1 | 0.690686 | 1.27E-77 | postive |
| CTLA4 | AC004865.2 | 0.69075 | 1.21E-77 | postive |
| TNFRSF25 | LINC00115 | 0.690831 | 1.15E-77 | postive |
| TMIGD2 | AC079414.3 | 0.691065 | 9.70E-78 | postive |
| LTA | LINC02084 | 0.691411 | 7.58E-78 | postive |
| TMIGD2 | AL034417.2 | 0.692495 | 3.50E-78 | postive |
| PDCD1 | LINC00426 | 0.69295 | 2.52E-78 | postive |
| TMIGD2 | AC023043.4 | 0.693193 | 2.12E-78 | postive |
| TMIGD2 | TOB1-AS1 | 0.693218 | 2.08E-78 | postive |
| TMIGD2 | AC074135.1 | 0.693382 | 1.85E-78 | postive |
| TNFRSF25 | AC008735.2 | 0.693473 | 1.73E-78 | postive |
| TNFRSF25 | LINC01176 | 0.693657 | 1.52E-78 | postive |
| TNFRSF25 | LINC00174 | 0.693664 | 1.51E-78 | postive |
| TMIGD2 | LINC01356 | 0.693769 | 1.40E-78 | postive |
| ICOS | AC243829.2 | 0.693828 | 1.34E-78 | postive |
| TNFRSF25 | SH3BP5-AS1 | 0.694418 | 8.77E-79 | postive |
| TMIGD2 | AC020913.3 | 0.694744 | 6.93E-79 | postive |
| TNFRSF25 | AL365330.1 | 0.694776 | 6.77E-79 | postive |
| CTLA4 | AC008105.3 | 0.69478 | 6.76E-79 | postive |
| TMIGD2 | AC090515.5 | 0.695119 | 5.29E-79 | postive |
| TNFRSF25 | AL136295.7 | 0.695636 | 3.63E-79 | postive |
| TMIGD2 | AC092720.1 | 0.695638 | 3.63E-79 | postive |
| TNFRSF25 | AL139287.1 | 0.696029 | 2.73E-79 | postive |
| CTLA4 | BHLHE40-AS1 | 0.696089 | 2.62E-79 | postive |
| TMIGD2 | AC011477.4 | 0.696651 | 1.74E-79 | postive |
| TMIGD2 | AC114488.1 | 0.696701 | 1.68E-79 | postive |
| TNFSF8 | AC002091.2 | 0.696743 | 1.63E-79 | postive |
| TMIGD2 | AC026904.2 | 0.697066 | 1.28E-79 | postive |
| TMIGD2 | AL022311.1 | 0.697742 | 7.84E-80 | postive |
| TMIGD2 | AC005014.2 | 0.69782 | 7.40E-80 | postive |
| TNFRSF9 | LINC02363 | 0.698131 | 5.89E-80 | postive |
| TMIGD2 | AC009090.1 | 0.69833 | 5.09E-80 | postive |
| FASLG | AC004865.2 | 0.698333 | 5.08E-80 | postive |
| CD80 | LINC00426 | 0.699047 | 3.01E-80 | postive |
| TMIGD2 | AL023881.1 | 0.699568 | 2.05E-80 | postive |

| | | | | |
|---|---|---|---|---|
| TMIGD2 | AL731577.1 | 0.699749 | 1.79E-80 | postive |
| TMIGD2 | AL359643.2 | 0.700162 | 1.32E-80 | postive |
| TMIGD2 | AC008429.1 | 0.700219 | 1.27E-80 | postive |
| CD80 | AF127936.1 | 0.700669 | 9.09E-81 | postive |
| TMIGD2 | AC108471.2 | 0.700699 | 8.89E-81 | postive |
| TMIGD2 | AC018521.5 | 0.700753 | 8.54E-81 | postive |
| TNFRSF9 | USP30-AS1 | 0.701124 | 6.49E-81 | postive |
| CD86 | LINC02285 | 0.701162 | 6.31E-81 | postive |
| LTA | AC009133.3 | 0.701805 | 3.91E-81 | postive |
| TNFRSF25 | AC092118.2 | 0.701985 | 3.42E-81 | postive |
| TMIGD2 | AC010519.1 | 0.702412 | 2.49E-81 | postive |
| TMIGD2 | AL157904.1 | 0.702503 | 2.33E-81 | postive |
| TMIGD2 | AC107959.1 | 0.702868 | 1.77E-81 | postive |
| ICOS | TSPOAP1-AS1 | 0.703107 | 1.48E-81 | postive |
| TNFRSF25 | AC245052.4 | 0.703482 | 1.12E-81 | postive |
| TMIGD2 | AL022316.1 | 0.70388 | 8.30E-82 | postive |
| TNFRSF25 | LINC01089 | 0.703895 | 8.20E-82 | postive |
| FASLG | LINC02084 | 0.703971 | 7.75E-82 | postive |
| TMIGD2 | AC079907.1 | 0.703994 | 7.62E-82 | postive |
| TMIGD2 | AC135178.3 | 0.704951 | 3.71E-82 | postive |
| TMIGD2 | SRD5A3-AS1 | 0.705749 | 2.03E-82 | postive |
| TMIGD2 | AP001207.3 | 0.705778 | 1.98E-82 | postive |
| TNFRSF25 | AP002807.1 | 0.706295 | 1.34E-82 | postive |
| TMIGD2 | AC010761.6 | 0.706528 | 1.12E-82 | postive |
| TMIGD2 | UBOX5-AS1 | 0.706582 | 1.08E-82 | postive |
| TMIGD2 | AC009509.4 | 0.706656 | 1.02E-82 | postive |
| TMIGD2 | AC131009.1 | 0.706677 | 1.00E-82 | postive |
| TMIGD2 | AC008663.1 | 0.707883 | 4.01E-83 | postive |
| TMIGD2 | AC109597.2 | 0.707937 | 3.85E-83 | postive |
| TMIGD2 | NAV2-AS3 | 0.708073 | 3.47E-83 | postive |
| TMIGD2 | AC112491.1 | 0.708082 | 3.44E-83 | postive |
| TMIGD2 | AC079336.5 | 0.708344 | 2.82E-83 | postive |
| TNFRSF25 | AC012615.6 | 0.708447 | 2.60E-83 | postive |
| TNFRSF25 | RUSC1-AS1 | 0.709509 | 1.15E-83 | postive |
| PDCD1 | PCED1B-AS1 | 0.709788 | 9.31E-84 | postive |
| TNFRSF25 | AC114730.3 | 0.710106 | 7.29E-84 | postive |
| VTCN1 | ZNF350-AS1 | 0.710254 | 6.50E-84 | postive |
| TNFRSF8 | AC117402.1 | 0.71047 | 5.50E-84 | postive |
| TNFRSF9 | LINC00426 | 0.710581 | 5.05E-84 | postive |
| TMIGD2 | AC010618.3 | 0.710732 | 4.49E-84 | postive |
| TNFRSF9 | TRG-AS1 | 0.711205 | 3.12E-84 | postive |
| TMIGD2 | MAFTRR | 0.711528 | 2.43E-84 | postive |
| TNFRSF8 | CTD-2201I18.1 | 0.711768 | 2.01E-84 | postive |
| TNFRSF25 | AC087741.1 | 0.712118 | 1.54E-84 | postive |
| PDCD1 | AC004585.1 | 0.712543 | 1.10E-84 | postive |
| TMIGD2 | AC068888.2 | 0.712594 | 1.06E-84 | postive |
| TMIGD2 | AL356488.3 | 0.712785 | 9.14E-85 | postive |
| TMIGD2 | AC016876.3 | 0.713637 | 4.70E-85 | postive |
| TMIGD2 | AC006160.1 | 0.713725 | 4.39E-85 | postive |
| TNFRSF14 | GUSBP11 | 0.714026 | 3.47E-85 | postive |
| TMIGD2 | AC000068.1 | 0.714288 | 2.82E-85 | postive |
| TMIGD2 | AL163051.1 | 0.715706 | 9.24E-86 | postive |
| TMIGD2 | AP003555.3 | 0.716338 | 5.61E-86 | postive |
| TNFRSF17 | AC007384.1 | 0.71692 | 3.54E-86 | postive |
| TNFRSF25 | AL135999.1 | 0.71695 | 3.45E-86 | postive |
| TNFRSF25 | AC005387.2 | 0.717137 | 2.98E-86 | postive |
| TMIGD2 | AC009955.3 | 0.717307 | 2.60E-86 | postive |

| | | | | |
|---|---|---|---|---|
| TMIGD2 | TCF4-AS2 | 0.717733 | 1.85E-86 | postive |
| CTLA4 | AC009133.3 | 0.717835 | 1.71E-86 | postive |
| TMIGD2 | GNA14-AS1 | 0.718437 | 1.06E-86 | postive |
| LTA | AC007728.2 | 0.718901 | 7.30E-87 | postive |
| TNFRSF10A | TNFRSF10A-AS1 | 0.718926 | 7.15E-87 | postive |
| LTA | AL591468.1 | 0.719082 | 6.31E-87 | postive |
| TMIGD2 | AC245014.3 | 0.719411 | 4.85E-87 | postive |
| TMIGD2 | AP001372.1 | 0.719906 | 3.26E-87 | postive |
| TMIGD2 | AP001178.1 | 0.719937 | 3.18E-87 | postive |
| FASLG | USP30-AS1 | 0.720178 | 2.62E-87 | postive |
| TNFRSF9 | AL135818.1 | 0.720525 | 1.98E-87 | postive |
| TMIGD2 | AC005911.1 | 0.720807 | 1.58E-87 | postive |
| TNFRSF25 | H1FX-AS1 | 0.721008 | 1.34E-87 | postive |
| TNFRSF25 | AC004148.2 | 0.721307 | 1.05E-87 | postive |
| TMIGD2 | SKAP1-AS1 | 0.721453 | 9.35E-88 | postive |
| TMIGD2 | RBM15-AS1 | 0.721839 | 6.84E-88 | postive |
| TMIGD2 | LINC00551 | 0.722073 | 5.66E-88 | postive |
| TMIGD2 | AL645728.1 | 0.722302 | 4.70E-88 | postive |
| CTLA4 | AC243960.1 | 0.72275 | 3.26E-88 | postive |
| TMIGD2 | AC007993.2 | 0.722755 | 3.25E-88 | postive |
| LTA | AC004865.2 | 0.722822 | 3.08E-88 | postive |
| TMIGD2 | GTF3C2-AS1 | 0.72317 | 2.32E-88 | postive |
| TNFRSF9 | AC243829.2 | 0.723665 | 1.55E-88 | postive |
| TNFRSF25 | AL022328.2 | 0.723704 | 1.50E-88 | postive |
| TMIGD2 | AC087878.1 | 0.724074 | 1.11E-88 | postive |
| ICOS | AC007728.2 | 0.724104 | 1.08E-88 | postive |
| TMIGD2 | AC130324.1 | 0.724252 | 9.57E-89 | postive |
| FASLG | AL591468.1 | 0.724264 | 9.48E-89 | postive |
| TNFRSF8 | AC017076.1 | 0.72427 | 9.43E-89 | postive |
| TMIGD2 | F10-AS1 | 0.724634 | 7.00E-89 | postive |
| TMIGD2 | AC021755.2 | 0.724824 | 5.99E-89 | postive |
| TNFRSF25 | AC012645.4 | 0.725249 | 4.22E-89 | postive |
| TMIGD2 | AP000350.5 | 0.725359 | 3.86E-89 | postive |
| TMIGD2 | AC124017.1 | 0.725396 | 3.74E-89 | postive |
| TMIGD2 | AC090061.1 | 0.725461 | 3.54E-89 | postive |
| CTLA4 | AC007728.2 | 0.725603 | 3.15E-89 | postive |
| TMIGD2 | AC117490.2 | 0.725657 | 3.02E-89 | postive |
| TMIGD2 | AC006441.4 | 0.725942 | 2.38E-89 | postive |
| TMIGD2 | AL021707.7 | 0.726471 | 1.54E-89 | postive |
| TNFRSF17 | FAM30A | 0.727086 | 9.25E-90 | postive |
| TMIGD2 | AL355001.1 | 0.727136 | 8.88E-90 | postive |
| TMIGD2 | AC140847.2 | 0.727397 | 7.15E-90 | postive |
| TMIGD2 | AC093752.2 | 0.727565 | 6.21E-90 | postive |
| TMIGD2 | AP001099.1 | 0.727986 | 4.38E-90 | postive |
| TMIGD2 | FALEC | 0.728343 | 3.25E-90 | postive |
| TMIGD2 | AL133551.1 | 0.72889 | 2.06E-90 | postive |
| TMIGD2 | AC123768.2 | 0.729398 | 1.35E-90 | postive |
| TNFRSF25 | AC040162.3 | 0.731 | 3.49E-91 | postive |
| ICOS | AC004585.1 | 0.731083 | 3.26E-91 | postive |
| TNFRSF25 | LINC00342 | 0.731511 | 2.27E-91 | postive |
| FASLG | AC007728.2 | 0.732627 | 8.80E-92 | postive |
| TNFRSF9 | AC004585.1 | 0.732655 | 8.59E-92 | postive |
| TMIGD2 | AC010618.2 | 0.732868 | 7.17E-92 | postive |
| TMIGD2 | AP001972.4 | 0.733743 | 3.40E-92 | postive |
| TMIGD2 | MIS18A-AS1 | 0.734843 | 1.32E-92 | postive |
| TMIGD2 | AP002993.1 | 0.735081 | 1.08E-92 | postive |
| TMIGD2 | AC006111.2 | 0.735228 | 9.51E-93 | postive |

| | | | | |
|---|---|---|---|---|
| TMIGD2 | AC005363.2 | 0.735775 | 5.93E-93 | postive |
| TMIGD2 | AC011825.4 | 0.736081 | 4.56E-93 | postive |
| TNFRSF25 | AL109811.3 | 0.737026 | 2.01E-93 | postive |
| TMIGD2 | AC079322.1 | 0.737171 | 1.77E-93 | postive |
| TMIGD2 | AP005264.1 | 0.737874 | 9.61E-94 | postive |
| TNFRSF9 | AL591468.1 | 0.737927 | 9.18E-94 | postive |
| TMIGD2 | AL391069.2 | 0.738217 | 7.13E-94 | postive |
| FASLG | PCED1B-AS1 | 0.738275 | 6.78E-94 | postive |
| LTB | PCED1B-AS1 | 0.73913 | 3.21E-94 | postive |
| TMIGD2 | AL356218.1 | 0.739735 | 1.89E-94 | postive |
| TMIGD2 | AL512791.2 | 0.739744 | 1.87E-94 | postive |
| TMIGD2 | LINC01336 | 0.739797 | 1.79E-94 | postive |
| ICOS | AL591468.1 | 0.740209 | 1.24E-94 | postive |
| TMIGD2 | TUBA3FP | 0.740264 | 1.18E-94 | postive |
| TMIGD2 | AC006960.2 | 0.740751 | 7.72E-95 | postive |
| TMIGD2 | SPON1-AS1 | 0.740927 | 6.60E-95 | postive |
| TMIGD2 | AC093001.1 | 0.741144 | 5.45E-95 | postive |
| TMIGD2 | SLC25A30-AS1 | 0.74155 | 3.81E-95 | postive |
| TMIGD2 | AL031733.2 | 0.742768 | 1.29E-95 | postive |
| TMIGD2 | AC107993.1 | 0.743208 | 8.70E-96 | postive |
| TMIGD2 | AC131571.1 | 0.74479 | 2.11E-96 | postive |
| TMIGD2 | AP001781.1 | 0.74499 | 1.76E-96 | postive |
| CTLA4 | PCED1B-AS1 | 0.745043 | 1.68E-96 | postive |
| TMIGD2 | C6orf99 | 0.74613 | 6.30E-97 | postive |
| TMIGD2 | AC244093.3 | 0.746598 | 4.12E-97 | postive |
| TMIGD2 | AC068831.1 | 0.746715 | 3.71E-97 | postive |
| TMIGD2 | AL022476.1 | 0.746729 | 3.66E-97 | postive |
| TMIGD2 | AC145285.3 | 0.747267 | 2.24E-97 | postive |
| TMIGD2 | AL391095.3 | 0.747385 | 2.02E-97 | postive |
| TMIGD2 | AC090559.2 | 0.747748 | 1.45E-97 | postive |
| TMIGD2 | AC012186.2 | 0.747961 | 1.19E-97 | postive |
| TMIGD2 | AC138207.7 | 0.748926 | 4.93E-98 | postive |
| TMIGD2 | AC002550.2 | 0.748971 | 4.73E-98 | postive |
| TMIGD2 | Z97200.1 | 0.749171 | 3.93E-98 | postive |
| TMIGD2 | AC011443.1 | 0.74919 | 3.87E-98 | postive |
| TMIGD2 | RN7SL832P | 0.74925 | 3.66E-98 | postive |
| TMIGD2 | AC009831.3 | 0.750111 | 1.66E-98 | postive |
| TNFSF8 | AC002091.1 | 0.750118 | 1.65E-98 | postive |
| TMIGD2 | AC103719.1 | 0.750426 | 1.24E-98 | postive |
| TMIGD2 | AC007405.1 | 0.750815 | 8.65E-99 | postive |
| TNFRSF25 | AC027601.1 | 0.751104 | 6.61E-99 | postive |
| TMIGD2 | AC012360.1 | 0.751138 | 6.41E-99 | postive |
| TNFRSF25 | AC020907.4 | 0.751223 | 5.92E-99 | postive |
| TMIGD2 | AC024592.1 | 0.751368 | 5.18E-99 | postive |
| TMIGD2 | AL451085.1 | 0.751404 | 5.01E-99 | postive |
| LTA | USP30-AS1 | 0.75193 | 3.07E-99 | postive |
| LTA | AL590764.1 | 0.752525 | 1.76E-99 | postive |
| TMIGD2 | AC087164.1 | 0.752747 | 1.43E-99 | postive |
| TMIGD2 | AC008663.3 | 0.752865 | 1.28E-99 | postive |
| CD86 | LINC01094 | 0.753485 | ######## | postive |
| TMIGD2 | AC010422.2 | 0.753866 | ######## | postive |
| TMIGD2 | AC090115.1 | 0.754586 | ######## | postive |
| TMIGD2 | AC005391.1 | 0.754987 | ######## | postive |
| CTLA4 | TRG-AS1 | 0.755005 | ######## | postive |
| TMIGD2 | LINC00471 | 0.75501 | ######## | postive |
| TMIGD2 | ABCC5-AS1 | 0.755322 | ######## | postive |
| CD274 | AL359076.1 | 0.755931 | ######## | postive |

| | | | | |
|---|---|---|---|---|
| TMIGD2 | LINC01422 | 0.755948 | ######## | postive |
| TMIGD2 | AC005277.2 | 0.756516 | ######## | postive |
| FASLG | LINC00426 | 0.756728 | ######## | postive |
| TNFRSF25 | AC005306.1 | 0.757099 | ######## | postive |
| TMIGD2 | AD001527.1 | 0.757172 | ######## | postive |
| TMIGD2 | AC004839.1 | 0.758171 | ######## | postive |
| ICOS | BHLHE40-AS1 | 0.758324 | ######## | postive |
| TMIGD2 | AL109947.1 | 0.75834 | ######## | postive |
| ICOS | TRG-AS1 | 0.758358 | ######## | postive |
| TMIGD2 | AC004233.4 | 0.759075 | ######## | postive |
| TMIGD2 | FAM215A | 0.759216 | ######## | postive |
| LTA | AL135818.1 | 0.759293 | ######## | postive |
| TMIGD2 | LINC02585 | 0.75967 | ######## | postive |
| TMIGD2 | AC023983.1 | 0.760219 | ######## | postive |
| TMIGD2 | AC092756.1 | 0.760385 | ######## | postive |
| TMIGD2 | AC009145.2 | 0.760481 | ######## | postive |
| TMIGD2 | AC099518.1 | 0.761046 | ######## | postive |
| TMIGD2 | FRY-AS1 | 0.76172 | ######## | postive |
| TMIGD2 | AC008543.4 | 0.761821 | ######## | postive |
| TMIGD2 | AC026474.1 | 0.761928 | ######## | postive |
| TMIGD2 | AC005237.1 | 0.763073 | ######## | postive |
| TMIGD2 | NDUFB2-AS1 | 0.763255 | ######## | postive |
| CD40LG | LINC00892 | 0.763343 | ######## | postive |
| TNFRSF25 | AC011472.1 | 0.76338 | ######## | postive |
| TMIGD2 | LINC01655 | 0.764032 | ######## | postive |
| TMIGD2 | REV3L-IT1 | 0.764228 | ######## | postive |
| TMIGD2 | AC067817.2 | 0.764275 | ######## | postive |
| TMIGD2 | AC114939.1 | 0.764315 | ######## | postive |
| TMIGD2 | AC008764.6 | 0.764545 | ######## | postive |
| TMIGD2 | AC013553.3 | 0.764683 | ######## | postive |
| TMIGD2 | AC015849.1 | 0.764946 | ######## | postive |
| TMIGD2 | AC093462.1 | 0.76505 | ######## | postive |
| TMIGD2 | AC005828.4 | 0.765212 | ######## | postive |
| LTA | LINC00426 | 0.765307 | ######## | postive |
| TMIGD2 | AC004584.1 | 0.765726 | ######## | postive |
| TMIGD2 | AC091180.2 | 0.765859 | ######## | postive |
| TMIGD2 | AC005616.1 | 0.766597 | ######## | postive |
| TMIGD2 | AL589739.1 | 0.76665 | ######## | postive |
| TMIGD2 | AC022960.1 | 0.767081 | ######## | postive |
| TMIGD2 | AC008443.5 | 0.767125 | ######## | postive |
| TMIGD2 | AC003965.2 | 0.767516 | ######## | postive |
| TMIGD2 | AC138230.1 | 0.767696 | ######## | postive |
| TMIGD2 | AC007292.3 | 0.767906 | ######## | postive |
| TMIGD2 | LINC01290 | 0.768115 | ######## | postive |
| TMIGD2 | AC099521.1 | 0.768377 | ######## | postive |
| TMIGD2 | AL121983.2 | 0.768509 | ######## | postive |
| TMIGD2 | AC087301.1 | 0.769051 | ######## | postive |
| FASLG | AC243829.4 | 0.769619 | ######## | postive |
| TMIGD2 | AC010463.2 | 0.769644 | ######## | postive |
| TMIGD2 | AP000915.2 | 0.769813 | ######## | postive |
| ICOS | AL135818.1 | 0.769936 | ######## | postive |
| TMIGD2 | AC005884.2 | 0.770183 | ######## | postive |
| CTLA4 | AC004585.1 | 0.770307 | ######## | postive |
| TMIGD2 | AL592071.1 | 0.770382 | ######## | postive |
| TMIGD2 | AC090970.1 | 0.770804 | ######## | postive |
| TMIGD2 | LINC02453 | 0.770939 | ######## | postive |
| TMIGD2 | HMGA1P4 | 0.770971 | ######## | postive |

| | | | | |
|---|---|---|---|---|
| TMIGD2 | AL513329.1 | 0.771554 | ######## | postive |
| TMIGD2 | AC137630.2 | 0.771781 | ######## | postive |
| TMIGD2 | AL135790.1 | 0.771926 | ######## | postive |
| TMIGD2 | AC100793.2 | 0.771961 | ######## | postive |
| TMIGD2 | AC105020.5 | 0.772259 | ######## | postive |
| TMIGD2 | AC096540.1 | 0.77229 | ######## | postive |
| TMIGD2 | AC008543.3 | 0.773145 | ######## | postive |
| TMIGD2 | AC138811.1 | 0.773236 | ######## | postive |
| TMIGD2 | LINC01635 | 0.773719 | ######## | postive |
| TMIGD2 | AL121890.2 | 0.77418 | ######## | postive |
| TMIGD2 | AC083841.1 | 0.774299 | ######## | postive |
| TMIGD2 | AP003555.2 | 0.774478 | ######## | postive |
| TMIGD2 | AC090229.1 | 0.774635 | ######## | postive |
| TMIGD2 | AL391832.2 | 0.774665 | ######## | postive |
| TMIGD2 | LINC02435 | 0.77518 | ######## | postive |
| TMIGD2 | AC007272.1 | 0.775363 | ######## | postive |
| TMIGD2 | AL591178.1 | 0.77571 | ######## | postive |
| TMIGD2 | AL161668.3 | 0.77583 | ######## | postive |
| TMIGD2 | AL031289.1 | 0.775959 | ######## | postive |
| TMIGD2 | UFL1-AS1 | 0.775989 | ######## | postive |
| TMIGD2 | LINC01985 | 0.776543 | ######## | postive |
| TMIGD2 | AC092718.6 | 0.776628 | ######## | postive |
| TMIGD2 | AC005529.1 | 0.776993 | ######## | postive |
| TMIGD2 | GK-IT1 | 0.777101 | ######## | postive |
| TMIGD2 | AC013549.1 | 0.777723 | ######## | postive |
| TMIGD2 | AC079384.1 | 0.777919 | ######## | postive |
| TMIGD2 | AC124947.1 | 0.777999 | ######## | postive |
| TMIGD2 | LINC02147 | 0.77823 | ######## | postive |
| TMIGD2 | AL109955.1 | 0.778958 | ######## | postive |
| TMIGD2 | AL591848.2 | 0.778998 | ######## | postive |
| TMIGD2 | AC025176.1 | 0.779201 | ######## | postive |
| TMIGD2 | AC004923.4 | 0.779989 | ######## | postive |
| TMIGD2 | AC091180.4 | 0.780265 | ######## | postive |
| TMIGD2 | AC017006.2 | 0.780484 | ######## | postive |
| TMIGD2 | LINC00449 | 0.78096 | ######## | postive |
| TMIGD2 | AC006111.3 | 0.781389 | ######## | postive |
| TMIGD2 | BRWD1-AS1 | 0.781416 | ######## | postive |
| TMIGD2 | AC005775.1 | 0.781519 | ######## | postive |
| FASLG | AL135818.1 | 0.781562 | ######## | postive |
| TMIGD2 | AC010531.6 | 0.781745 | ######## | postive |
| TMIGD2 | LIF-AS1 | 0.781755 | ######## | postive |
| TMIGD2 | AL360182.2 | 0.782045 | ######## | postive |
| TMIGD2 | AC008127.1 | 0.782217 | ######## | postive |
| TMIGD2 | AC011603.2 | 0.782286 | ######## | postive |
| ICOS | AC243829.4 | 0.782546 | ######## | postive |
| TMIGD2 | AC023813.3 | 0.782714 | ######## | postive |
| TMIGD2 | AC011471.2 | 0.782844 | ######## | postive |
| TMIGD2 | AC106870.1 | 0.783055 | ######## | postive |
| TMIGD2 | AC010525.1 | 0.783103 | ######## | postive |
| TMIGD2 | AL360157.1 | 0.783407 | ######## | postive |
| TMIGD2 | AL732314.4 | 0.78366 | ######## | postive |
| TMIGD2 | AL133371.3 | 0.784085 | ######## | postive |
| TMIGD2 | AC136469.1 | 0.784247 | ######## | postive |
| TMIGD2 | AC078777.1 | 0.784515 | ######## | postive |
| TMIGD2 | FO393418.1 | 0.78459 | ######## | postive |
| TMIGD2 | AC012363.2 | 0.784727 | ######## | postive |
| TMIGD2 | AL359636.1 | 0.784783 | ######## | postive |

| | | | | |
|---|---|---|---|---|
| TMIGD2 | LINC002481 | 0.785462 | ######## | postive |
| FASLG | AC004585.1 | 0.785551 | ######## | postive |
| TMIGD2 | LINC00618 | 0.7856 | ######## | postive |
| TMIGD2 | MDS2 | 0.785773 | ######## | postive |
| TMIGD2 | AP005203.1 | 0.785904 | ######## | postive |
| TMIGD2 | AC022167.3 | 0.785999 | ######## | postive |
| TMIGD2 | HCG14 | 0.786006 | ######## | postive |
| TMIGD2 | AP000944.1 | 0.786287 | ######## | postive |
| TMIGD2 | AC130469.1 | 0.786299 | ######## | postive |
| TMIGD2 | AC105020.2 | 0.786637 | ######## | postive |
| TMIGD2 | AC114321.1 | 0.786899 | ######## | postive |
| CD86 | AC011899.2 | 0.786913 | ######## | postive |
| TMIGD2 | AC007422.2 | 0.786917 | ######## | postive |
| TMIGD2 | AC011498.1 | 0.786979 | ######## | postive |
| TMIGD2 | AL358214.1 | 0.7871 | ######## | postive |
| TMIGD2 | AC008115.1 | 0.787209 | ######## | postive |
| TMIGD2 | AC084026.2 | 0.787239 | ######## | postive |
| LTA | TRG-AS1 | 0.787365 | ######## | postive |
| TMIGD2 | LINC02345 | 0.787503 | ######## | postive |
| TMIGD2 | AL023803.2 | 0.787558 | ######## | postive |
| TMIGD2 | AC118755.1 | 0.78762 | ######## | postive |
| TMIGD2 | AC020917.2 | 0.787917 | ######## | postive |
| TMIGD2 | AL391097.2 | 0.787939 | ######## | postive |
| TMIGD2 | AC012640.1 | 0.788027 | ######## | postive |
| ICOS | LINC00426 | 0.788032 | ######## | postive |
| TMIGD2 | AC011462.3 | 0.788324 | ######## | postive |
| TMIGD2 | AC078980.1 | 0.788454 | ######## | postive |
| TMIGD2 | AC093206.1 | 0.788523 | ######## | postive |
| TMIGD2 | LINC00337 | 0.788695 | ######## | postive |
| TMIGD2 | AC119396.2 | 0.788766 | ######## | postive |
| TMIGD2 | AC018761.1 | 0.78894 | ######## | postive |
| TMIGD2 | AL357497.1 | 0.789278 | ######## | postive |
| TMIGD2 | LINC01397 | 0.789379 | ######## | postive |
| TMIGD2 | AC013724.1 | 0.790129 | ######## | postive |
| TMIGD2 | AC005332.1 | 0.790319 | ######## | postive |
| TMIGD2 | AC135050.1 | 0.790584 | ######## | postive |
| LTA | BHLHE40-AS1 | 0.790718 | ######## | postive |
| TMIGD2 | LINC02356 | 0.790941 | ######## | postive |
| TMIGD2 | AP003774.4 | 0.791641 | ######## | postive |
| TMIGD2 | AC126773.1 | 0.791779 | ######## | postive |
| TMIGD2 | USP12-AS2 | 0.7918 | ######## | postive |
| TMIGD2 | AC009119.1 | 0.791872 | ######## | postive |
| TMIGD2 | AC008747.1 | 0.791962 | ######## | postive |
| TMIGD2 | LINC02340 | 0.792274 | ######## | postive |
| TMIGD2 | LINC02255 | 0.792396 | ######## | postive |
| TNFRSF17 | AC007569.1 | 0.793183 | ######## | postive |
| TMIGD2 | AC092809.2 | 0.794936 | ######## | postive |
| TMIGD2 | AC107294.1 | 0.795245 | ######## | postive |
| CTLA4 | LINC00426 | 0.795253 | ######## | postive |
| TMIGD2 | AC119428.2 | 0.799514 | ######## | postive |
| TMIGD2 | LINC01841 | 0.799885 | ######## | postive |
| TMIGD2 | AL355340.1 | 0.800486 | ######## | postive |
| TMIGD2 | AC091180.5 | 0.800936 | ######## | postive |
| TMIGD2 | MIR3945HG | 0.801755 | ######## | postive |
| TNFRSF25 | PTOV1-AS2 | 0.802602 | ######## | postive |
| TMIGD2 | AC025048.4 | 0.80548 | ######## | postive |
| FASLG | TRG-AS1 | 0.807079 | ######## | postive |

| | | | | |
|---|---|---|---|---|
| FASLG | BHLHE40-AS1 | 0.812391 | ######## | postive |
| TNFRSF25 | ARHGAP27P1-BPTFP1-KPNA2P3 | 0.815483 | ######## | postive |
| LTA | PCED1B-AS1 | 0.820093 | ######## | postive |
| TNFRSF17 | LINC02362 | 0.83749 | ######## | postive |
| TNFRSF17 | AC104699.1 | 0.846235 | ######## | postive |
| LTA | AC004585.1 | 0.847165 | ######## | postive |
| TNFRSF17 | AC012236.1 | 0.848246 | ######## | postive |
| TNFRSF9 | BHLHE40-AS1 | 0.850568 | ######## | postive |
| TNFRSF9 | AC243829.4 | 0.850662 | ######## | postive |
| TNFRSF17 | LINC00582 | 0.862059 | ######## | postive |
| TNFRSF17 | LINC02576 | 0.864258 | ######## | postive |
| TNFRSF17 | AL133467.1 | 0.873801 | ######## | postive |

**Table S3.** The identified 219 lncRNAs strongly associated with survival.

| lncRNA | HR | pvalue | lower | upper |
|---|---|---|---|---|
| AC024060.1 | 1.341182 | 1.92E-14 | 1.244082 | 1.445861 |
| AC016773.1 | 2.023985 | 5.12E-14 | 1.684586 | 2.431764 |
| AL354760.1 | 4.191864 | 9.90E-12 | 2.774729 | 6.332773 |
| BREA2 | 9.5322 | 8.94E-11 | 4.821666 | 18.84469 |
| HOTAIRM1 | 1.171586 | 4.92E-10 | 1.114572 | 1.231516 |
| AL513218.1 | 2.118174 | 6.40E-10 | 1.669508 | 2.687416 |
| RFPL3S | 10.08945 | 6.82E-10 | 4.841634 | 21.02535 |
| AP003352.1 | 1.565176 | 1.12E-09 | 1.355078 | 1.807849 |
| VPS9D1-AS1 | 1.889185 | 1.44E-09 | 1.537386 | 2.321485 |
| AL451050.2 | 6.621224 | 1.49E-09 | 3.58749 | 12.22041 |
| AL662797.1 | 4.879493 | 1.54E-09 | 2.917331 | 8.161383 |
| U62317.2 | 1.086782 | 1.63E-09 | 1.057784 | 1.116574 |
| AL137784.2 | 3.825858 | 2.10E-09 | 2.466316 | 5.934839 |
| LINC00941 | 1.456101 | 3.53E-09 | 1.28536 | 1.649522 |
| AL109741.1 | 1.117406 | 6.99E-09 | 1.076204 | 1.160187 |
| AC069281.2 | 2.531581 | 7.91E-09 | 1.84662 | 3.470612 |
| C3orf35 | 14.72375 | 8.27E-09 | 5.898949 | 36.7504 |
| AP002807.1 | 1.522066 | 1.09E-08 | 1.317876 | 1.757892 |
| AC095057.3 | 2.639981 | 1.86E-08 | 1.882282 | 3.702688 |
| AL121852.1 | 19.29166 | 3.89E-08 | 6.713913 | 55.43236 |
| AC004771.1 | 2.299699 | 5.10E-08 | 1.704316 | 3.103072 |
| AL117379.1 | 1.570123 | 5.28E-08 | 1.334632 | 1.847165 |
| AL139123.1 | 3.917873 | 6.18E-08 | 2.38964 | 6.423449 |
| AC068620.2 | 2.779059 | 6.79E-08 | 1.917275 | 4.028203 |
| PLA2G4C-AS1 | 9.109589 | 8.81E-08 | 4.054834 | 20.4656 |
| AL671710.1 | 9.25265 | 1.02E-07 | 4.078679 | 20.99002 |
| AL158834.2 | 8.099362 | 1.07E-07 | 3.744614 | 17.51841 |
| CAHM | 2.259953 | 1.11E-07 | 1.672447 | 3.053841 |
| AC048341.2 | 1.129568 | 1.35E-07 | 1.07955 | 1.181902 |
| AC093673.1 | 1.085291 | 1.39E-07 | 1.052729 | 1.118861 |
| AC083967.1 | 1.072794 | 1.79E-07 | 1.044863 | 1.101473 |
| LINC01943 | 1.893874 | 1.84E-07 | 1.48972 | 2.407672 |
| AC025857.2 | 1.217365 | 1.95E-07 | 1.130448 | 1.310964 |
| AC084824.5 | 1.563391 | 2.03E-07 | 1.320922 | 1.850367 |
| U47924.3 | 1.928488 | 2.14E-07 | 1.504664 | 2.471691 |
| AC147067.1 | 1.563475 | 2.50E-07 | 1.31928 | 1.852871 |
| LINC01311 | 2.364779 | 2.59E-07 | 1.704363 | 3.281095 |
| AC084876.1 | 1.600887 | 2.89E-07 | 1.337485 | 1.916163 |
| AC087289.2 | 6.999414 | 3.19E-07 | 3.319279 | 14.75977 |
| AC093726.2 | 1.708033 | 3.52E-07 | 1.390029 | 2.098788 |
| AC127502.2 | 1.326144 | 3.77E-07 | 1.189308 | 1.478723 |

| | | | | |
|---|---|---|---|---|
| SNHG10 | 1.505653 | 3.86E-07 | 1.285585 | 1.763393 |
| RRN3P2 | 2.799153 | 4.01E-07 | 1.880023 | 4.167639 |
| AC244197.2 | 1.494974 | 4.08E-07 | 1.279548 | 1.74667 |
| AL354836.1 | 1.082708 | 4.78E-07 | 1.049728 | 1.116723 |
| AL355488.1 | 1.316321 | 6.00E-07 | 1.181656 | 1.466333 |
| LINC01138 | 1.560393 | 6.62E-07 | 1.309379 | 1.859529 |
| LINC01355 | 1.689177 | 6.89E-07 | 1.373373 | 2.0776 |
| AL391244.3 | 1.821467 | 7.35E-07 | 1.436637 | 2.309381 |
| AC034236.2 | 1.790241 | 7.71E-07 | 1.421078 | 2.255304 |
| AC008870.2 | 2.435435 | 7.87E-07 | 1.710616 | 3.467375 |
| LINC01089 | 1.228902 | 8.36E-07 | 1.132152 | 1.33392 |
| AC078909.2 | 3.121222 | 8.43E-07 | 1.984282 | 4.909596 |
| AC009283.1 | 1.227119 | 9.18E-07 | 1.13082 | 1.331618 |
| AC027796.4 | 1.398539 | 9.87E-07 | 1.22275 | 1.5996 |
| AC010973.2 | 1.495941 | 1.21E-06 | 1.271395 | 1.760144 |
| AL731567.1 | 1.365812 | 1.24E-06 | 1.204099 | 1.549244 |
| AL390728.6 | 1.093914 | 1.38E-06 | 1.054774 | 1.134507 |
| ITGB2-AS1 | 1.224683 | 1.38E-06 | 1.127935 | 1.329729 |
| AL365356.5 | 1.212274 | 1.38E-06 | 1.121145 | 1.310811 |
| AC132192.2 | 1.717914 | 1.55E-06 | 1.377619 | 2.142268 |
| AL021707.6 | 1.22262 | 1.56E-06 | 1.126341 | 1.327128 |
| AC010245.2 | 2.261166 | 1.66E-06 | 1.619475 | 3.157118 |
| AC092119.2 | 2.019064 | 1.68E-06 | 1.514457 | 2.691803 |
| PTOV1-AS1 | 1.979176 | 1.69E-06 | 1.496625 | 2.617315 |
| AL645940.1 | 2.204145 | 1.73E-06 | 1.594378 | 3.047115 |
| AC016957.2 | 2.195277 | 2.06E-06 | 1.586761 | 3.037155 |
| AL596094.1 | 1.450299 | 2.13E-06 | 1.243666 | 1.691264 |
| AC104758.2 | 2.295796 | 2.15E-06 | 1.628024 | 3.237469 |
| AC092143.3 | 4.44237 | 2.21E-06 | 2.395689 | 8.237568 |
| AC092809.4 | 2.76864 | 2.56E-06 | 1.811166 | 4.232284 |
| AC005291.2 | 1.186013 | 2.65E-06 | 1.10451 | 1.273531 |
| AC005387.1 | 2.109825 | 2.66E-06 | 1.544883 | 2.881358 |
| AF196972.1 | 2.673959 | 2.81E-06 | 1.771831 | 4.035406 |
| LINC01160 | 6.3671 | 3.71E-06 | 2.906579 | 13.94766 |
| LINC02154 | 1.078374 | 3.83E-06 | 1.044405 | 1.113447 |
| AC073335.2 | 1.188252 | 4.26E-06 | 1.104026 | 1.278903 |
| AC127024.6 | 3.564889 | 4.71E-06 | 2.068574 | 6.143571 |
| AC100803.3 | 1.756522 | 4.80E-06 | 1.379769 | 2.236149 |
| LINC00926 | 1.667517 | 4.97E-06 | 1.338904 | 2.076784 |
| AC008105.1 | 10.94353 | 5.15E-06 | 3.911906 | 30.61448 |
| AC020907.4 | 1.418561 | 5.63E-06 | 1.219815 | 1.64969 |
| AC004034.1 | 4.329615 | 5.65E-06 | 2.299548 | 8.151848 |
| LINC00115 | 2.050203 | 6.33E-06 | 1.501216 | 2.79995 |
| MHENCR | 1.073955 | 6.41E-06 | 1.041184 | 1.107758 |
| RNF139-AS1 | 5.005759 | 6.50E-06 | 2.485687 | 10.08076 |
| AC124067.2 | 1.323223 | 7.65E-06 | 1.170458 | 1.495927 |
| AC120053.1 | 1.294781 | 9.20E-06 | 1.155085 | 1.451372 |
| AC010326.3 | 1.128736 | 9.57E-06 | 1.069811 | 1.190907 |
| AC020558.2 | 2.020305 | 9.77E-06 | 1.479286 | 2.75919 |
| YEATS2-AS1 | 2.300027 | 1.08E-05 | 1.58727 | 3.332845 |
| MIR155HG | 1.09785 | 1.10E-05 | 1.053104 | 1.144497 |
| AC002553.1 | 1.491395 | 1.11E-05 | 1.247887 | 1.78242 |
| LINC01871 | 1.193474 | 1.30E-05 | 1.102255 | 1.292241 |
| AL158212.2 | 1.965745 | 1.33E-05 | 1.450159 | 2.664641 |
| ASMTL-AS1 | 1.057749 | 1.34E-05 | 1.031346 | 1.084828 |
| AC015802.5 | 2.152651 | 1.35E-05 | 1.524148 | 3.040326 |
| AL591845.1 | 0.631507 | 1.40E-05 | 0.513242 | 0.777023 |

| | | | | |
|---|---|---|---|---|
| AC073655.2 | 2.81892 | 1.49E-05 | 1.763482 | 4.506035 |
| AC018809.1 | 3.852888 | 1.53E-05 | 2.090798 | 7.100041 |
| AC087741.1 | 1.381383 | 1.54E-05 | 1.193135 | 1.599332 |
| AL022328.3 | 1.926311 | 1.60E-05 | 1.430157 | 2.594591 |
| AC109446.3 | 2.107993 | 1.73E-05 | 1.500158 | 2.962111 |
| MCCC1-AS1 | 1.735534 | 1.78E-05 | 1.349205 | 2.232484 |
| AL592211.1 | 3.329944 | 1.78E-05 | 1.922262 | 5.768478 |
| AL359504.2 | 2.236264 | 1.92E-05 | 1.54606 | 3.234595 |
| AC027601.1 | 4.823705 | 1.92E-05 | 2.344101 | 9.926248 |
| ZKSCAN2-DT | 1.74007 | 1.99E-05 | 1.349047 | 2.244432 |
| AL035563.1 | 1.623795 | 2.13E-05 | 1.298559 | 2.03049 |
| AL139089.1 | 1.836313 | 2.54E-05 | 1.383856 | 2.436702 |
| AC005785.1 | 1.549768 | 2.71E-05 | 1.262991 | 1.901662 |
| AC010542.5 | 1.303755 | 2.85E-05 | 1.151449 | 1.476207 |
| AC010809.2 | 2.627525 | 2.86E-05 | 1.671218 | 4.131053 |
| AP000229.1 | 0.112662 | 2.89E-05 | 0.040496 | 0.313431 |
| ATP2C2-AS1 | 45.75301 | 2.97E-05 | 7.603491 | 275.3128 |
| CASC19 | 2.600579 | 3.11E-05 | 1.658611 | 4.077514 |
| CCDC18-AS1 | 1.173676 | 3.16E-05 | 1.08842 | 1.265611 |
| AC132872.1 | 1.196977 | 3.20E-05 | 1.099733 | 1.302818 |
| AC004951.4 | 19.4267 | 3.22E-05 | 4.797274 | 78.66901 |
| AL135818.2 | 4.522664 | 3.22E-05 | 2.220156 | 9.213087 |
| AC010327.5 | 6.389166 | 3.22E-05 | 2.66518 | 15.31658 |
| AC109460.3 | 4.138246 | 3.38E-05 | 2.114688 | 8.098159 |
| AC004825.2 | 1.203673 | 3.38E-05 | 1.102692 | 1.3139 |
| AL139352.1 | 4.752135 | 3.57E-05 | 2.269611 | 9.95007 |
| PICSAR | 1.334036 | 3.63E-05 | 1.163505 | 1.529562 |
| AL133410.1 | 1.867463 | 3.85E-05 | 1.387073 | 2.51423 |
| AC015660.3 | 1.998448 | 3.87E-05 | 1.437076 | 2.779111 |
| AC129510.1 | 1.622475 | 3.87E-05 | 1.288474 | 2.043058 |
| AL442128.2 | 6.733813 | 3.96E-05 | 2.711792 | 16.72113 |
| AC106786.1 | 3.680052 | 3.97E-05 | 1.976859 | 6.850659 |
| LINC02019 | 2.89566 | 3.97E-05 | 1.743909 | 4.808075 |
| AL096865.1 | 1.772026 | 4.17E-05 | 1.347814 | 2.329754 |
| IGBP1-AS1 | 2.936462 | 4.18E-05 | 1.754005 | 4.916069 |
| PTOV1-AS2 | 1.164799 | 4.18E-05 | 1.082832 | 1.25297 |
| AC132872.3 | 1.236176 | 4.21E-05 | 1.116901 | 1.368188 |
| AL596223.2 | 2.390392 | 4.49E-05 | 1.572834 | 3.632914 |
| LINC00893 | 1.557917 | 4.66E-05 | 1.258557 | 1.928484 |
| AC004817.3 | 1.109176 | 4.91E-05 | 1.055055 | 1.166073 |
| AC067945.3 | 2.883401 | 5.10E-05 | 1.72736 | 4.813126 |
| LINC02422 | 3.099021 | 5.71E-05 | 1.786469 | 5.375928 |
| AC099850.3 | 1.130629 | 6.20E-05 | 1.064697 | 1.200644 |
| ZNF436-AS1 | 1.393071 | 6.40E-05 | 1.184086 | 1.63894 |
| LINC00342 | 1.15099 | 7.00E-05 | 1.073909 | 1.233603 |
| AC048341.1 | 1.958167 | 7.04E-05 | 1.405854 | 2.727465 |
| AL096701.3 | 2.847558 | 7.30E-05 | 1.697852 | 4.77579 |
| AP001160.1 | 1.477487 | 7.33E-05 | 1.218247 | 1.791894 |
| AC092118.2 | 2.183981 | 7.36E-05 | 1.484302 | 3.213477 |
| PCED1B-AS1 | 1.130953 | 8.03E-05 | 1.063852 | 1.202286 |
| AC008105.3 | 1.30019 | 8.26E-05 | 1.140894 | 1.481728 |
| AL138921.1 | 8.739533 | 8.30E-05 | 2.969055 | 25.72517 |
| AL161452.1 | 3.493929 | 8.49E-05 | 1.872275 | 6.520164 |
| AC005306.1 | 2.438241 | 8.86E-05 | 1.561485 | 3.807284 |
| AC008764.8 | 2.104685 | 8.98E-05 | 1.450292 | 3.054349 |
| AC087239.1 | 1.704902 | 9.57E-05 | 1.304041 | 2.228986 |
| AC018653.3 | 1.28845 | 9.60E-05 | 1.134383 | 1.463442 |

| | | | | |
|---|---|---|---|---|
| AC073611.1 | 1.173598 | 9.70E-05 | 1.082839 | 1.271963 |
| AC022126.1 | 3.319327 | 9.83E-05 | 1.814884 | 6.070876 |
| LINC02195 | 1.612093 | 1.00E-04 | 1.267401 | 2.050531 |
| LINC02328 | 2.0932 | 0.000102 | 1.441998 | 3.038483 |
| LINC01011 | 1.651318 | 0.000108 | 1.28111 | 2.128507 |
| AC004148.2 | 1.284133 | 0.00011 | 1.131296 | 1.457618 |
| AC008610.1 | 1.123074 | 0.00011 | 1.058918 | 1.191118 |
| AC091057.1 | 3.310663 | 0.000117 | 1.800317 | 6.088089 |
| AC004817.2 | 1.12738 | 0.00012 | 1.060551 | 1.19842 |
| AC145285.6 | 6.35138 | 0.00013 | 2.463856 | 16.37272 |
| AC020931.1 | 7.303697 | 0.000139 | 2.626665 | 20.30864 |
| KMT2E-AS1 | 1.071451 | 0.000141 | 1.034036 | 1.110219 |
| AC005332.5 | 1.429961 | 0.000146 | 1.188973 | 1.719793 |
| AC022167.2 | 2.784666 | 0.000147 | 1.640999 | 4.725394 |
| AL359532.1 | 26.97521 | 0.000152 | 4.904861 | 148.3553 |
| PDXDC2P-NPIPB14P | 1.759616 | 0.000154 | 1.313169 | 2.357845 |
| AC009120.2 | 1.324227 | 0.000164 | 1.144308 | 1.532435 |
| AC017083.1 | 3.258824 | 0.000169 | 1.760776 | 6.031393 |
| AC074117.1 | 1.579307 | 0.000181 | 1.243335 | 2.006064 |
| AL513320.1 | 1.383192 | 0.000182 | 1.167128 | 1.639255 |
| LINC00174 | 1.266734 | 0.000186 | 1.119007 | 1.433963 |
| AL021707.8 | 1.287487 | 0.000188 | 1.127579 | 1.470073 |
| AP000254.1 | 1.263786 | 0.000195 | 1.117338 | 1.429428 |
| ASB16-AS1 | 1.273903 | 0.000203 | 1.121169 | 1.447444 |
| AL162171.1 | 0.323824 | 0.000205 | 0.178571 | 0.587229 |
| AC092171.4 | 1.461305 | 0.000207 | 1.195936 | 1.785557 |
| AC073575.2 | 3.105925 | 0.000209 | 1.706247 | 5.653797 |
| CAPN10-DT | 1.919864 | 0.000211 | 1.359732 | 2.710738 |
| NALT1 | 1.396845 | 0.000246 | 1.168279 | 1.67013 |
| ZNF32-AS1 | 2.210061 | 0.000263 | 1.443513 | 3.38367 |
| AC044849.1 | 3.058156 | 0.0003 | 1.668243 | 5.60609 |
| AC008735.2 | 1.101553 | 0.000317 | 1.045062 | 1.161098 |
| AC004816.1 | 1.136354 | 0.000319 | 1.059948 | 1.218268 |
| AC005840.2 | 1.563857 | 0.000346 | 1.224107 | 1.997905 |
| AC011462.4 | 1.169802 | 0.000363 | 1.073177 | 1.275126 |
| AC013731.1 | 2.582473 | 0.000366 | 1.532529 | 4.351738 |
| AC090152.1 | 1.737255 | 0.000377 | 1.281258 | 2.35554 |
| AC012645.4 | 1.895119 | 0.000405 | 1.32979 | 2.700785 |
| DM1-AS | 1.748512 | 0.000407 | 1.282719 | 2.383448 |
| AC023908.3 | 6.111782 | 0.000422 | 2.234326 | 16.71818 |
| AL159169.2 | 2.510509 | 0.000432 | 1.50371 | 4.191403 |
| AC016737.1 | 2.622619 | 0.000432 | 1.533026 | 4.486636 |
| AC040162.3 | 2.45467 | 0.000459 | 1.485395 | 4.056432 |
| NARF-IT1 | 2.277602 | 0.000471 | 1.43585 | 3.612821 |
| AL136295.7 | 1.290888 | 0.00051 | 1.11778 | 1.490805 |
| AC234582.1 | 1.725495 | 0.000542 | 1.266683 | 2.350498 |
| AC004585.1 | 1.296772 | 0.000608 | 1.117725 | 1.5045 |
| AC017076.1 | 1.217268 | 0.000658 | 1.08708 | 1.363047 |
| AC004865.2 | 2.068496 | 0.000667 | 1.360933 | 3.143928 |
| INE1 | 1.302074 | 0.000694 | 1.117889 | 1.516606 |
| AL360181.2 | 1.192535 | 0.000706 | 1.077012 | 1.320449 |
| AC114730.3 | 1.876805 | 0.000752 | 1.301373 | 2.706677 |
| AC004466.1 | 8.448773 | 0.000758 | 2.440029 | 29.25448 |
| USP30-AS1 | 1.173883 | 0.000824 | 1.068621 | 1.289514 |
| AC010883.1 | 1.259792 | 0.000829 | 1.10025 | 1.442469 |
| OGFR-AS1 | 2.293901 | 0.00084 | 1.409066 | 3.734376 |
| LINC02062 | 1.451611 | 0.000855 | 1.166041 | 1.807119 |

| | | | | |
|---|---|---|---|---|
| FO393401.1 | 2.695725 | 0.000896 | 1.501527 | 4.839696 |
| LINC00551 | 0.001774 | 0.000897 | 4.22E-05 | 0.074556 |
| AL683807.1 | 1.366256 | 0.000928 | 1.135841 | 1.643413 |
| AC017104.1 | 1.97869 | 0.000933 | 1.320955 | 2.963927 |
| AC009118.3 | 1.693563 | 0.000958 | 1.238835 | 2.315204 |
| H1FX-AS1 | 1.889165 | 0.000983 | 1.294069 | 2.757923 |
| AL135999.1 | 1.434679 | 0.000988 | 1.157399 | 1.778386 |
